# SPRIZ (CCDC127) protein conserved in deuterostome animals is a rigid OMM–IMM tether that locally sets mitochondrial IMS volume

**DOI:** 10.1126/sciadv.aef3522

**Published:** 2026-10-14

**Authors:** Raman Shulinski, Roman Loban, Danny Farhat, Benjamín Cartes-Saavedra, Matthew J. Magda, Pavel Kotchetkov, Victoria Caminiti, Linda David, Yuri I. Wolf, Conor O’Dwyer, Morgan Fullerton, Yan Burelle, Jyh-Yeuan (Eric) Lee, Andrea Raimondi, Eugene V. Koonin, György Hajnóczky, Mary-Ellen Harper, Luca Pellegrini

**Affiliations:** ^1^ Graduate program in Biochemistry, Faculty of Medicine, University of Ottawa, Ottawa, ON, Canada.; ^2^ Mitochondria Biology Laboratory, Faculty of Medicine, University of Ottawa, Ottawa, ON, Canada.; ^3^ MitoCare Center, Department of Pathology and Genomic Medicine, Thomas Jefferson University, Philadelphia, PA, USA.; ^4^ Ottawa Institute for Systems Biology, Faculty of Medicine, University of Ottawa, Ottawa, ON, Canada.; ^5^ Graduate Program in Neurobiology, Faculty of Medicine, University of Ottawa, Ottawa, ON, Canada.; ^6^ Computational Biology Branch, Division of Intramural Research, National Library of Medicine, National Institutes of Health, Bethesda, MD, USA.; ^7^ Department of Biochemistry, Microbiology and Immunology, Faculty of Medicine, University of Ottawa, Ottawa, ON, Canada.; ^8^ Interdisciplinary School of Health Sciences, Faculty of Health Sciences, University of Ottawa, Ottawa, ON, Canada.; ^9^ Institute for Research in Biomedicine, Bellinzona, Switzerland.

## Abstract

The double-membrane nature of mitochondria generates an intermembrane volume through which ions, reactive oxygen species, and metabolites must flux to reach the matrix or cytosol, but whether this volume is a genetically specified structure remains unknown. We identify SPRIZ (spatial regulator of the intermembrane zone), the product of *Ccdc127*, a gene conserved across deuterostome animals. SPRIZ assembles into an ∼18-nanometer homodimeric tether anchored in the outer mitochondrial membrane (OMM) and inserted into the inner mitochondrial membrane (IMM) through vertebrate-specific hydrophobic loops, physically spacing the two membranes and setting local intermembrane volume. Cryo-electron tomography of native mitochondria revealed rod-like densities spanning the intermembrane space (IMS), consistent with SPRIZ’s predicted rigid coiled-coil architecture and membrane-spanning topology. In mouse liver, two-dimensional morphometry measured ∼3-fold expansion of IMS area with SPRIZ and ∼5.5-fold with lengthened mitochondrial artificial increaser of the IMS (MARTINI); 3D electron tomography confirmed MARTINI’s greater OMM–IMM separation and normalized IMS volume. This expansion was functionally validated by increased ^14^C-mannitol accessibility to the IMS and by impaired mitochondrial Ca^2+^ uptake. Constitutive *Ccdc127/SPRIZ* knockout produced viable mice and, in liver mitochondria, preserved overall ultrastructure but caused modest IMS expansion, consistent with loss of local OMM–IMM constraints that stabilize membrane geometry at discrete sites. We therefore identify IMS volume as a genetically encoded and tunable architectural parameter of vertebrate mitochondria. In this model, SPRIZ is a molecular spacing system that locally sets OMM–IMM separation, shaping the environment through which metabolites, ions, and signals diffuse and establishing local gradients within the IMS.

## INTRODUCTION

Mitochondria are double-membrane organelles whose outer and inner mitochondrial membranes (OMM and IMM) delimit the intermembrane space (IMS), a narrow, crowded aqueous compartment that serves as a transit and reaction space for protons, Ca^2+^, metabolites, reactive oxygen species (ROS), phospholipids, and proteins (*1*–*6*). The IMS’s confined, crowded geometry is expected to influence processes that depend on its volume (e.g., metabolite flux) and on the distance separating the two membranes (e.g., protein import into the IMM and matrix). Given the importance of these processes, one might expect IMS volume—or OMM-IMM separation—to be actively regulated. However, despite near-saturation genetic screening in yeast, *Caenorhabditis elegans*, and *Drosophila melanogaster*, no dedicated molecular machinery has been identified that actively specifies IMS geometry or volume. This indicates that, in these model organisms, the IMS can self-organize and be maintained or remodeled indirectly by existing mitochondrial components and that core mitochondrial functions do not require a dedicated machinery that sets OMM-IMM distance or IMS volume. In vertebrates, however, mitochondria acquired additional functions that accompanied the emergence of diverse tissues with specialized bioenergetic and signaling demands (*1*, *7*, *8*), alongside an expansion of the mitochondrial proteome: Whereas the human mitochondrial proteome comprises ∼1136 proteins (*9*), the *Saccharomyces cerevisiae* mitochondrial proteome contains ∼901 high-confidence proteins (*10*), an ∼26% increase, with several conserved pathways also incorporating vertebrate orthologs that have diverged substantially in sequence and/or domain architecture (*11*, *12*). In this context (*13*), a dedicated molecular mechanism that locally controls IMS volume could represent a vertebrate-specific functional innovation, enabling precise regulation of intermembrane geometry to support vertebrate mitochondrial functions, tissue-specific metabolic demands, and physiological plasticity.

Although no dedicated spatial regulator of IMS volume has yet been identified, several mitochondrial assemblies can influence OMM-IMM apposition indirectly as a secondary consequence of their primary architectural roles. The most prominent is the mitochondrial contact site and cristae organizing system (MICOS), the core machinery that organizes cristae and forms cristae junctions (CJs), and which has therefore long been considered a potential contributor to OMM-IMM tethering (*14*, *15*). However, recent high-resolution cryo–electron tomography (cryo-ET) indicates that core MICOS subunits such as Mic60 and Mic19 are oriented predominantly parallel to the IMM rather than acting as perpendicular “struts” that could directly impose a defined intermembrane distance (*16*). MICOS can nevertheless engage outer-membrane partners through the mitochondrial IMS bridging (MIB) supercomplex, creating trans-envelope connections at CJ (*15*). Yet, there is currently no evidence that MICOS or MIB enforces—or dynamically regulates—IMS spacing and volume beyond these discrete junction-associated regions. Because CJs are interspersed along the boundary membrane (*12*), any spacing constraint generated at junctions would be expected to remain localized, whereas electron micrographs typically show a comparatively uniform, parallel OMM-IMM separation along extended stretches of the boundary membrane, including regions far from junctions. Consistent with this, careful inspection of representative electron micrographs from cell lines bearing single or double knockouts of MICOS components shows that despite profound disruption of cristae organization, a largely parallel and readily discernible OMM-IMM spacing along the boundary membrane remains evident (*17*). Together, these observations argue that MICOS/MIB-dependent contacts can account for local coupling at CJ but do not explain how IMS width and volume are specified more broadly along the boundary membrane, pointing to additional determinants of intermembrane geometry beyond MICOS/MIB.

Beyond MICOS/MIB, additional models have been proposed to account for intermembrane geometry, but none provide a general molecular explanation for how IMS width and volume are specified. Soluble IMS proteins have been suggested to influence OMM-IMM separation through steric effects, exemplified by mitochondrial creatine kinase (mtCK), which forms large oligomeric assemblies (*18*–*20*). However, mtCK expression is tissue-restricted (e.g., prominent in heart, skeletal muscle, and brain) and is undetectable in multiple large-scale mitochondrial proteomes, including mouse liver (*21*). This argues against a broadly deployed role for mtCK in setting IMS geometry and, more generally, supports the view that large IMS-resident proteins and complexes contribute to intermembrane geometry largely through indirect, “moonlighting” effects. ATAD3A provides a precedent for trans-envelope coupling and could affect intermembrane geometry, yet its adenosine triphosphatase–dependent activities and conformational dynamics support a multifunctional, regulated tether, again consistent with moonlighting contributions to spacing rather than a defining geometric function (*22*, *23*).

Collectively, these observations underscore a conceptual asymmetry in mitochondrial biology: Cristae architecture is a genetically encoded structural parameter of the cell because it is directly specified and remodeled by dedicated machineries. Instead, the IMS—from yeast to *Drosophila*—is not because it can be explained most parsimoniously through indirect and moonlighting effects rather than direct genetic specification. However, this paradigm may have shifted in vertebrate evolution. With the ∼26% mitochondrial proteome expansion and recruitment into broader tissue-specific functions and regulatory programs, there would have been substrate and selective pressure to acquire a dedicated molecular machinery that directly specifies IMS width and volume.

CCDC127 is a MitoCarta3.0 mitochondrial protein predicted to contain an elongated coiled-coil domain. Stimulated emission depletion (STED) microscopy studies showed CCDC127 signals appearing as puncta distributed across the mitochondrial network, without detectable overlap with MICOS markers or cristae-labeled Cox8a (*24*). Transfection-based studies proposed a peripheral, cytosolic-OMM association with roles in endoplasmic reticulum (ER)–mitochondria contacts and adipogenesis (*25*). By contrast, prior proximity-dependent biotin identification–based proteomics placed CCDC127 on the IMS side of the organelle (*26*), and a recent bioRxiv study further supports an OMM-anchored topology with the coiled-coil/C-terminal region exposed to the IMS under endogenous expression in human embryonic kidney (HEK) 293 cells (*27*). The functional role of CCDC127 remains unclear. A recent bioRxiv preprint proposes CCDC127 as an MICOS interactor in HEK293 cells, where its knockdown disrupts cristae organization (*27*). However, STED microscopy and CRISPR knockout studies in HeLa cells report no colocalization with Mic60, no proximity to CJ, and no cristae alterations (*24*). Additional bioRxiv preprints describe cristae disorganization upon CCDC127 knockdown in HeLa cells, linked to impaired lipid transport and reduced cardiolipin levels (*28*), or knockout in HEK293 cells, attributed to defective CCDC127 multimerization and dual OMM-IMM engagement via its C-terminal tips (*29*). These divergent findings and inconsistent mechanisms underscore the need for quantitative ultrastructural in vivo studies to elucidate the role of CCDC127 in mitochondrial structure.

Here, we conduct a bioinformatics analysis showing that, although *Ccdc127* most likely emerged early in bilaterian animals, it appears to have been lost in nearly all protostome taxa while remaining conserved across most deuterostomes. In particular, this protein is present in all vertebrates, where it shows a distinct domain architecture suggestive of a conserved function. Using in vivo constitutive *Ccdc127* knockout mice together with liver-directed adeno-associated virus serotype 8 (AAV8) expression (*21*), quantitative ultrastructural morphometry (*21*, *30*, *31*), mitochondrial proteomics (*32*), functional assays, and all-atom molecular dynamics (MD) simulations (*33*–*38*), we show in mouse liver that CCDC127 is a mitochondrial protein that homodimerizes into a rigid, ∼18-nm OMM–anchored tether spanning the IMS perpendicular to the two membranes; its C-terminal domain engages the IMM to expand IMS volume in a length-defined manner. Notably, spatial regulator of the intermembrane zone (SPRIZ)–dependent IMS expansion requires the SPRIZ loop residues that mediate engagement with the IMM. Whereas constitutive *Ccdc127* deletion did not detectably affect cristae structure or MICOS components in mouse liver, hepatic overexpression of SPRIZ caused limited cristae remodeling without detectably impairing mitochondrial respiration. On the basis of this causal architectural role, we refer to the *Ccdc127* gene product as SPRIZ, a vertebrate-conserved mechanism that establishes IMS width and volume as a genetically specified structural parameter of the cell.

## RESULTS

### Evolutionary analysis of SPRIZ (CCDC127): Bilaterian origin with vertebrate-specific retention and conservation

To place SPRIZ in an evolutionary context, we surveyed all National Center for Biotechnology Information eukaryotic genome assemblies satisfying high-quality assembly criteria, including reference genomes and complete or chromosome-level assemblies. This initial search space comprised 7025 eukaryotic genome assemblies. From these, we selected one representative assembly per eukaryotic family, generating a curated search space of 605 assemblies spanning 31 phyla. This analysis recovered 711 distinct SPRIZ homologs across 292 assemblies (data file S1). SPRIZ homologs were detected broadly across Deuterostomia, with the notable exception of Tunicata, and more sparsely in selected protostome lineages, including Bryozoa, Nemertea, Priapulida, and Arthropoda. Notably, SPRIZ homologs were not detected in the invertebrate genetic model organisms *D. melanogaster* and *C. elegans*.

Phylogenetic reconstruction revealed three strongly supported major clades corresponding to Chordata, non-chordate Deuterostomia, and Protostomia (fig. S1, A to C). This topology is best compatible with an origin of SPRIZ at the base of Bilateria, followed by near-complete conservation within Deuterostomia and multiple losses, or divergence beyond recognition, within Protostomia (table S1). An alternative scenario involving horizontal gene transfer from Deuterostomia to Protostomia appears less likely because the scattered distribution of SPRIZ homologs across Protostomia would require multiple independent transfer events, and documented gene transfers among animals are rare.

Within Chordata, the SPRIZ phylogeny largely followed the taxonomy and did not reveal distinct paralogous clades. Notably, the branch leading to the base of Vertebrata was very long, suggestive of rapid evolution accompanied by a defined structural innovation: insertion of an approximately 45–amino acid segment into the N-terminal coiled-coil region of ancestral SPRIZ, which therefore defines the length of the vertebrate SPRIZ form. Consistent with this interpretation, OrthoDB analysis identified SPRIZ orthologs in nearly every vertebrate species examined (1131 of 1155 species available; the absences are likely caused by incomplete or erroneous genome assembly), and the vertebrate consensus logo shows retention of this 45–amino acid segment (fig. S2, A and B). Together, these findings support the origin of SPRIZ at the base of Bilateria. The subsequent evolution of this protein was characterized by near-complete conservation within Deuterostomia, in particular, in vertebrates, following the acquisition of a vertebrate-specific extended coiled-coil domain, contrasted by sparse and scattered representation in Protostomia, likely due to multiple gene losses.

### Conserved length and membrane-binding features define the evolutionary conservation of SPRIZ (CCDC127) in vertebrates

Vertebrate SPRIZ orthologs exhibit pronounced primary-sequence conservation, with 49% identity between human and *Danio* SPRIZ and 57% between human and *Xenopus* SPRIZ, including preservation of an N-terminal hydrophobic segment encoding a transmembrane domain (discussed below; fig. S2A). AlphaFold3 predictions (*39*) further indicate that these orthologs adopt an essentially invariant, elongated α-helical architecture (Fig. 1A): human, bird, amphibian/reptile, and shark models overlay with minimal deviation and preserve a long (∼14 nm; ∼24 α-helical turns) coiled-coil segment of comparable length in the core domain, while the C-terminal region adopts a conserved fold that extends the overall span of the α-helical scaffold (Fig. 1A). This architecture resembles that found in nonvertebrate SPRIZ orthologs, e.g. horseshoe crab *Limulus polyphemus* (fig. S2, B and C). In *L. polyphemus*, however, the coiled-coil segment is about half the length of the counterpart in vertebrate SPRIZ owing to two internal deletions (20 and 14 amino acids) that together remove 44 residues (∼12 α-helical turns), shortening the predicted coiled-coil region (fig. S2, B to D). By contrast, the AlphaFold3-predicted C-terminal domain remains similar in overall fold despite only ∼24% sequence identity (fig. S2, B and C). Closer inspection, however, indicates that the protostome orthologs lack the two C-terminal features that enable vertebrate SPRIZ to autonomously graft into the IMM—loop-1 and loop-2 (shown below): While a loop-1–corresponding element is present, its sequence lacks the hydrophobic character required for membrane binding, and loop-2 is absent (fig. S2D). Together, these observations indicate that the defining features of vertebrate SPRIZ—a fixed ∼14-nm coiled-coil (∼24 α-helical turns) and two C-terminal IMM-grafting loops—emerged early in vertebrate evolution and have remained under strong purifying selection for ∼500 million years. Thus, SPRIZ function is predicted to depend both on the distinct protein architecture requiring length conservation and its capacity to engage the IMM.

**Fig. 1.**
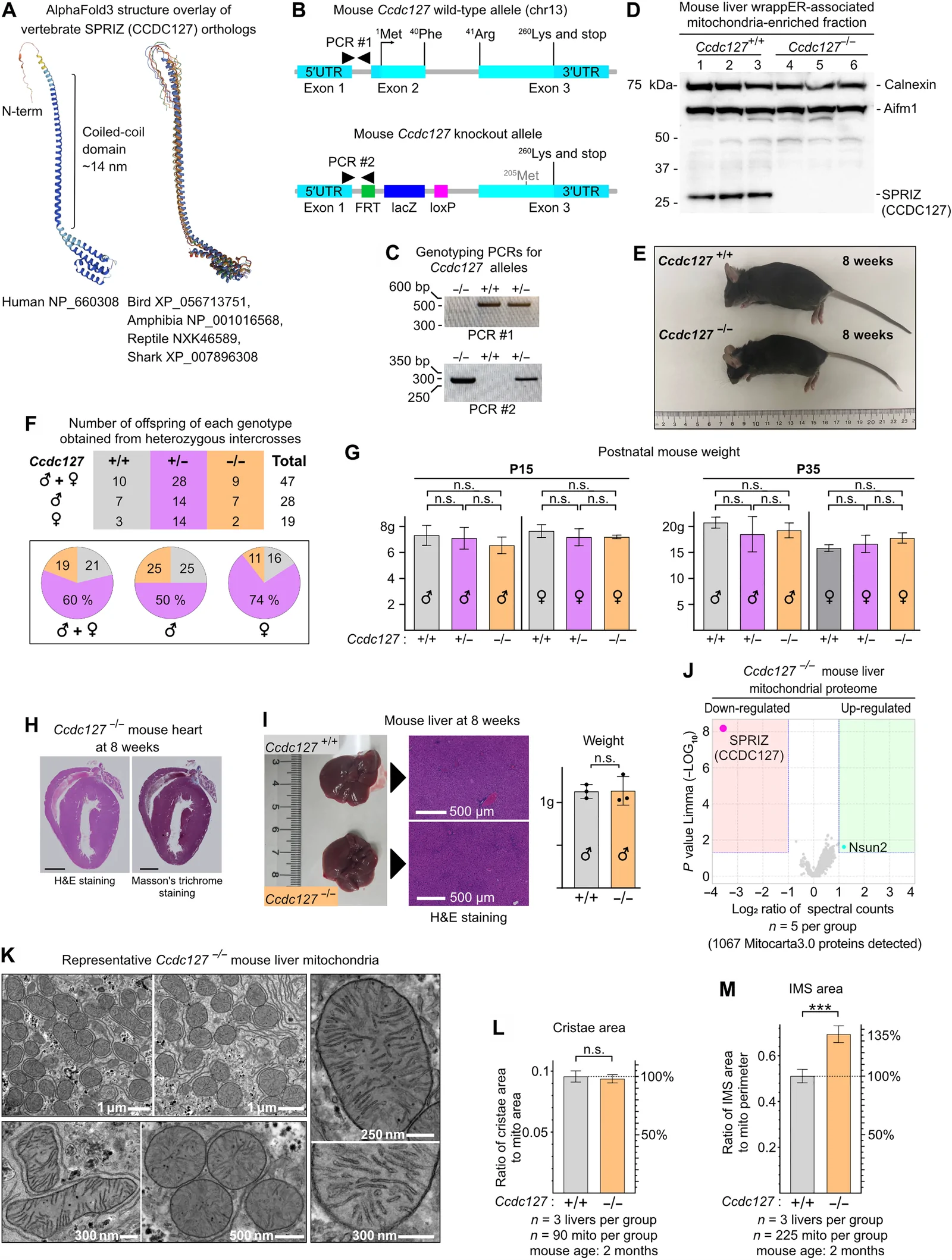
In *Ccdc127* knockout mice, loss of SPRIZ expands IMS area in liver mitochondria without changing mitochondrial content, cristae ultrastructure, or animal development. (**A**) AlphaFold3 overlays of SPRIZ (CCDC127). (**B**) Wild-type and knockout alleles on chromosome 13 (EMMA #14537). Exon 2, containing the 5′UTR, start codon, and first 40 amino acids, is deleted; FRT (green), lacZ (blue), and loxP (magenta) sites are shown. (**C**) Polymerase chain reaction (PCR) #1 detects wild-type [570 base pairs (bp)]; PCR#2 identifies heterozygous (both bands), and homozygous knockout (305 bp). (**D**) SPRIZ immunoblot in wrappER-associated mitochondrial fractions from wild-type (+/+) and knockout (−/−) livers, confirming loss. (**E**) Eight-week-old wild-type and knockout littermates. (**F**) Genotypes from heterozygous intercrosses (total *n* = 47 offspring) did not differ from 1:2:1 (χ^2^ = 1.77, df = 2, *P* = 0.41). (**G**) Body weight at P15 and P35 by genotype and sex (*n* ≥ 7 per group; n.s., not significant by Kruskal-Wallis test; data are shown as means ± SD). (**H**) Knockout heart sections stained with H&E and Masson’s trichrome (scale bars, 1.5 mm). (**I**) Liver images, liver weight (*n* = 3 per group; n.s., not significant by Mann-Whitney *U* test), and H&E sections (scale bars, 500 μm). (**J**) Mitochondrial proteomics volcano plot (control versus *Ccdc127* knockout; *n* = 5 per group), showing SPRIZ loss without major mitochondrial protein changes (thresholds at *P* < 0.05 and |log_2_ fold change| >1). (**K**) Knockout liver mitochondrial electron micrographs showing preserved ultrastructure and cristae. (**L**) Cristae area/mitochondrial area; workflow and points are in fig. S6D (*n* = 3 livers per group, 90 mitochondria per group analyzed; n.s., not significant by unpaired *t* test; data are shown as means ± SEM). (**M**) IMS area/mitochondrial perimeter; workflow and points are in fig. S6E (***P < 0.001 by unpaired *t* test; data are shown as means ± SEM).

### SPRIZ (CCDC127) is an OMM-anchored protein with an IMS-facing coiled-coil domain

We confirmed the MitoCarta3.0 annotation of *Ccdc127* (*9*) by detecting SPRIZ in HeLa cells and mouse liver and validated the commercially available anti-CCDC127 antibody for use in this study (figs. S3, A and B and S4, A to C). Three independent predictors (TMHMM, Phobius, and AlphaFold3) identified a single conserved transmembrane helix (amino acids 26 to 44) (fig. S3, C and D), supported by carbonate extraction showing that SPRIZ is an integral membrane protein (fig. S3E). Protease-protection assays on intact liver mitochondria further established a short cytosolic N terminus, with the coiled-coil and C-terminal regions exposed to the IMS (fig. S3, F and G).

To define SPRIZ tissue distribution, we surveyed ProteomicsDB transcriptomic datasets, which showed that *Ccdc127* is expressed across mouse tissues, with highest expression in brain and substantial levels in heart, liver, kidney, and brown adipose tissue (fig. S5). In mouse liver RNA sequencing data (*21*), *Ccdc127* transcripts [∼13 transcripts per million (TPM)] fall within the range of mitochondrial functional regulators such as MFN1 (14 TPM), MFN2 (21 TPM), and OPA1 (29 TPM) but are far below highly abundant outer-membrane proteins or large structural assemblies such as VDAC1 (142 TPM) and MICOS components IMMT/MIC60 (79 TPM) and MIC19 (110 TPM) (table S2).

We next quantified SPRIZ in a mouse liver wrapping rough ER (wrappER)–associated mitochondria-enriched fraction proteomics dataset (data file S2) (*21*, *32*) using intensity-based absolute quantification (iBAQ), a metric that is approximately proportional to molar abundance within a dataset and is most appropriate for within-experiment comparisons across proteins. Across five independent samples from this analysis, SPRIZ averaged 345 iBAQ—comparable to MFN1 (171), MFN2 (395), and OPA1 (1,836), and orders of magnitude lower than VDAC1 (86,045), IMMT/MIC60 (7,992), and MIC19 (19,115) (table S2).

Independent protein copy-number estimates in HEK293 cells (*40*) similarly place SPRIZ at ∼30,984 molecules per cell, comparable to MFN2 (85,243) but far below VDAC1 (2,937,942) and MIC60 (974,301). Together with STED microscopy showing a punctate mitochondrial distribution of endogenous SPRIZ in HeLa cells (*24*), these data support the idea that SPRIZ acts at discrete sites within the IMS.

### SPRIZ (CCDC127) affects IMS geometry without affecting mouse development or viability

These observations raised the possibility that SPRIZ represents a previously unrecognized class of mitochondrial factor: a protein that contributes to the spatial organization of the intermembrane compartment. To test this model in vivo, we generated a constitutive *Ccdc127* knockout mouse as a genetic tool to examine the consequences of SPRIZ loss at the organismal, tissue, and mitochondrial ultrastructural levels.

We obtained a constitutive *Ccdc127* knockout mouse line from the European Mouse Mutant Archive consortium (EMMA #14537) and rederived it for phenotypic assessment. The knockout allele disrupts the *Ccdc127* open reading frame by deleting exon 2, which contains part of the 5′ untranslated region (5′UTR), the initiating methionine, and the first 40 amino acids; the next in-frame AUG occurs at Met^205^ and would produce at most a short ∼55–amino-acid peptide lacking the predicted transmembrane helix (Fig. 1, B and C). Immunoblotting of liver wrappER–associated mitochondria–enriched fractions confirmed complete absence of SPRIZ in homozygous *Ccdc127* knockout animals without altering levels of representative mitochondrial or ER markers (Fig. 1D). Homozygous knockouts arose at expected Mendelian ratios from heterozygous intercrosses, with no embryonic or perinatal lethality and no sex bias (Fig. 1F). Moreover, *Ccdc127^−/−^* males and females readily produced viable litters including both sexes. Postnatally, *Ccdc127^−/−^* mice appeared indistinguishable from wild-type littermates in size and gross morphology at 8 weeks of age (Fig. 1E), and no overt behavioral abnormalities were observed under standard housing conditions. Body weights remained comparable across genotypes and sexes at postnatal days 15 (P15) and P35 (Fig. 1G).

Representative hematoxylin and eosin (H&E) and Masson’s trichrome staining of hearts from 8-week-old knockout mice showed preserved cardiac morphology with no overt pathology or fibrosis (Fig. 1H). At the same age, male liver weights were unchanged and H&E staining revealed normal hepatic architecture (Fig. 1I). Because grossly normal histology can, in principle, mask compensatory changes at the organelle level, we next examined the proteomes of the two organelle systems most directly positioned to buffer hepatic metabolic perturbations linked to *Ccdc127* ablation: mitochondria and their tightly associated wrappER, which ensheathes liver mitochondria and is implicated in lipoprotein synthesis and hepatic lipid metabolism (*21*, *30*, *40*). We therefore performed comparative liquid chromatography–tandem mass spectrometry on five wrappER-associated mitochondria–enriched fractions prepared as described (*32*) from livers of 8-week-old *Ccdc127^+/+^* and *Ccdc127^−/−^* mice (data file). Among 1067 MitoCarta3.0-annotated mitochondrial proteins quantified, SPRIZ was markedly depleted in knockouts—validating genetic ablation—whereas no other mitochondrial proteins were significantly altered aside from a modest up-regulation of NSUN2 (Fig. 1J). Similarly, analysis of wrappER proteins revealed no significant changes (fig. S6A).

Consistent with the mitochondrial proteomic data, electron microscopy (EM) analyses revealed no overt differences in mitochondrial appearance in *Ccdc127^−/−^* mouse livers (Fig. 1K and fig. S6F). Quantitative EM morphometry of three independent livers per genotype further demonstrated no changes in mitochondrial abundance or average cross-sectional area based on analysis of approximately 13,000 mitochondria in total (fig. S6B). At the ultrastructural level, cristae width and area were likewise unchanged in knockout mitochondria (Fig. 1L and fig. S6, C and D). In contrast, the IMS area increased by approximately 35% in *Ccdc127^−/−^* liver mitochondria (Fig. 1M and fig. S6E; *P* < 0.001) based on measurements of 225 IMS regions per genotype across the three livers. Thus, under the tested basal conditions, SPRIZ is dispensable for normal viability, gross tissue architecture, mitochondrial abundance, and cristae organization, yet its loss selectively increases IMS area, identifying SPRIZ as a determinant of intermembrane geometry in vivo.

### SPRIZ (CCDC127) assembles into homodimers in mouse liver mitochondria

Bioinformatic analysis identified an α-helical region in SPRIZ (amino acids 113 to 134) organized as a coiled-coil with heptad repeats in a leucine zipper–like register, with hydrophobic a/d positions and charged e/g residues consistent with potential dimerization (Fig. 2A). AlphaFold3 predictions supported this architecture with high confidence (predicted local distance difference test ∼70 to 90 across the core), modeling SPRIZ as a parallel homodimer in which the coiled-coil domain forms the rod-like shaft and the C-terminal domain homodimerizes to generate a wider, conical module at the distal end (Fig. 2B and fig. S7H). This model is consistent with recent structural data on recombinant CCDC127 fragments, including dimerization/tetramerization of the coiled-coil domain (residues 48 to 120) by crystallography, homodimerization of the C-terminal domain (137 to 260) by size-exclusion chromatography, and transfection experiments with residues 1 to 135 (*29*). We therefore tested full-length SPRIZ self-association using orthogonal cellular and biochemical assays.

**Fig. 2.**
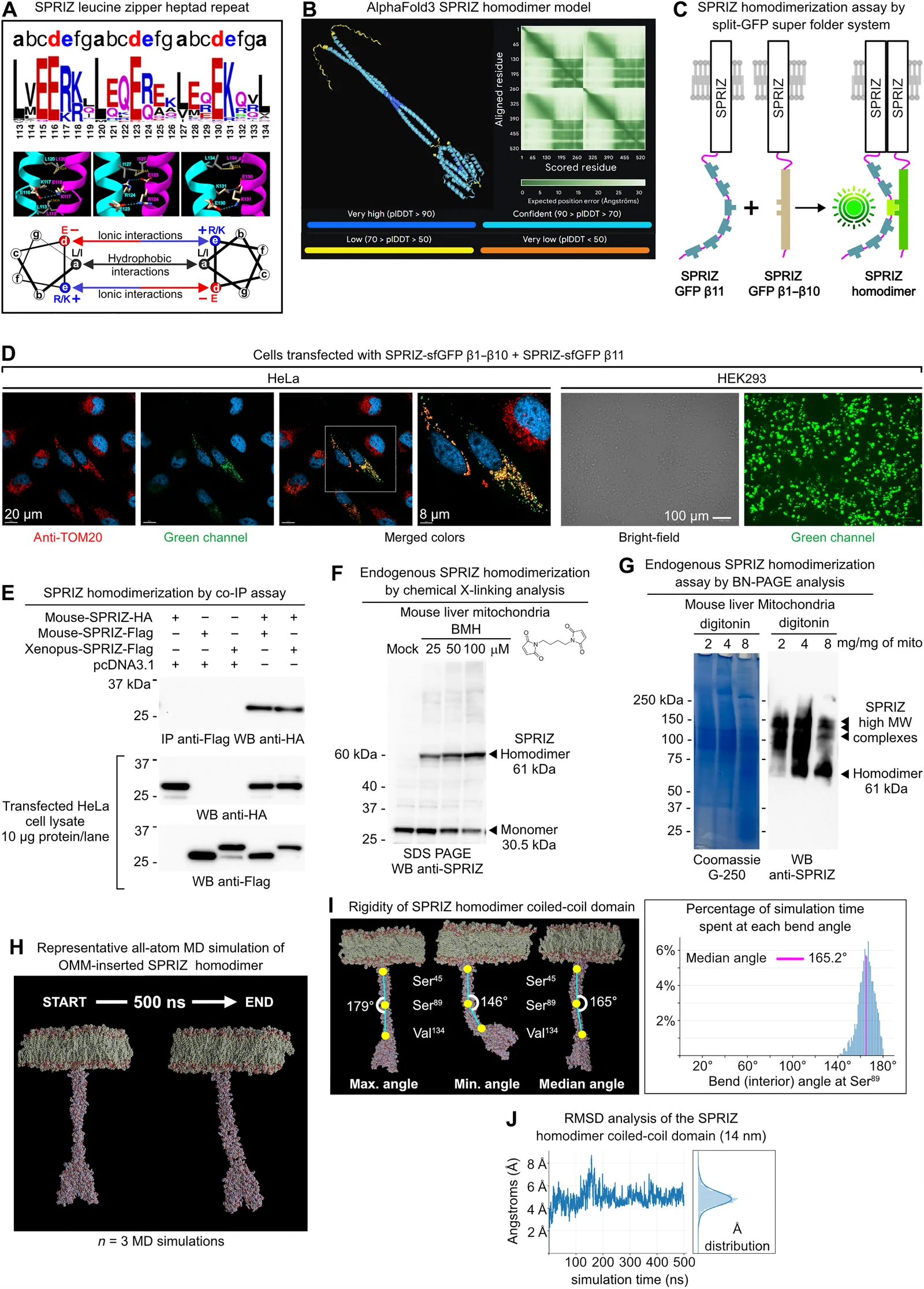
SPRIZ forms a rigid coiled-coil homodimer in endogenous mouse liver mitochondria. (**A**) Predicted leucine-zipper heptad repeats in SPRIZ (CCDC127), highlighting hydrophobic a/d positions and ionic interactions supporting coiled-coil dimerization. (**B**) AlphaFold3 model of the SPRIZ homodimer showing an elongated coiled-coil with high predicted local distance difference test (pLDDT) scores (>90) in the core and lower confidence at the termini. (**C**) Split-superfolder GFP assay in which SPRIZ fused to β1 to 10 and β11 fragments reconstitutes fluorescence upon coexpression (right), indicating homodimerization. (**D**) Confocal images of HeLa and HEK293 cells expressing SPRIZ-sfGFP(β1–10)–HA and SPRIZ-sfGFP(β11)x7-Flag, showing reconstituted GFP (green) colocalized with TOM20 (red); merged and bright-field images are shown. (**E**) Coimmunoprecipitation of SPRIZ-HA and SPRIZ-Flag from HeLa lysates showing reciprocal pull-down. (**F**) Endogenous SPRIZ homodimerization by bismaleimidohexane (BMH) cross-linking of mouse liver mitochondria-enriched fractions. BMH [0 (mock), 25, 50, or 100 μM; structure shown right] produced a prominent ∼61-kDa SPRIZ band (arrowhead), with reduction of the ∼30.5-kDa monomer. (**G**) BN-PAGE of mouse liver mitochondria solubilized with 2, 4, or 8 mg of digitonin per mg mitochondrial protein. Coomassie staining shows loading; anti-CCDC127 immunoblotting identifies an ∼61-kDa homodimer and higher–molecular weight SPRIZ complexes (arrowheads). (**H**) Starting and 500-ns end-point structures from an all-atom simulation of the SPRIZ homodimer in an OMM-like bilayer. (**I**) Coiled-coil rigidity during simulation. Snapshots show maximum (179°), minimum (146°), and median (165°) bend angles at Ser^89^; Ser^45^, Ser^89^, and Val^134^ are marked, and cyan lines indicate the helical axis. The angle distribution peaks at 165.2° (magenta line), indicating limited bending. (**J**) Backbone RMSD more than 500 ns stabilized at 4 to 6 Å after equilibration, with occasional peak to ∼8 Å. The inset distribution is centered at ∼4 to 5 Å, supporting overall conformational stability.

Split-superfolder green fluorescent protein (GFP) complementation detected homodimerization in two mammalian cell lines (HeLa and HEK293), with mitochondrial fluorescence observed only upon coexpression of complementary GFP-tagged SPRIZ constructs (Fig. 2, C and D, and fig. S7, A and B and I to J). Coimmunoprecipitation provided independent evidence for self-association (Fig. 2E and fig. S7, C to F) and also detected association between SPRIZ orthologs from mouse and *Xenopus* (Fig. 2E and fig. S7, E, G, and H). This cross-species association indicates that the structural determinants that specify SPRIZ dimerization are conserved across vertebrates, consistent with broad sequence conservation across the full-length protein and with AlphaFold3 models supporting a compatible mouse–*Xenopus* dimer interface (fig. S7, G and H).

To establish the oligomeric state of endogenous SPRIZ in its native physiological environment, we performed orthogonal biochemical analyses directly in mouse liver mitochondria. Chemical cross-linking with bismaleimidohexane (BMH) induced a clear, dose-dependent shift from monomer to an ∼61-kDa species corresponding to the SPRIZ homodimer (Fig. 2F). Progressive digitonin solubilization and blue native polyacrylamide gel electrophoresis (BN-PAGE) similarly revealed a prominent ∼61-kDa SPRIZ species consistent with the homodimer, together with additional higher–molecular weight SPRIZ-containing species whose composition remains to be determined (Fig. 2G). Together with recent structural studies on CCDC127 (*29*), these complementary in vivo approaches demonstrate that SPRIZ predominantly assembles as a homodimer under native mitochondrial conditions in mouse liver.

### All-atom MD simulations define SPRIZ (CCDC127) as a rigid coiled-coil rod with sustained near-perpendicular orientation to the OMM

To test whether the SPRIZ homodimer predicted by AlphaFold3 has the mechanical rigidity and long-term stability required to function as a spatial regulator of the IMS, we performed all-atom MD simulations of the full-length, membrane-anchored homodimer embedded in an OMM-mimetic phospholipid bilayer. Three independent 500-ns simulations showed no unfolding, dimer dissociation, or large-scale bending of the coiled-coil rod, and snapshots at the start and end of a representative trajectory confirmed sustained near-perpendicular orientation relative to the bilayer plane, with only modest conformational adjustments over time (Fig. 2H and movie S1).

Backbone root mean square deviation (RMSD) analysis revealed clear domain-specific behavior (fig. S8A and Fig. 2J). The coiled-coil domain (spanning residues 45 to 134) exhibited moderate fluctuations, with RMSD rising during initial equilibration and stabilizing around 5 to 6 Å over the latter half of the trajectory (Fig. 2J). Complementing this stability, quantitative assessment of backbone linearity in the coiled-coil—via interior bend angles along virtual lines connecting representative α-carbons (Ser^45^ → Ser^89^ → Val^134^)—showed tight clustering around a median of 165.2° (Fig. 2I), indicating strong resistance to sharp curvature and overall persistence as a near-straight rod (180° ideal).

In contrast, the C-terminal domain (residues 135 to 260) exhibited greater flexibility, with RMSD plateauing at ∼3 to 3.5 Å (fig. S8A). This mobility was corroborated by the broader bend angle distribution along Ser^89^ → Val^134^ → Phe^177^, centered at a median of 131.6° (fig. S8B), indicating substantial conformational variability in the distal module compared to the rigid coiled-coil rod. The sustained near-perpendicular, membrane-anchored architecture of the SPRIZ homodimer thus establishes a mechanistic foundation for direct regulation of IMS volume, with the rigid coiled-coil positioning the C-terminal conical domain ∼14 nm projected toward the IMM—where its moderate flexibility may act as a hinge-like joint to adapt to potential IMM undulations and facilitate targeted interactions with the IMM lipid bilayer, a hypothesis we tested next.

### Multiscale, orthogonal computational analyses define SPRIZ as an autonomous OMM-IMM tether

SPRIZ homodimer terminates in a C-terminal conical domain that, by virtue of its position and two vertebrate-specific hydrophobic elements at its base (loop-1 and loop-2; fig. S2D), is well placed to contact the opposing IMM. We therefore first tested whether this distal module can autonomously engage an IMM-like bilayer using three complementary in silico approaches: physics-based membrane positioning (PPM), AlphaFold3 protein–lipid modeling, and coarse-grained (CG) MD simulations.

PPM 3.0 positioned the SPRIZ homodimer against an IMM-mimicking bilayer with the base of the C-terminal conical domain contacting the headgroup region, placing loop-1 (176 to 178; Leu-Phe-Leu) and loop-2 (222 to 223; Val-Trp) at the interface (Fig. 3, A and B, and movie S2). AlphaFold3 protein–lipid modeling independently converged on the same binding mode, with loops 1 and 2 contributing prominently alongside additional residues near the distal surface (Fig. 3C and movie S3). Together, these models align with a recent bioRxiv report showing that a recombinant CCDC127 C-terminal homodimer binds liposomes in vitro and that Ser substitutions in the loop residues weaken this association (*29*).

**Fig. 3.**
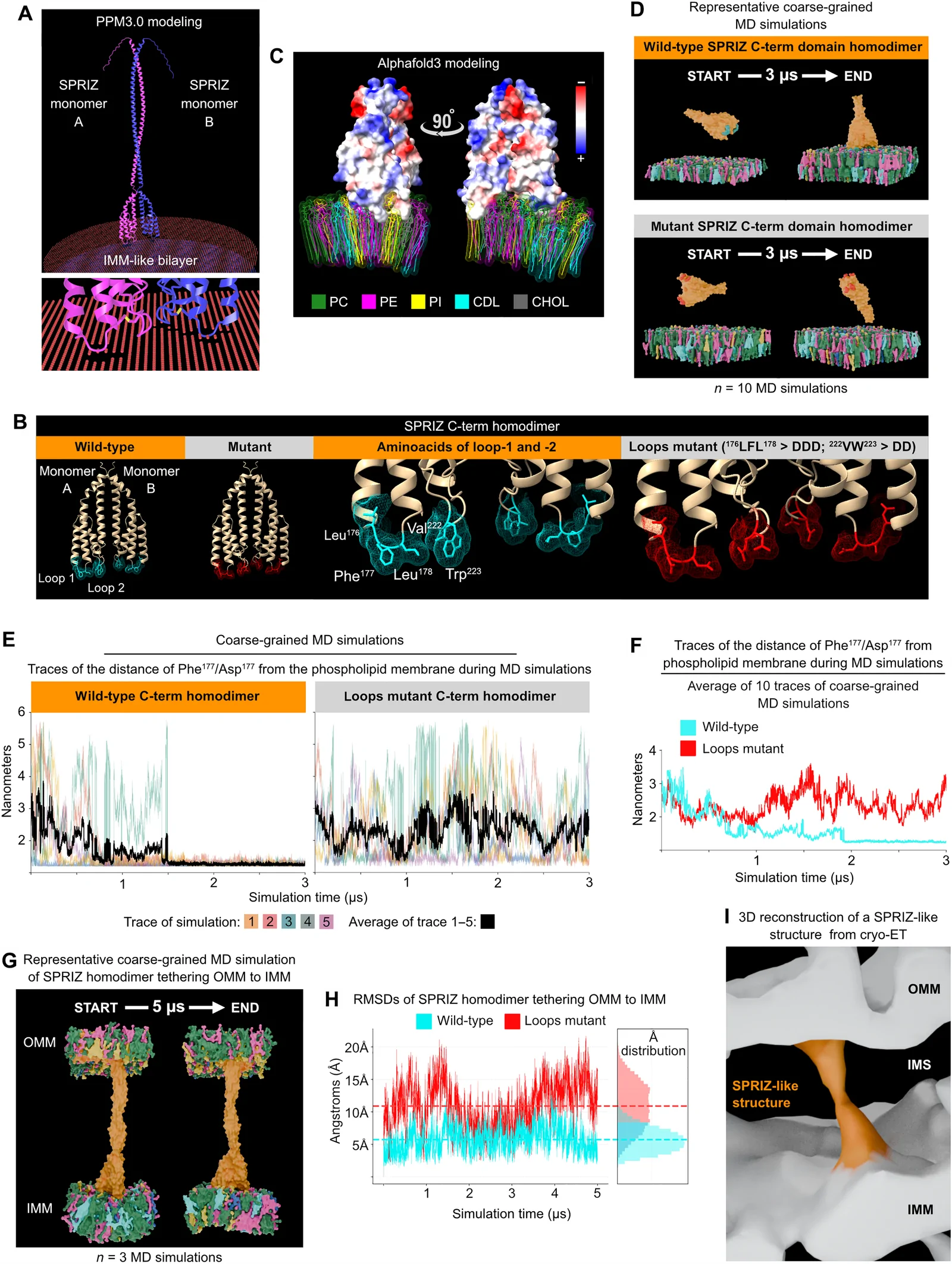
Multiscale modeling and CG MD simulations of SPRIZ homodimer membrane engagement and OMM-IMM tethering. (**A**) PPM 3.0 membrane-embedding model of the SPRIZ homodimer at an IMM-like bilayer. The parallel homodimer is nearly perpendicular to the membrane, with its C-terminal conical domain engaging the IMM surface. Bottom: enlarged interface showing two unstructured loops. (**B**) Structural basis of loop engagement and design of the charge-reversal “loops mutant.” Left: C-terminal conical homodimer with membrane-proximal loop-1 and loop-2 (cyan). Center: hydrophobic residues in loop-1 (L176, F177, and L178) and loop-2 (V222 and W223) of monomer A forming the predicted IMM-interacting interface. Right: loops mutant (176LFL → 176DDD; 222VW → 222DD), converting this surface to a negatively charged interface (red). (**C**) AlphaFold3 model of the C-terminal conical homodimer at an IMM-like lipid monolayer, shown as electrostatic surfaces in two orthogonal views. The loops extend below polar headgroups into the acyl-chain region. Lipids shown are PC, PE, PI, CDL, and CHOL; red and blue indicate negative and positive potential. The model used an earlier AlphaFold3 version supporting protein-lipid comodeling, unavailable in the current public version. Inputs, outputs, and models are provided in Supplementary Auxiliary Material and Supplementary Methods. (**D**) CG MD simulations of wild-type and loops-mutant C-terminal homodimers. (**E**) Distance from Phe^177^ or Asp^177^ to the nearest phospholipid phosphate in five representative 3-μs simulations. (**F**) Mean traces across all 10 simulations (wild-type, cyan; mutant, red) show stable low distances for wild-type and higher fluctuating distances for the mutant. (**G** and **H**) Full-length homodimer tethering between OMM and IMM bilayers. (G) Representative 5-μs trajectory showing stable dual-membrane engagement. (H) Backbone RMSD and distribution for wild-type (cyan) and mutant (red); the mutant shows higher, more variable RMSD, indicating reduced tether stability. (**I**) 3D reconstruction of a SPRIZ-like OMM-IMM bridge from cryo-ET data published in (*41*).

To complement the static membrane-positioning and protein-lipid interaction models from PPM and AlphaFold3, we next turned to CG MD as a physics-based dynamical calculation of membrane engagement by the SPRIZ C-terminal conical-domain homodimer. This approach integrates a defined interaction potential to evolve the protein-bilayer system over time, allowing the conical-domain homodimer to diffuse, collide with, and associate with (or fail to associate with) an IMM-like bilayer without imposed docking or restraints. We performed 10 independent 3-μs runs starting from the AlphaFold-predicted structure of the isolated SPRIZ C-terminal conical-domain homodimer.

Across replicates, the wild-type C-terminal homodimer underwent unbiased diffusion and stochastic encounters with the bilayer; when loops 1 and 2 from one monomer contacted the phospholipid headgroup region in a productive orientation, the interaction was captured and the C-terminal conical domain proceeded to graft and remain persistently membrane-associated for the remainder of the trajectory (Fig. 3D, top, and movie S4). In contrast, a loops mutant in which the hydrophobic residues in loops 1 and 2 were replaced by aspartates (Fig. 3B) showed impaired capture and stabilization, with the C-terminal conical domain failing to achieve stable bilayer grafting (Fig. 3D, bottom, and movie S5). Inspection of individual trajectories suggested a sequential engagement mechanism: One monomer’s loops typically graft first, followed by engagement of the second monomer, which stabilizes the persistent bilayer-associated state (movie S4). Quantification of membrane proximity using the residue-177–to-phosphate distance metric showed that wild-type trajectories converged to a sustained low-distance regime, whereas the loops mutant remained at higher, fluctuating distances and did not reach a comparably stable state (Fig. 3E). This distinction was preserved when all 10 simulations were aggregated, confirming a robust separation between wild type and the loops mutant in their average behavior (Fig. 3F).

To extend these insights to the full-length SPRIZ homodimer in an OMM-IMM tethering context, we performed three independent CG MD simulations (5 μs each) of the OMM-anchored SPRIZ homodimer positioned opposite an IMM-like bilayer (Fig. 3G and movie S6). Across trajectories, the C-terminal conical domain established stable IMM engagement and maintained a persistent tethered configuration without sustained detachment or loss of the spanning geometry, consistent with the behavior of the isolated C-terminal domain. RMSD analysis relative to the starting structure remained low and stable for wild-type SPRIZ (cyan trace) throughout the simulations (Fig. 3H, left), whereas the loops mutant (red trace) showed higher and more variable RMSD values, consistent with reduced stability of the tethered state. Correspondingly, RMSD distributions clustered below ∼6 Å for wild type, while the mutant exhibited a broader distribution peaking around ∼11 Å with minimal overlap (Fig. 3H, right), supporting that loop mutations compromise stable SPRIZ-mediated OMM-IMM tethering.

To directly visualize OMM-IMM–spanning structures in situ, we reanalyzed a published high-quality cryo-ET of mitochondria in focused ion beam (FIB)–milled mouse embryonic fibroblast (MEF) cells (*41*). Within the IMS, we identified electron-dense filamentous densities that appeared to bridge the OMM and IMM (fig. S9). By following individual densities through consecutive virtual sections spaced 0.6 nm apart, we established that one such structure remained continuous across approximately seven sections, corresponding to a reconstructed z-thickness of ∼4.2 nm. This dimension is consistent with the AlphaFold3-predicted architecture of the SPRIZ homodimer, which comprises an IMM-associated base of ∼4.5 nm and a coiled-coil rod of ∼2.3-nm diameter. Three-dimensional (3D) isosurface reconstruction of the tomographic volume revealed a characteristic rod-like morphology, with a wider base associated with the IMM and a narrower density extending across the IMS to contact the OMM (Fig. 3I and fig. S9). These observations provide in situ evidence for the existence of OMM-IMM–bridging rod-like densities whose architecture is compatible with the predicted SPRIZ tether.

Together, these multiscale simulations support a model in which the SPRIZ homodimer’s rigid coiled-coil rod positions a C-terminal conical domain that autonomously grafts into an IMM-like bilayer via loops 1 and 2, thereby enabling stable OMM-IMM tethering. This coupled structural-mechanistic framework motivated engineering a gain-of-function, lengthened SPRIZ variant to calibrate and causally probe SPRIZ-dependent IMS phenotypes.

### A length-extended SPRIZ gain-of-function tool (MARTINI) for in vivo IMS-geometry studies

To causally test whether SPRIZ functions as a spatial regulator of the IMS, we sought a gain-of-function variant whose effects on IMS expansion would scale proportionally with the length of SPRIZ’s rigid, near-perpendicular OMM-anchored rod-like structure. To this end, we engineered MARTINI (mitochondrial artificial increaser of the IMS) which selectively lengthens the coiled-coil rod while preserving the OMM anchor, homodimerization interface, and the full C-terminal conical, IMM-engaging module.

MARTINI incorporates a targeted in-frame, in-register duplication of the SPRIZ coiled-coil segment (Ala^58^-Arg^132^), extending the IMS-spanning region without disrupting coiled-coil register or overall folding (Fig. 4A). AlphaFold3 modeling predicted a SPRIZ-like MARTINI homodimer with high confidence, including an elongated coiled-coil shaft and preservation of the C-terminal dimer interface; consistent with this, the predicted aligned error map indicates strong confidence within and between the paired helices across the extended rod (fig. S10A). The model further predicts a substantial increase in end-to-end length (∼29 nm versus ∼17 nm for native SPRIZ; Fig. 4B). Experimentally, MARTINI–hemagglutinin (HA) localized to mitochondria in transfected HeLa cells with colocalization to TOM20 (Fig. 4C), and protease-protection assays on isolated mouse liver mitochondria expressing MARTINI confirmed correct transmembrane insertion and IMS-oriented C-terminal exposure (Fig. 4F). MARTINI retained self-association, as shown by homodimer coimmunoprecipitation (Fig. 4D) and split-superfolder GFP complementation (Fig. 4E). Last, all-atom MD simulations of OMM-anchored MARTINI in an OMM-like bilayer showed sustained structural integrity over 400 ns, with the coiled-coil remaining near-linear and near-perpendicular to the membrane plane (median interior angle ∼157.7°; Fig. 4, G and H, and movie S7) and low conformational drift (fig. S10, B and C). The C-terminal module retained a constrained flexibility comparable to wild-type SPRIZ: the median bend-angle distribution centered at ∼157.4° for MARTINI (fig. S10D) versus ∼131.6° for SPRIZ (fig. S8B), supporting preservation of the distal module’s hinge-like behavior despite rod lengthening. Collectively, these validations establish MARTINI as a properly folded, mitochondria-targeted, self-associating, rigid, length-extended SPRIZ variant that preserves both the near-perpendicular rod-like *z*-axis architecture and the calibrated flexibility of the C-terminal conical module needed for matched gain-of-function tests of rod length–dependent IMS phenotypes.

**Fig. 4.**
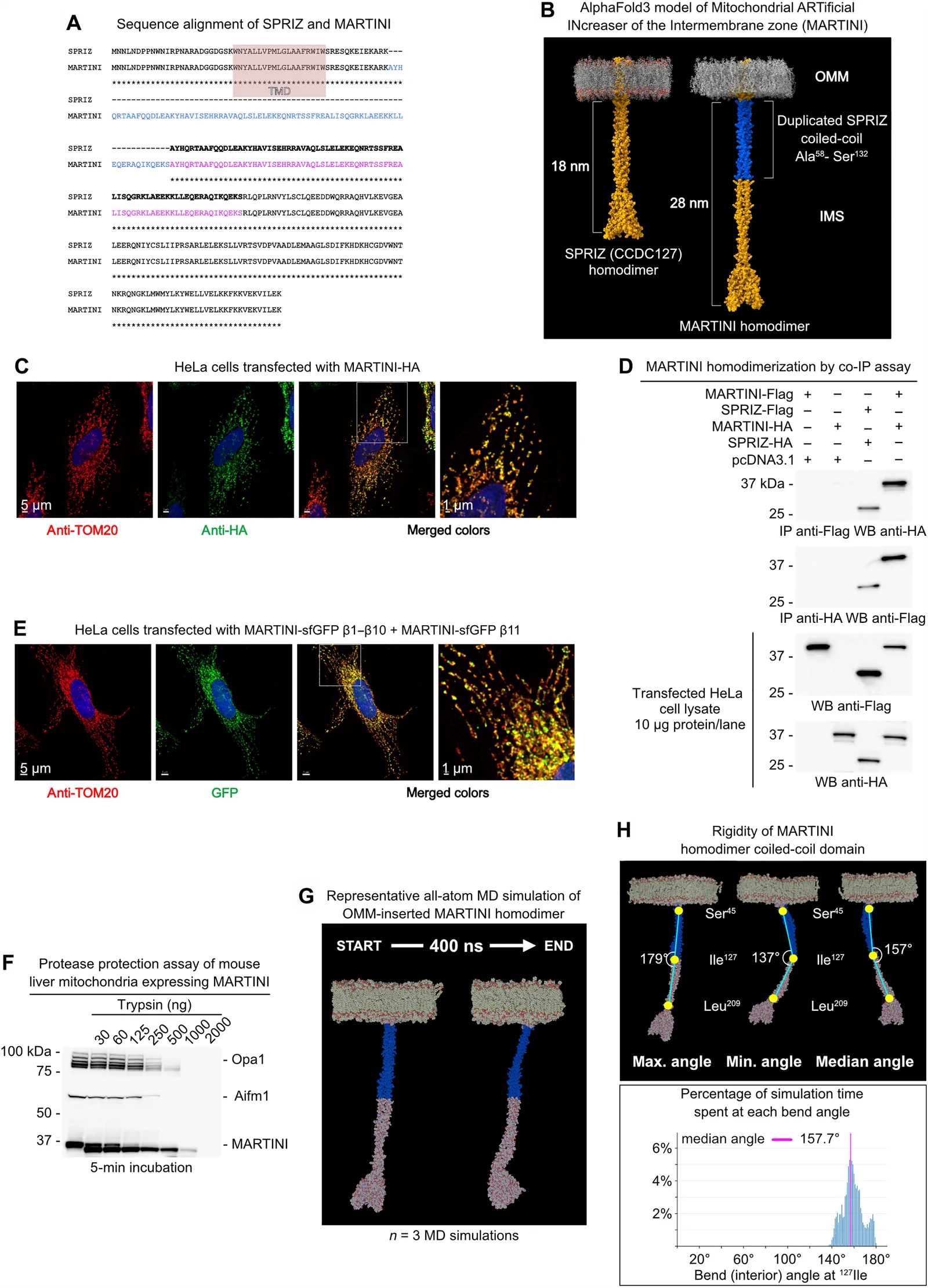
Rational design and structural/mechanical characterization of MARTINI, an engineered lengthened SPRIZ variant for controlled IMS expansion. (**A**) Sequence alignment of SPRIZ (CCDC127) and MARTINI. MARTINI retains the N-terminal transmembrane domain (TMD, boxed) and C-terminal conical domain but contains an in-frame, in-register duplication of the coiled-coil segment (Ala^58^-Arg^132^, blue), extending the IMS-spanning rod while preserving helical register. (**B**) AlphaFold3 models of SPRIZ and MARTINI homodimers. The duplicated MARTINI segment is blue. Predicted length increases from ∼18 to ∼28 nm, consistent with a gain-of-function tether designed to expand the IMS. (**C**) MARTINI mitochondrial localization in HeLa cells. MARTINI-HA (anti-HA, green) colocalizes with TOM20 (red); nuclei are stained with DAPI (blue). Right: enlarged boxed region. Scale bars, 5 μm (main) and 1 μm (zoom). (**D**) MARTINI homodimerization by coimmunoprecipitation. HeLa cells coexpressing MARTINI-Flag and MARTINI-HA, or SPRIZ controls, show reciprocal pulldown. Input lysates (10 μg per lane) confirm expression and molecular weight. (**E**) MARTINI self-association by split-superfolder GFP complementation. MARTINI-sfGFP(β1–10)–HA and MARTINI-sfGFP(β11)x7-Flag reconstitute GFP fluorescence (green) colocalized with TOM20 (red). Right: enlarged boxed region. Scale bars, 5 μm (main) and 1 μm (zoom). (**F**) Protease-protection assay confirming MARTINI topology in mouse liver mitochondria. Mitochondria from MARTINI-expressing livers were treated with trypsin (30 to 2000 ng, 5 min). MARTINI is protected at low concentrations, like IMS/IMM markers Opa1 and Aifm1, and progressively lost at higher doses, consistent with an N-cytosolic, IMS-facing orientation. (**G** and **H**) All-atom MD simulations of OMM-anchored MARTINI homodimer. (G) Representative trajectory showing stable transmembrane insertion and upright coiled-coil conformation at the start and after 400 ns. (H) Coiled-coil rigidity quantified using bend angles defined by Ser^45^, Ile^127^, and Leu^209^ (yellow markers). The Ile^127^ angle distribution has a median of ∼158°, indicating a near-linear coiled-coil with limited bending.

### Attenuation of mitochondrial Ca^2+^ uptake by SPRIZ (CCDC127) and MARTINI in transfected HeLa cells

We exploited the rigid, OMM-anchored, near-perpendicular rod-like architecture of SPRIZ and MARTINI—which spans the IMS—to test whether their overexpression in cultured HeLa cells expands the IMS, with MARTINI eliciting a proportionally greater effect. As an initial structural check, EM sections showed largely preserved overall mitochondrial morphology across conditions; at the same time, higher-magnification views of the mitochondrial periphery revealed increased apparent OMM-IMM separation in cells expressing SPRIZ, Xenopus SPRIZ, or MARTINI relative to controls, consistent with IMS expansion (Fig. 5A).

**Fig. 5.**
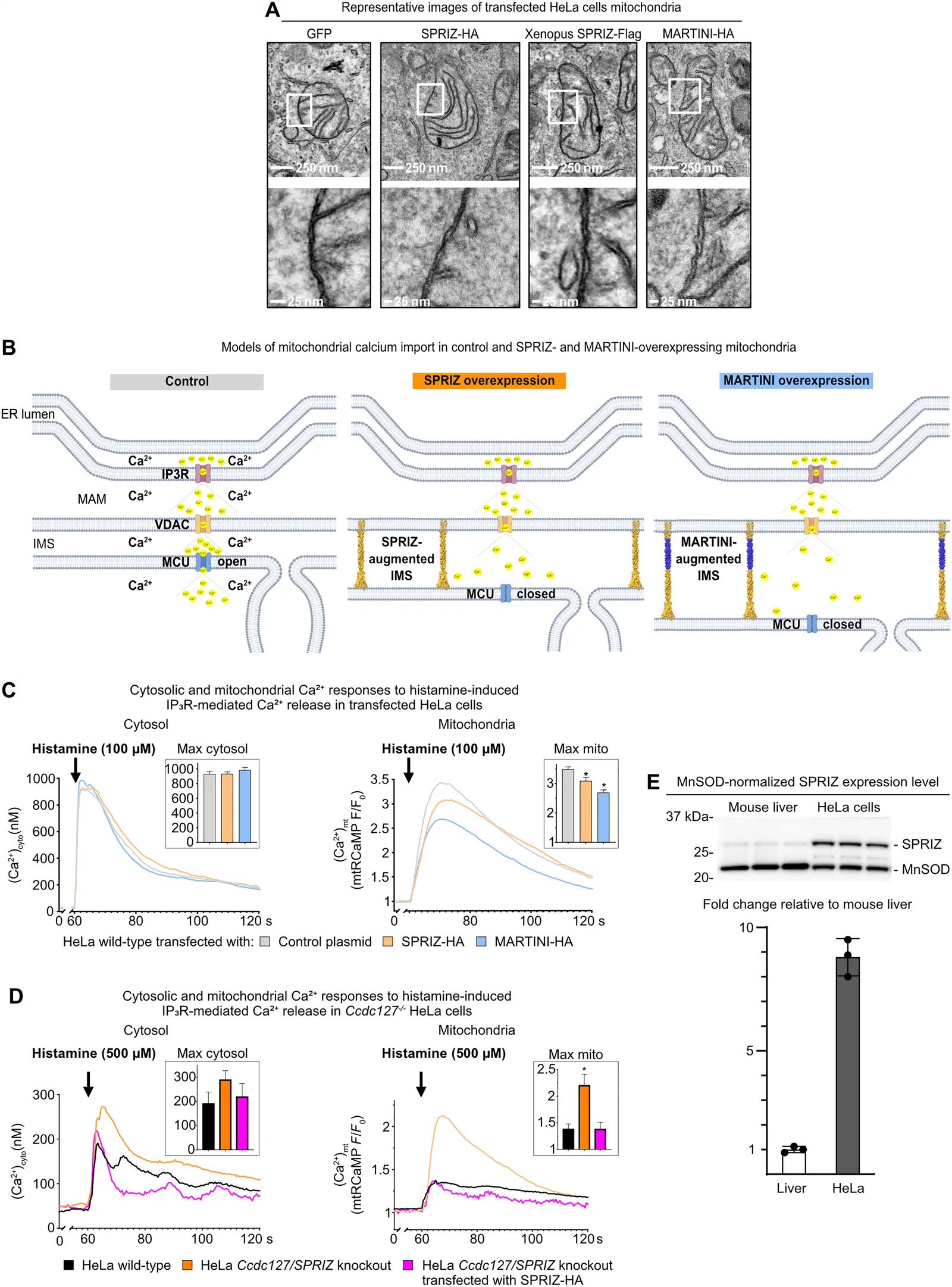
SPRIZ and MARTINI overexpression impairs mitochondrial Ca^2+^ uptake in transfected HeLa cells. (**A**) Representative transmission electron microscopy (TEM) images of mitochondrial ultrastructure in control HeLa cells or cells expressing SPRIZ-HA, Xenopus SPRIZ-Flag, or MARTINI-HA. Top rows: low-magnification views; bottom rows: high-magnification views of boxed regions showing OMM-IMM spacing and cristae organization. (**B**) Schematic model of Ca^2+^ transfer at mitochondria-associated membranes (MAMs). In control cells, IP_3_R-mediated ER Ca^2+^ release generates a local microdomain that is efficiently relayed via VDAC to MCU. SPRIZ- or MARTINI-mediated IMS expansion disperses Ca^2+^ within the IMS, weakening VDAC-MCU coupling and thereby slowing the rate and reducing the efficiency of mitochondrial Ca^2+^ uptake. Created in BioRender. Harper, M. (2026) https://BioRender.com/0872y2w. (**C**) Cytosolic (left) and mitochondrial (right) Ca^2+^ responses recorded simultaneously to histamine (100 μM)–induced IP_3_R-mediated ER Ca^2+^ release. Mean traces for control (gray), SPRIZ (orange), and MARTINI (blue). Insets show peak responses (means ± SEM). Mitochondrial Ca^2+^ uptake is significantly reduced in SPRIZ- and MARTINI-expressing cells (*n* = 3 independent biological replicates; >50 cells quantified per replicate; *P < 0.05 by one-way analysis of variance (ANOVA) with Tukey’s multiple-comparisons test). (**D**) Cytosolic (left) and mitochondrial (right) Ca^2+^ responses recorded simultaneously to histamine (500 μM)–induced IP_3_R-mediated ER Ca^2+^ release. Mean traces for control (black), SPRIZ KO (dark orange), and SPRIZ KO + SPRIZ-HA (magenta) cells. Insets show peak responses (means ± SEM). (*n* = 3 biological replicates; mean traces of ≥15 cells quantified per replicate; *P < 0.05 by one-way ANOVA with Tukey’s multiple-comparisons test). (**E**) Representative immunoblot showing endogenous SPRIZ levels in mouse liver mitochondria–enriched fractions and HeLa cells. MnSOD was used as a mitochondrial loading control. Quantification of MnSOD-normalized SPRIZ signal is shown on the right as fold change relative to mouse liver, with individual dots representing independent samples and bars indicating means ± SEM.

Given this apparent IMS-selective geometric change, we next sought functional validation of the central model by measuring the mitochondrial matrix Ca^2+^ signal, which requires efficient Ca^2+^ transfer across the IMS from OMM entry pores (VDAC) to the IMM Ca^2+^ uniporter complex (MCU). Increasing IMS volume is expected to lengthen the aqueous diffusion path between VDAC and MCU and dilute local Ca^2+^ at the uniporter, thereby increasing the Ca^2+^ load required to reach MCU activation thresholds and reducing uptake efficiency (Fig. 5B). Consistent with this model, mitochondrial matrix Ca^2+^ rise was selectively reduced upon SPRIZ or MARTINI expression despite preserved cytosolic Ca^2+^ transients. Specifically, after histamine-evoked IP_3_R-mediated ER Ca^2+^ release, cytosolic Ca^2+^ signals were comparable across conditions, whereas the mitochondrial Ca^2+^ response (mtRCaMP) was diminished in terms of rising phase and peak in SPRIZ- and MARTINI-expressing cells, with MARTINI producing the stronger effect (Fig. 5C). These effects were not explained by changes in the uniporter machinery, as MICU1, MCU, and VDAC levels were comparable in control, SPRIZ-HA, and MARTINI-HA lysates (fig. S11, B to D).

To validate these findings, we tested whether the attenuated mitochondrial Ca^2+^ uptake could instead reflect altered ER–mitochondria contact architecture, which could reduce Ca^2+^ delivery from IP_3_R-containing ER domains to mitochondria independently of IMS expansion. To address this, we performed quantitative EM analysis of mitochondria-associated membranes in the same transfected HeLa cell system. SPRIZ expression did not significantly alter the number of MAM contacts per cell, nor did it change the fraction of mitochondria displaying 0, 1, or ≥2 MAM contacts (fig. S11E; *n* = 20 cells per group; >550 mitochondria per group). In addition, individual MAM contact length and the percentage of mitochondrial perimeter occupied by MAM contacts were unchanged relative to GFP-expressing controls (fig. S11F). Thus, the reduced mitochondrial matrix Ca^2+^ response in SPRIZ-expressing cells is not explained by gross remodeling of ER-mitochondria contacts, supporting the interpretation that impaired Ca^2+^ transfer reflects altered OMM-IMM coupling across the expanded IMS.

To test the role of endogenous SPRIZ in Ca^2+^ transfer across the IMS, we recorded histamine-induced Ca^2+^ transients in wild-type and *Ccdc127/SPRIZ* knockout HeLa cells. Cytosolic Ca^2+^ transients were comparable across genotypes; however, the mitochondrial matrix Ca^2+^ response was significantly enhanced in the *Ccdc127/SPRIZ* knockout cells (Fig. 5D). The specific involvement of SPRIZ in this process was validated by genetic rescue in the *Ccdc127/SPRIZ* knockout cells, which reverted the mitochondrial Ca^2+^ rise to the level of the wild-type cells (Fig. 5D). Together with the overexpression studies, these loss of function and rescue experiments converge to support a role for SPRIZ in the control of Ca^2+^ transfer across the IMS. Together, these cell-based structural and functional readouts are consistent with SPRIZ-dependent IMS expansion and motivated direct in vivo tests of SPRIZ and MARTINI as modulators of IMS geometry in mouse liver mitochondria.

### AAV8-mediated hepatic expression of SPRIZ and MARTINI drives graded IMS expansion in mouse liver mitochondria

Building on the cell-based functional readouts consistent with SPRIZ-dependent IMS expansion, we next tested whether elevating SPRIZ levels is sufficient to remodel mitochondrial ultrastructure in vivo. To enable a causal structure-function comparison, we expressed SPRIZ alongside its lengthened variant MARTINI, allowing us to ask whether any IMS phenotype is accentuated when the encoded rod length is increased. We generated AAV8 vectors expressing either protein under the liver-specific thyroxine-binding globulin (TBG) promoter (Fig. 6A) (*21*) and injected them into 6-month-old male mice. Immunoblotting of liver mitochondria–enriched fractions confirmed expression 3 weeks after injection (Fig. 6A, right), which we selected for subsequent morphometric and functional analyses. At the tissue level, liver appearance was indistinguishable from controls. At the cellular level, EM sections showed broadly preserved hepatocyte organization, including an apparently intact wrappER network surrounding mitochondria and no obvious disruption of other major membrane systems.

**Fig. 6.**
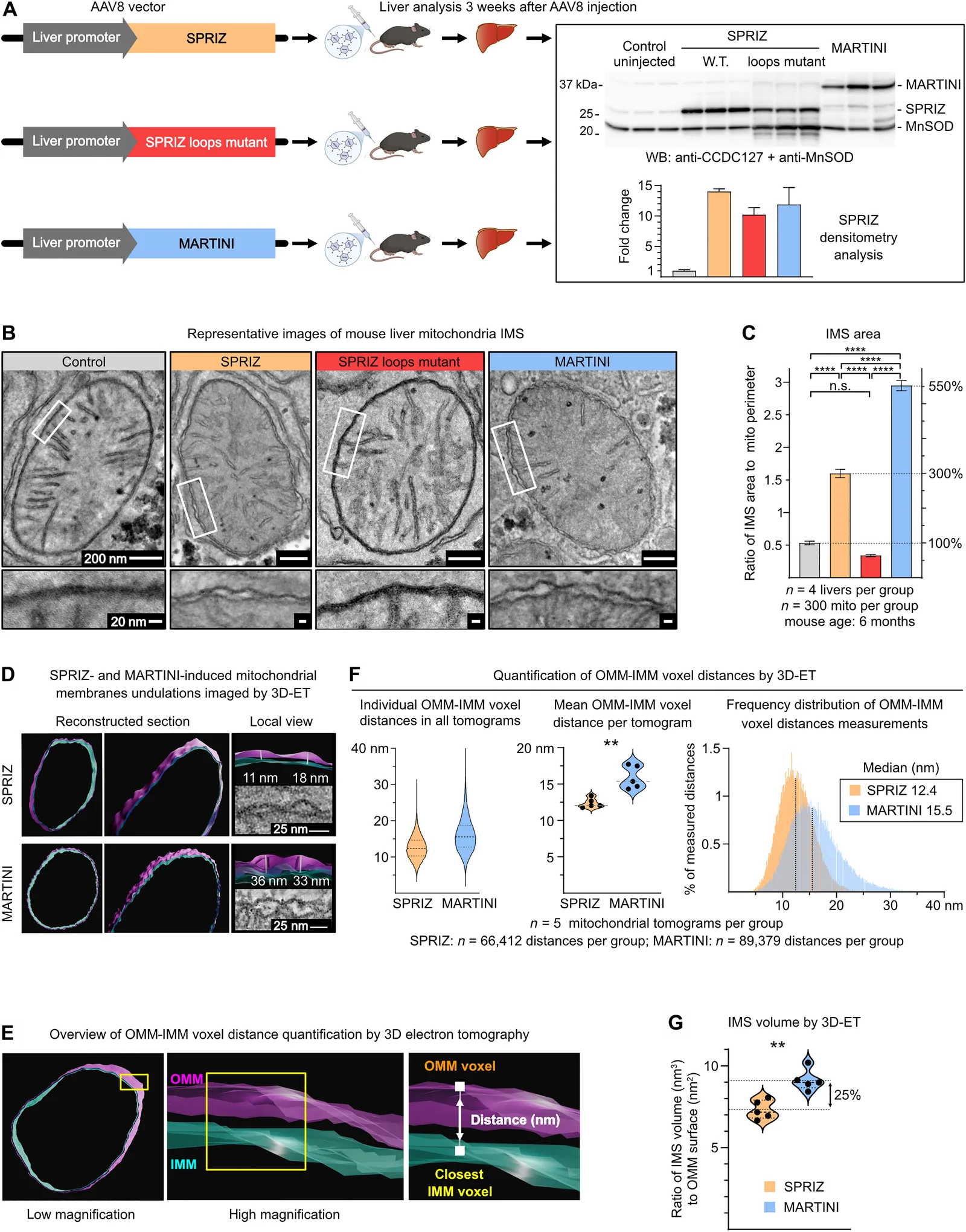
SPRIZ and MARTINI drive length-dependent IMS expansion in mouse liver mitochondria. (**A**) Experimental design. Six-month-old male mice received liver-specific AAV8-TBG vectors expressing SPRIZ, SPRIZ loops mutant (176LFL → 176DDD; 222VW → 222DD), or MARTINI. Livers were collected 3 weeks later for EM morphometry and biochemical assays. Immunoblotting of mitochondria-enriched fractions confirms expression; MARTINI migrates at ∼37 kDa and SPRIZ constructs at ∼30.5 kDa. MnSOD is the loading control. Bottom right: expression densitometry. Created in BioRender. Harper, M. (2026) https://BioRender.com/xy0xdz0. (**B**) Representative TEM images from control, SPRIZ-, loops mutant-, and MARTINI-expressing livers. Boxed expanded-IMS regions are enlarged below. (**C**) IMS area/mitochondrial perimeter. Workflow shown in fig. S6E; all individual data points in fig. S12E. *n* = 4 livers per group; 300 mitochondria per group. ****P < 0.0001 by one-way ANOVA with Tukey’s multiple-comparisons test; means ± SEM. (**D**) SPRIZ- and MARTINI-induced membrane undulations analyzed by 3D-ET. Reconstructed sections, enlarged undulations, and local undulations with corresponding tomographic slices are shown from left to right. OMM and IMM are magenta and cyan; local distances are indicated. Scale bars, 25 nm. (**E**) OMM-IMM voxel-distance workflow. Each OMM voxel is assigned the distance to the nearest IMM voxel. (**F**) OMM-IMM voxel distances by 3D-ET. Violin plots show distributions of individual OMM-IMM voxel distances with medians and quartiles (left) and mean OMM-IMM voxel distance per tomogram (middle; *n* = 5 mitochondrial tomograms per group; **P < 0.01 by Mann–Whitney *U* test). Frequency plots show individual distance measurements and group medians. SPRIZ median, 12.4 nm; MARTINI median, 15.5 nm. (**G**) IMS volume/OMM surface area by 3D-ET. Dots represent individual tomograms; dotted lines indicate group means [*n* = 5 mitochondrial tomograms per group; **P < 0.01 by Mann-Whitney *U* test; SPRIZ: 95% confidence interval (CI), 6.598 to 8.035; MARTINI: 95% CI, 8.312 to 9.931].

Quantitative EM morphometry nonetheless revealed a shared shift in global mitochondrial metrics under both SPRIZ and MARTINI: mitochondrial number per field decreased by ∼30% while average mitochondrial cross-sectional area increased by ∼15 to 20% relative to controls (fig. S12, C and D; >10,000 mitochondria quantified per group). We next quantified cristae parameters. Although inspection of individual sections did not reveal a consistent or overt *cristae* abnormality, morphometry detected divergent remodeling by the two constructs: The fraction of mitochondrial area occupied by cristae increased with SPRIZ (from ∼0.036 in controls to ∼0.045) but decreased with MARTINI (to ∼0.021), and cristae width shifted accordingly (SPRIZ, 25 → 27 nm; MARTINI, 25 → 20 nm) (fig. S12, A and B; *n* ≥ 170 mitochondria per group).

In contrast to these relatively subtle cristae changes, the mitochondrial periphery showed an immediately distinctive phenotype in SPRIZ- and especially MARTINI-expressing mitochondria (Fig. 6B and fig. S12F). High-magnification views revealed a visibly widened OMM-IMM separation compared with controls, which typically displayed smooth, closely apposed, near-parallel membrane profiles (Fig. 6B and fig. S12F). Under SPRIZ expression, the IMS was visibly widened and the peripheral OMM/IMM profiles became undulated, with waviness that was further amplified and extended along longer stretches of the perimeter in MARTINI-expressing mitochondria. Under MARTINI expression, the apparent IMS widening was more pronounced and was frequently accompanied by extended regions of undulated (“wavy”) OMM/IMM profiles along the perimeter (Fig. 6, B and D, and fig. S12, F to H).

Quantitative morphometry confirmed that this visually prominent phenotype reflects a large, graded expansion of the IMS. Using the IMS-area workflow described in fig. S6E, we measured 300 mitochondria per group (*n* = 4 livers per group) and expressed IMS area normalized to mitochondrial perimeter. Relative to controls, SPRIZ increased the IMS-area/perimeter ratio by ∼3-fold (from ∼0.55 to ∼1.6), whereas MARTINI drove an even larger ∼5.5-fold increase (to ∼2.9) (Fig. 6C and fig. S12E). All pairwise comparisons were highly significant (*P* < 0.00001). Thus, SPRIZ expression is sufficient to expand the IMS in vivo, and MARTINI drives a proportionally larger expansion consistent with its increased rod length.

### SPRIZ-dependent IMS expansion requires the predicted IMM-engaging loops

The robust IMS expansion produced by hepatic SPRIZ expression provided an opportunity to test two connected predictions of our structural model: first, that loop-1 and loop-2 within the C-terminal conical domain are functional determinants of IMM engagement and, second, that SPRIZ-dependent IMS expansion requires this predicted tethering interface. To address this, we generated an SPRIZ loops mutant in which the predicted IMM-interacting residues identified in Fig. 3, A to H were disrupted and expressed it in mouse liver using the same AAV8-TBG strategy used for wild-type SPRIZ and MARTINI (Fig. 6A). Immunoblotting of liver mitochondria–enriched fractions confirmed robust recovery of the loops mutant 3 weeks after injection, with MnSOD-normalized expression comparable to wild-type SPRIZ (Fig. 6A, right). Complementary biochemical and structural analyses further established that the loops mutant retains the correct OMM-anchored, IMS-exposed topology, forms the expected ∼61-kDa homodimer with efficiency comparable to wild-type SPRIZ, and remains structurally stable (fig. S13, A to I).

In representative EM sections, mitochondria expressing the loops mutant did not show the widened IMS or undulated OMM/IMM profiles characteristic of wild-type SPRIZ expression. Instead, their peripheral membrane organization resembled control liver mitochondria, with closely apposed OMM and IMM profiles along the mitochondrial perimeter (Fig. 6B and fig. S12F). Quantitative morphometry confirmed this visual impression. Whereas wild-type SPRIZ increased the IMS-area/perimeter ratio by ∼threefold and MARTINI produced an even larger increase, the SPRIZ loops mutant failed to increase IMS area and was indistinguishable from control mitochondria (Fig. 6C). Thus, this loss of phenotype cannot be explained by lack of expression, altered topology, failure of dimerization, or structural destabilization. Rather, it shows that disruption of the predicted IMM-engaging loops abolishes SPRIZ-dependent IMS expansion in vivo. These data therefore experimentally validate the loop-dependent tethering prediction from Fig. 3 and identify loop-1 and loop-2 as required structural determinants of SPRIZ-mediated IMS remodeling in mouse liver mitochondria.

### Electron tomography reveals SPRIZ- and MARTINI-dependent expansion of the IMS volume

To extend our ultrastructural analysis from two to three dimensions, we performed electron tomography on mitochondria in the liver of mice expressing SPRIZ or MARTINI. Five independent mitochondrial tomograms were reconstructed and segmented for each condition, allowing visualization of the OMM (magenta) and inner boundary membrane/IMM (teal) in full 3D context (Fig. 6D and fig. S12, G and H). These reconstructions confirmed the presence of membrane undulations in both conditions and revealed qualitatively larger local OMM-IMM separations in MARTINI-expressing mitochondria. To quantify OMM-IMM spacing directly in 3D, we implemented a voxel-based workflow in which the shortest 3D distance from every OMM voxel to its nearest IMM voxel was measured across the entire boundary membrane (Fig. 6E and fig. S12I). This approach generated comprehensive distance distributions from >66,000 (SPRIZ) and >89,000 (MARTINI) individual voxel measurements. The resulting distributions demonstrated a clear rightward shift for MARTINI, with median OMM-IMM distances of 12.4 nm in SPRIZ-expressing mitochondria versus 15.5 nm in MARTINI-expressing mitochondria (Fig. 6F). Using the same 3D reconstructions, we additionally calculated IMS volume normalized to OMM surface area. This true volumetric measurement showed that MARTINI increases the IMS volume/OMM surface ratio to approximately 125% of the SPRIZ value (Fig. 6G). Together, these electron tomography analyses establish that the longer engineered MARTINI tether produces a measurable increase in OMM-IMM separation and IMS volume relative to SPRIZ when quantified directly in 3D reconstructed mitochondrial volumes.

### SPRIZ and MARTINI generate functional increases in intermembrane volume in mouse liver mitochondria

To functionally validate the SPRIZ- and MARTINI-driven IMS expansion observed by EM, we next turned to mitochondria-enriched liver fractions and applied orthogonal assays expected to be sensitive to intermembrane volume and OMM-IMM coupling/apposition. First, we adapted a radiotracer assay of mitochondrial compartment accessibility (Fig. 7A). Freshly isolated liver mitochondria–enriched fractions from control mice and mice expressing SPRIZ, the SPRIZ loops mutant, or MARTINI were incubated iso-osmotically with ^14^C-mannitol or ^14^C-dextran. Mannitol crosses the OMM through VDAC to access the IMS but is excluded from the matrix by the IMM, whereas high–molecular weight dextran is excluded by VDAC and controls for nonspecific carryover, surface retention, and pelleting artifacts. Consistent with EM morphometry (Fig. 6, B and C), mitochondria expressing SPRIZ or MARTINI retained more ^14^C-mannitol than controls (Fig. 7B). The SPRIZ loops mutant failed to increase ^14^C-mannitol accessibility and resembled controls (Fig. 7B). Low and unchanged ^14^C-dextran retention across conditions confirmed that elevated ^14^C-mannitol signals reflect specific IMS accessibility rather than nonspecific radiotracer retention, membrane damage, or incomplete washing.

**Fig. 7.**
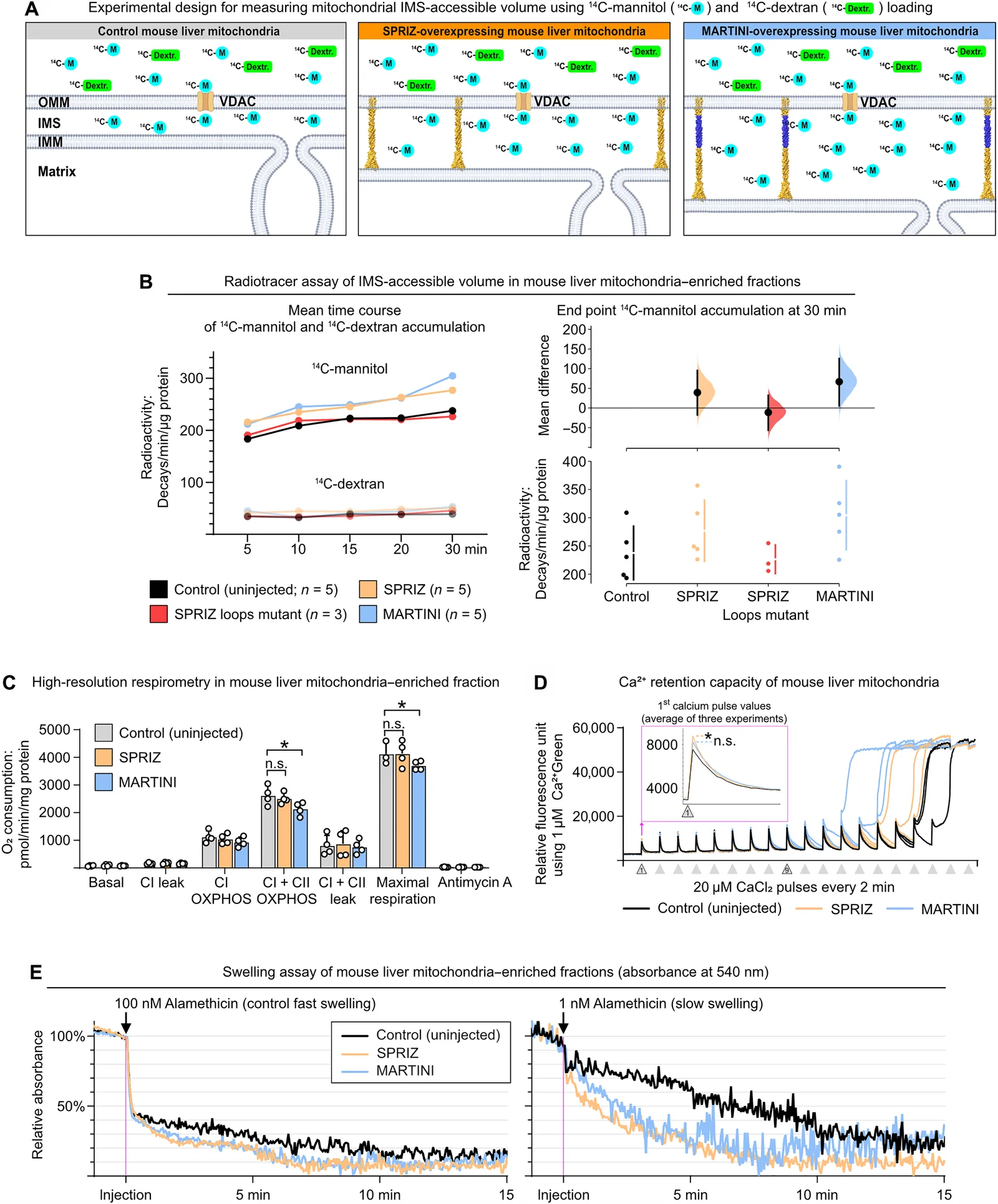
Functional readouts of increased IMS in SPRIZ- and MARTINI-expressing mitochondria. (**A**) Experimental design. ^14^C-mannitol enters the IMS through VDAC, whereas ^14^C-dextran remains extramitochondrial and controls for carryover. SPRIZ- or MARTINI-mediated IMS expansion increases the mannitol-accessible compartment. Created in BioRender. Harper, M. (2026) https://BioRender.com/0872y2w. (**B**) Radiotracer measurements in mouse liver mitochondria-enriched fractions from uninjected control, SPRIZ-, SPRIZ loops mutant, and MARTINI-expressing mice. Left: mean time courses of ^14^C-mannitol and ^14^C-dextran accumulation normalized to protein. Right: 30-min 14C-mannitol accumulation shown as a Gardner-Altman estimation plot. Individual values are shown below and bootstrap distributions of mean differences versus control above. Black dots indicate mean differences; vertical lines indicate 95% confidence intervals. Data are shown for control (black; *n* = 5), SPRIZ (orange; *n* = 5), SPRIZ loops mutant (red; *n* = 3), and MARTINI (blue; *n* = 5). (**C**) High-resolution respirometry in liver mitochondria-enriched fractions. Oxygen consumption rates (picomole of O_2_ per minute per milligrams of protein) under the indicated respiratory states are shown as individual biological replicates with group means ± SD. *P < 0.05, n.s., not significant by two-way ANOVA with Tukey’s multiple-comparisons test. (**D**) Ca^2+^ retention capacity in mouse liver mitochondria–enriched fractions. A total of 20 μM CaCl_2_ pulses (gray arrowheads) were added every 2 min while monitoring extramitochondrial Ca^2+^ with 1 μM Ca^2+^ Green. Traces show three independent biological replicates per group for control (black), SPRIZ (orange), and MARTINI (blue). Inset (magenta box) quantifies the first Ca^2+^ pulse (mean of three experiments); statistics by two-way ANOVA with Tukey’s multiple-comparisons test (*P < 0.05; n.s., not significant). (**E**) Alamethicin-induced swelling in liver mitochondria–enriched fractions, monitored as decreased absorbance at 540 nm. Representative traces show control (black), SPRIZ (orange), and MARTINI (blue). Left: 100 nM alamethicin induces rapid, comparable swelling. Right: 1 nM alamethicin reveals graded acceleration in SPRIZ and MARTINI samples. Arrow indicates alamethicin addition.

We next measured calcium retention capacity (CRC) in isolated liver mitochondria (Fig. 7D; *n* = 3 independent experiments). In this assay, mitochondria receive repeated Ca^2+^ pulses while extramitochondrial Ca^2+^ is monitored fluorometrically. The first Ca^2+^ pulse reached a significantly higher extramitochondrial Ca^2+^ peak in SPRIZ mitochondria compared with control (inset in Fig. 7D), which might reflect slower access of Ca^2+^ to the mitochondrial calcium uniporter complex mtCU owing to the expanded IMS. MARTINI showed a similar but nonsignificant trend. With repetitive pulsing, both SPRIZ- and MARTINI-expressing mitochondria were prone to delayed Ca^2+^ dysregulation (likely involving sensitization of the permeability transition pore), reaching the retention limit earlier than controls. This graded reduction in CRC tracks with the graded IMS expansion driven by SPRIZ versus MARTINI.

Because the SPRIZ loops mutant is expressed but fails to expand the IMS, it provided a negative structure-function comparator to ask whether the Ca^2+^-handling phenotype follows IMS expansion. In CRC assays performed on liver mitochondria–enriched fractions, wild-type SPRIZ reproduced the expected shift in Ca^2+^ handling, including the higher first-pulse extramitochondrial Ca^2+^ peak, whereas the SPRIZ loops mutant behaved similarly to control uninjected mitochondria (fig. S14A). Thus, the Ca^2+^-handling phenotype segregates with SPRIZ-dependent IMS expansion and is lost in a mutant that fails to support loop-dependent OMM-IMM tethering.

We then used alamethicin-induced swelling as a second volume-sensitive readout (Fig. 7E). Alamethicin forms nonselective pores in mitochondrial membranes, enabling osmotic water influx into the matrix that is detected as a decrease in A540 light scattering. When pore formation is limiting, effective intermembrane volume and OMM-IMM apposition are expected to influence how rapidly the osmotic perturbation equilibrates across the organelle, thereby modulating swelling kinetics. Consistent with this expectation, mitochondria from SPRIZ- and MARTINI-expressing livers swelled faster than controls at low-dose alamethicin (1 nM), whereas high-dose alamethicin (100 nM) produced rapid, comparable maximal swelling in all groups (Fig. 7E). This selective acceleration under limiting pore conditions provides an additional functional signature consistent with SPRIZ- and MARTINI-driven IMS enlargement affecting volume-dependent osmotic behavior. The loops mutant produced the same negative segregation in the swelling assay. At low-dose alamethicin, loops-mutant mitochondria failed to reproduce the SPRIZ-dependent swelling phenotype and instead tracked closely with control mitochondria (fig. S14B). Together with the CRC data, these results show that the functional phenotypes associated with SPRIZ expression segregate with IMS expansion and are lost in a mutant that is expressed but fails to support loop-dependent OMM-IMM tethering.

As a baseline functional control, high-resolution respirometry revealed no major changes in oxidative phosphorylation, and fatty-acid oxidation capacity was unchanged (Fig. 7C and fig. S15, A and B). Thus, although hepatic SPRIZ overexpression was accompanied by limited cristae remodeling (fig. S12, A and B), these structural changes did not translate into a detectable impairment of respiratory capacity under our in vitro assay conditions, in which isolated mitochondria are supplied with excess substrates and effectors and equilibration across the IMS can occur. Together with the IMS ultrastructural changes imposed by SPRIZ and MARTINI overexpression, these data provide functional evidence that SPRIZ acts as a spatial regulator of the IMS.

## DISCUSSION

In this study, we present converging evidence that the product of the *Ccdc127* gene, which we named SPRIZ, governs the geometry of the IMS. SPRIZ assembles as a homodimeric, OMM-anchored molecule whose rigid, near-perpendicular coiled-coil rod projects across the IMS and terminates in a C-terminal conical module capable of engaging the IMM (Fig. 3, G to I)—an architecture intrinsically suited to impose defined local membrane separation along the *z* axis and thereby generate discrete pockets of expanded intermembrane volume (Fig. 6, A to G).

The most decisive evidence that SPRIZ is sufficient to set IMS geometry comes from gain-of-function experiments in mouse liver: SPRIZ overexpression drove a large IMS expansion through the induction of widespread undulations of the mitochondrial envelope. These membrane deformations, which were accentuated by the engineered lengthened variant MARTINI, occurred throughout the organelle, including in regions devoid of cristae (Fig. 6D and fig. S12, G and H), and produced a graded, rod length–dependent increase in IMS area (∼300% for SPRIZ versus ∼550% for MARTINI; Fig. 6C). Because these undulations directly generate expanded IMS volume even in the absence of cristae, these results demonstrate that SPRIZ can increase IMS volume independently of cristae and their organizing machinery. Together, these findings support a model in which SPRIZ tunes IMS volume locally by constraining or expanding OMM-IMM separation at discrete sites. In the loss-of-function setting, this occurs without detectable changes in cristae organization or MICOS components, whereas gain-of-function SPRIZ expression can produce limited cristae remodeling without detectably impairing mitochondrial respiration. Whether these cristae changes reflect direct consequences of altered OMM-IMM spacing or pleiotropic effects of SPRIZ overexpression remains to be established.

Several observations establish that the primary role of SPRIZ is local regulation of IMS geometry and argue against its function as an integral or essential component of the MICOS complex or of CJ biology. The identification of SPRIZ in high–molecular weight complexes by BN-PAGE (Fig. 2G) remains compatible with a possible auxiliary contribution to the still incompletely understood processes of CJ assembly, maintenance, remodeling, or elimination at sites of proximity to the MICOS machinery. Separately, SPRIZ has an evolutionary origin and history distinct from those of known MICOS components and acquired vertebrate-specific structural innovations—the extended coiled-coil and IMM-grafting loops—that enable local control of IMS geometry. Most critically, cristae density and morphology remain fully preserved in the liver mitochondria of *Ccdc127^−/−^* mice (Fig. 1, K and L, and fig. S6, C, D, and F), and comparative proteomics of wrappER-associated mitochondria-enriched fractions from these animals demonstrate robust depletion of SPRIZ with no detectable changes in the mitochondrial proteome or in MICOS complex subunit expression (Fig. 1J, data file S2, and fig. S6A). Consistent with this, quantitative proteomics show that SPRIZ is present at substantially lower levels than the central MICOS scaffold Mic60 (∼eightfold; table S2), and super-resolution imaging indicates that endogenous SPRIZ puncta do not appreciably colocalize with Mic60 in HeLa cells (*24*). Together, these findings establish SPRIZ as a dedicated, vertebrate-specific regulator that tunes IMS volume locally and independently of the core MICOS scaffold, revealing that—at least in vertebrates—IMS geometry is a genetically specified and tunable structural parameter of mitochondria. At the same time, they leave open the intriguing possibility that SPRIZ, through its proximity to CJs, may have been secondarily co-opted in vertebrates to enhance the efficiency or robustness with which mitochondria handle CJ biology as a whole.

Additional support for the structural plausibility of this model comes from reanalysis of a published cryo-ET of FIB-milled MEF cells from Kucińska *et al.* (*41*). Although that study focused on nuclear-envelope remodeling and linker of nucleoskeleton and cytoskeleton complex filaments, our inspection of the original tomographic volume revealed electron-dense filamentous densities within the mitochondrial IMS that span the OMM-IMM interval (Fig. 3I and fig. S9). These densities extend approximately perpendicular to the two membranes and appear continuous with both the OMM and IMM. Because the tomogram consists of successive virtual z-slices through the reconstructed volume, spaced 0.6 nm apart, individual densities could be followed across consecutive sections. One such density is visible from section 36 through section 42 and the remaining filamentous throughout the *z* series and corresponding to an apparent *z* axis thickness of approximately 4.2 nm (fig. S9). This value is compatible with the AlphaFold3-based structural prediction of the SPRIZ homodimer tether, including an IMM-engaging base of approximately 4.5 nm and a coiled-coil rod diameter of approximately 2.3 nm. We do not infer molecular identity from the cryo-ET density alone, and the absence of a frequency comparison between wild-type and *Ccdc127-*knockout mitochondria remains a limitation of this analysis. However, these observations provide direct in situ visualization of rod-like OMM-IMM–spanning densities with the orientation and dimensions expected for the predicted SPRIZ homodimer tether.

The combined use of an in vivo mouse liver model and cultured HeLa cells provides complementary and mutually reinforcing evidence that SPRIZ regulates mitochondrial function primarily by modulating IMS geometry in a dose- and context-dependent manner. Several observations from the two systems support this view. In mouse liver, where endogenous SPRIZ abundance is relatively low (Fig. 5E and table S2), constitutive deletion of *Ccdc127* produced no significant changes in CRC or in low-dose alamethicin- or calcium-induced swelling (fig. S14, C to E). These functional readouts were therefore unaffected by the ∼35% increase in IMS area (Fig. 1M), which occurred without detectable alterations in cristae organization, MICOS components, or mitochondrial abundance (Fig. 1, J to L, and fig. S6, B to D). In contrast, hepatic overexpression of SPRIZ increased total SPRIZ protein levels ∼14-fold (∼7-fold increase in the homodimer) and expanded IMS area by ∼300%, whereas the lengthened MARTINI variant generated an even greater, graded expansion of ∼550% (Fig. 6C and fig. S12E). These IMS area increases were corroborated by radiotracer measurements, 3D EM reconstructions, elevated extramitochondrial Ca^2+^ after the first pulse, and accelerated low-dose alamethicin-induced swelling (Figs. 6, A to G, and 7, A to E). HeLa cells, which express nearly an order of magnitude more SPRIZ than liver (Fig. 5E and table S2), displayed clear bidirectional effects on mitochondrial Ca^2+^ uptake—enhanced by SPRIZ knockout and attenuated by overexpression of SPRIZ or MARTINI (Fig. 5, C and D, and fig. S11A), despite preserved cytosolic Ca^2+^ transients and unaltered abundance of the calcium uptake machinery (fig. S11, B to G). Together, these results support a dose-dependent influence of SPRIZ on IMS geometry rather than direct regulation of any single IMS-dependent process. Consistent with this interpretation, loop mutants designed to disrupt OMM-IMM tethering failed to expand the IMS (Fig. 6, B and C) and did not reproduce the IMS-related functional phenotypes (Fig. 7B and fig. S14, A and B). Notably, within this framework, calcium handling emerges as a functional readout of IMS geometry rather than as the exclusive biological output of SPRIZ activity, as further supported by the ^14^C-mannitol accumulation assay (Fig. 7, A and B). By changing the size and organization of the intermembrane compartment, SPRIZ is expected to influence any process that depends on diffusion, buffering, or local concentration gradients within the IMS, including metabolite exchange, ROS signaling, protein import, lipid transfer, and osmotic equilibration. In this way, SPRIZ may provide vertebrates with a mechanism to locally and dynamically tune mitochondrial performance and signaling output through direct control of IMS geometry.

## MATERIALS AND METHODS

The following section describes selected key methods; complete and detailed descriptions of all materials and methods are available in Supplementary Materials and Methods.

### *Ccdc127* (SPRIZ) knockout mouse line and genotyping

Cryopreserved sperm from C57BL/6N mice carrying the targeted *Ccdc127^tm1b^* allele (with a lacZ reporter cassette and loxP elements disrupting the gene after exon 2) was obtained from EMMA and rederived to establish the line. After rederivation, the colony was maintained in the heterozygous state and expanded at the University of Ottawa animal facility, where targeted-allele presence was reverified before routine breeding to generate experimental cohorts (wild-type, heterozygous, and homozygous knockout mice). For genotyping, genomic DNA was isolated from ear biopsies using a column-based kit, quantified by spectrophotometry, and diluted as needed for polymerase chain reaction (PCR). PCR reactions used a hot-start master mix and standard components (buffer, MgCl_2_, dNTPs, and polymerase) with primer pairs designed to distinguish wild-type versus *Ccdc127^tm1b^* alleles. Wild-type amplification used a *Ccdc127*-specific forward/reverse primer pair, whereas knockout detection used the same forward primer combined with a cassette-specific reverse primer. All animal procedures were approved by the University of Ottawa Animal Care Committee through Animal Care and Veterinary Service under protocol numbers BMIb-4327-R2 and BMIe-4165-R2 A1. All procedures were conducted in accordance with the guidelines of the Canadian Council on Animal Care, and mice were housed and handled in the University of Ottawa institutional animal facility for breeding and experimental use.

### Quantitative EM and IMS morphometry

Liver tissue pieces were immersion-fixed in paraformaldehyde plus glutaraldehyde in sodium cacodylate buffer and then sectioned on a vibratome. Enhanced membrane contrast was achieved through reduced-osmium chemistry (ferrocyanide/osmium), thiocarbohydrazide treatment, and a second osmium step, followed by graded ethanol dehydration and propylene oxide exchange before Durcupan resin infiltration and polymerization. Ultrathin (∼80 nm) sections were collected on copper grids. HeLa cell pellets underwent analogous fixation and embedding steps, including agarose embedding and vibratome sectioning to match tissue processing. Grids were imaged at 80 kV with a 4K digital camera. Low-magnification fields were used for mitochondrial abundance/area metrics, whereas high-magnification images supported IMS and cristae analyses, with acquisition performed blind to condition and with pixel sizes recorded for each magnification range.

Quantitative morphometry combined semiautomated segmentation with manual curation. For mitochondrial number and area in liver, low-magnification mitochondria were segmented in napari using an empanada/MitoNet model and then manually curated to generate accurate binary masks; Fiji/ImageJ was used to extract area and perimeter and to normalize mitochondrial counts to a standardized tissue area (225 μm^2^). For IMS quantification, high-magnification EM images were preprocessed (grayscale conversion, mean filtering, and Otsu thresholding with minimal uniform adjustment when needed), and then OMM and IMM were manually traced. IMS area was computed as the pixel area between traced boundaries and normalized to OMM perimeter (IMS area/perimeter; fig. S6E). Cristae were traced as regions of interest to compute cristae area, aspect ratio, and cristae density normalized to mitochondrial area (fig. S6D).

### Multiscale MD simulations of SPRIZ-membrane interactions and tethering

Atomistic systems were prepared via CHARMM-GUI (*42*), with protein orientation/embedding guided by the membrane-protein placement tool PPM (*43*) and lipid compositions representing generic OMM mixtures with leaflet asymmetry. Systems were solvated, ionized, and simulated using NAMD 3 (*44*) with CHARMM36/CHARMM36m force fields (*45*). A staged equilibration protocol (minimization plus successive restrained/unrestrained relaxation phases) preceded multiple independent long production runs (400 to 500 ns) per system. Nonbonded interactions used standard cutoffs and particle mesh Ewald electrostatics, with temperature and pressure controlled by Langevin-based schemes.

To access longer timescales and mesoscale behaviors, CG MARTINI 3 simulations (*38*) were performed on (i) the SPRIZ C-terminal domain homodimer positioned above an IMM-like bilayer and (ii) dual-membrane systems modeling SPRIZ tethering between an OMM-like bilayer and an IMM-like bilayer. Loop-mutant variants were constructed in PyMOL, converted to CG representations with martinize2 (including elastic network restraints), and assembled with heterogeneous lipid bilayers generated by insane.py. CG simulations were run in GROMACS (2025.3) (*46*) using standard minimization, extended constant-number, pressure, and temperature equilibration, and multi-microsecond production sampling with multiple replicas to ensure robust statistics. Dual-membrane assemblies included VDAC in the OMM to promote stability and solvent exchange through the OMM as described (*47*), with protein packing/orientation optimized before membrane insertion. Trajectory analyses were performed using MDAnalysis and standard structural metrics (e.g., RMSD, distance/angle distributions) after periodic-boundary correction and recentering, with visualization and movie rendering performed using dedicated molecular visualization pipelines.

### Use of AI

Artificial intelligence (AI)–assisted tools disclosure: We used Grok 4.2 and ChatGPT-5.5 only as editorial aids to improve English language clarity and grammar. We did not use these or other AI tools to generate or modify data or images, or to perform analyses.

## Supplementary Materials

### The PDF file includes:

Supplementary Methods Figs. S1 to S15 Tables S1 and S2 Legends for data files S1 and S2 Legends for movies S1 to S7 References

## References

[1] A. Suomalainen, J. Nunnari, Mitochondria at the crossroads of health and disease. Cell 187, 2601–2627 (2024). 10.1016/j.cell.2024.04.037 38788685

[2] A. S. Monzel, J. A. Enríquez, M. Picard, Multifaceted mitochondria: Moving mitochondrial science beyond function and dysfunction. Nat. Metab. 5, 546–562 (2023). 10.1038/s42255-023-00783-1 PMC10427836 37100996

[3] C. N. Cunningham, J. Rutter, 20,000 Picometers under the OMM: Diving into the vastness of mitochondrial metabolite transport. EMBO Rep. 21, e50071 (2020). 10.15252/embr.202050071 PMC7202207 32329174

[4] S. A. Baker, J. Rutter, Metabolites as signalling molecules. Nat. Rev. Mol. Cell Biol. 24, 355–374 (2023). 10.1038/s41580-022-00572-w 36635456

[5] N. S. Chandel, Mitochondria. Cold Spring Harb. Perspect. Biol. 13, a040543 (2021). 10.1101/cshperspect.a040543 PMC7919390 33649187

[6] B. Cartes-Saavedra, A. Ghosh, G. Hajnóczky, The roles of mitochondria in global and local intracellular calcium signalling. Nat. Rev. Mol. Cell Biol. 26, 456–475 (2025). 10.1038/s41580-024-00820-1 39870977

[7] A. Oberst, C. Bender, D. R. Green, Living with death: The evolution of the mitochondrial pathway of apoptosis in animals. Cell Death Differ. 15, 1139–1146 (2008). 10.1038/cdd.2008.65 PMC2612587 18451868

[8] M. Picard, O. S. Shirihai, Mitochondrial signal transduction. Cell Metab. 34, 1620–1653 (2022). 10.1016/j.cmet.2022.10.008 PMC9692202 36323233

[9] S. Rath, R. Sharma, R. Gupta, T. Ast, C. Chan, T. J. Durham, R. P. Goodman, Z. Grabarek, M. E. Haas, W. H. W. Hung, P. R. Joshi, A. A. Jourdain, S. H. Kim, A. V. Kotrys, S. S. Lam, J. G. McCoy, J. D. Meisel, M. Miranda, A. Panda, A. Patgiri, R. Rogers, S. Sadre, H. Shah, O. S. Skinner, T. L. To, M. A. Walker, H. Wang, P. S. Ward, J. Wengrod, C. C. Yuan, S. E. Calvo, V. K. Mootha, MitoCarta3.0: An updated mitochondrial proteome now with sub-organelle localization and pathway annotations. Nucleic Acids Res. 49, D1541–D1547 (2021). 10.1093/nar/gkaa1011 PMC7778944 33174596

[10] M. Morgenstern, S. B. Stiller, P. Lübbert, C. D. Peikert, S. Dannenmaier, F. Drepper, U. Weill, P. Höß, R. Feuerstein, M. Gebert, M. Bohnert, M. van der Laan, M. Schuldiner, C. Schütze, S. Oeljeklaus, N. Pfanner, N. Wiedemann, B. Warscheid, Definition of a high-confidence mitochondrial proteome at quantitative scale. Cell Rep. 19, 2836–2852 (2017). 10.1016/j.celrep.2017.06.014 PMC5494306 28658629

[11] E. V. Koonin, K. S. Makarova, I. B. Rogozin, L. Davidovic, M. C. Letellier, L. Pellegrini, The rhomboids: A nearly ubiquitous family of intramembrane serine proteases that probably evolved by multiple ancient horizontal gene transfers. Genome Biol. 4, R19 (2003). 10.1186/gb-2003-4-3-r19 PMC153459 12620104

[12] C. Glytsou, E. Calvo, S. Cogliati, A. Mehrotra, I. Anastasia, G. Rigoni, A. Raimondi, N. Shintani, M. Loureiro, J. Vazquez, L. Pellegrini, J. A. Enriquez, L. Scorrano, M. E. Soriano, Optic atrophy 1 is epistatic to the core MICOS component MIC60 in mitochondrial cristae shape control. Cell Rep. 17, 3024–3034 (2016). 10.1016/j.celrep.2016.11.049 PMC5186903 27974214

[13] S. E. Calvo, V. K. Mootha, The mitochondrial proteome and human disease. Annu. Rev. Genomics Hum. Genet. 11, 25–44 (2010). 10.1146/annurev-genom-082509-141720 PMC4397899 20690818

[14] A. K. Kondadi, R. Anand, A. S. Reichert, Cristae membrane dynamics–A paradigm change. Trends Cell Biol. 30, 923–936 (2020). 10.1016/j.tcb.2020.08.008 32978040

[15] O. Daumke, M. van der Laan, Molecular machineries shaping the mitochondrial inner membrane. Nat. Rev. Mol. Cell Biol. 26, 706–724 (2025). 10.1038/s41580-025-00854-z 40369159

[16] T. Bock-Bierbaum, K. Funck, F. Wollweber, E. Lisicki, K. von der Malsburg, A. von der Malsburg, J. Laborenz, J. K. Noel, M. Hessenberger, S. Jungbluth, C. Bernert, S. Kunz, D. Riedel, H. Lilie, S. Jakobs, M. van der Laan, O. Daumke, Structural insights into crista junction formation by the Mic60-Mic19 complex. Sci. Adv. 8, eabo4946 (2022). 10.1126/sciadv.abo4946 PMC9432830 36044574

[17] T. Stephan, C. Brüser, M. Deckers, A. M. Steyer, F. Balzarotti, M. Barbot, T. S. Behr, G. Heim, W. Hübner, P. Ilgen, F. Lange, D. Pacheu-Grau, J. K. Pape, S. Stoldt, T. Huser, S. W. Hell, W. Möbius, P. Rehling, D. Riedel, S. Jakobs, MICOS assembly controls mitochondrial inner membrane remodeling and crista junction redistribution to mediate cristae formation. EMBO J. 39, e104105 (2020). 10.15252/embj.2019104105 PMC7361284 32567732

[18] T. Schnyder, M. Rojo, R. Furter, T. Wallimann, The structure of mitochondrial creatine kinase and its membrane binding properties. Mol. Cell. Biochem. 133-134, 115–123 (1994). 10.1007/BF01267951 7808449

[19] O. Stachowiak, U. Schlattner, M. Dolder, T. Wallimann, Oligomeric state and membrane binding behaviour of creatine kinase isoenzymes: Implications for cellular function and mitochondrial structure. Mol. Cell. Biochem. 184, 141–151 (1998). 9746318

[20] L. Kay, K. Nicolay, B. Wieringa, V. Saks, T. Wallimann, Direct evidence for the control of mitochondrial respiration by mitochondrial creatine kinase in oxidative muscle cells in situ. J. Biol. Chem. 275, 6937–6944 (2000). 10.1074/jbc.275.10.6937 10702255

[21] I. Anastasia, N. Ilacqua, A. Raimondi, P. Lemieux, R. Ghandehari-Alavijeh, G. Faure, S. L. Mekhedov, K. J. Williams, F. Caicci, G. Valle, M. Giacomello, A. D. Quiroga, R. Lehner, M. J. Miksis, K. Toth, T. Q. D. A. Vallim, E. V. Koonin, L. Scorrano, L. Pellegrini, Mitochondria-rough-ER contacts in the liver regulate systemic lipid homeostasis. Cell Rep. 34, 108873 (2021). 10.1016/j.celrep.2021.108873 33730569

[22] B. Gilquin, E. Taillebourg, N. Cherradi, A. Hubstenberger, O. Gay, N. Merle, N. Assard, M.-O. Fauvarque, S. Tomohiro, O. Kuge, J. Baudier, The AAA+ ATPase ATAD3A controls mitochondrial dynamics at the interface of the inner and outer membranes. Mol. Cell. Biol. 30, 1984–1996 (2010). 10.1128/MCB.00007-10 PMC2849464 20154147

[23] Y. Zhao, D. Hu, R. Wang, X. Sun, P. Ropelewski, Z. Hubler, K. Lundberg, Q. Wang, D. J. Adams, R. Xu, X. Qi, ATAD3A oligomerization promotes neuropathology and cognitive deficits in Alzheimer’s disease models. Nat. Commun. 13, 1121 (2022). 10.1038/s41467-022-28769-9 PMC8891325 35236834

[24] K. Can, A. Erstbetreuung, F. Liu, MitoMap-a three-dimensional landscape of mitochondrial architecture, thesis, Charité – Universitätsmedizin Berlin, Berlin, Germany (2025).

[25] Y. Xia, Y. Zhang, Y. Sun, L. He, CCDC127 regulates lipid droplet homeostasis by enhancing mitochondria-ER contacts. Biochem. Biophys. Res. Commun. 683, 149116 (2023). 10.1016/j.bbrc.2023.10.048 37864924

[26] V. Hung, P. Zou, H.-W. Rhee, N. D. Udeshi, V. Cracan, T. Svinkina, S. A. Carr, V. K. Mootha, A. Y. Ting, Proteomic mapping of the human mitochondrial intermembrane space in live cells via ratiometric APEX tagging. Mol. Cell 55, 332–341 (2014). 10.1016/j.molcel.2014.06.003 PMC4743503 25002142

[27] C. Zarges, P. Schepsky, R. A. Rothemann, T. Bock-Bierbaum, A. von der Malsburg, L. Baumann, A. Trifunovic, O. Daumke, M. van der Laan, K. von der Malsburg, J. Riemer, Topology control by a conserved cysteine pair in the OMM-protein CCDC127 enables MICOS interaction. bioRxiv 2025.12.12.693940 [Preprint] (2025); 10.64898/2025.12.12.693940.

[28] S. Hassdenteufel, Y. Bykov, B. Dubreuil, K. Noll, O. Faust, R. Kamyshinsky, M. Itkin, N. Cohen, S. Malitsky, Y. Peleg, S. Albeck, K. von der Malsburg, D. Fass, M. van der Laan, M. Schuldiner, A multiplexed approach for genetic screening of human cells by electron microscopy uncovers a critical effector of mitochondrial cristae shape. bioRxiv 2025.11.15.688601 [Preprint] (2025); 10.1101/2025.11.15.688601.

[29] T. Bock-Bierbaum, K. von der Malsburg, A. K. Natarajan, C. Zarges, K. C. Akkaya, A. Aladawi, K. Noll, S. Jungbluth, C. Schirra, Y. Zhu, N. Cremer, C. Bernert, F. Liu, M. Lehmann, J. Riemer, M. van der Laan, O. Daumke, Structural studies suggest CCDC127 as a novel membrane contact site protein in the mitochondrial intermembrane space. bioRxiv 2025.12.11.693622 [Preprint] (2025); 10.64898/2025.12.11.693622.

[30] N. Ilacqua, I. Anastasia, D. Aloshyn, R. Ghandehari-Alavijeh, E. A. Peluso, M. C. Brearley-Sholto, L. V. Pellegrini, A. Raimondi, T. Q. de Aguiar Vallim, L. Pellegrini, Expression of Synj2bp in mouse liver regulates the extent of wrappER-mitochondria contact to maintain hepatic lipid homeostasis. Biol. Direct 17, 37 (2022). 10.1186/s13062-022-00344-8 PMC9717519 36457006

[31] N. Ilacqua, I. Anastasia, A. Raimondi, P. Lemieux, T. Q. de Aguiar Vallim, K. Toth, E. V. Koonin, L. Pellegrini, A three-organelle complex made by wrappER contacts with peroxisomes and mitochondria responds to liver lipid flux changes. J. Cell Sci. 135, jcs259091 (2021). 10.1242/jcs.259091 PMC8627550 34672330

[32] N. Ilacqua, I. Anastasia, L. Pellegrini, Isolation and analysis of fractions enriched in WrappER-associated mitochondria from mouse liver. STAR Protoc. 2, 100752 (2021). 10.1016/j.xpro.2021.100752 PMC8377487 34458870

[33] T. B. Ansell, R. A. Corey, L. V. Viti, M. Kinnebrew, R. Rohatgi, C. Siebold, M. S. P. Sansom, The energetics and ion coupling of cholesterol transport through Patched1. Sci. Adv. 9, eadh1609 (2023). 10.1126/sciadv.adh1609 PMC10446486 37611095

[34] M. Chavent, A. L. Duncan, M. S. P. Sansom, Molecular dynamics simulations of membrane proteins and their interactions: From nanoscale to mesoscale. Curr. Opin. Struct. Biol. 40, 8–16 (2016). 10.1016/j.sbi.2016.06.007 PMC5404110 27341016

[35] W. Pezeshkian, S. J. Marrink, Simulating realistic membrane shapes. Curr. Opin. Cell Biol. 71, 103–111 (2021). 10.1016/j.ceb.2021.02.009 33721706

[36] L. Borges-Araújo, I. Patmanidis, A. P. Singh, L. H. S. Santos, A. K. Sieradzan, S. Vanni, C. Czaplewski, S. Pantano, W. Shinoda, L. Monticelli, A. Liwo, S. J. Marrink, P. C. T. Souza, Pragmatic coarse-graining of proteins: Models and applications. J. Chem. Theory Comput. 19, 7112–7135 (2023). 10.1021/acs.jctc.3c00733 37788237

[37] V. Corradi, B. I. Sejdiu, H. Mesa-Galloso, H. Abdizadeh, S. Y. Noskov, S. J. Marrink, D. P. Tieleman, Emerging diversity in lipid–protein interactions. Chem. Rev. 119, 5775–5848 (2019). 10.1021/acs.chemrev.8b00451 PMC6509647 30758191

[38] P. C. T. Souza, R. Alessandri, J. Barnoud, S. Thallmair, I. Faustino, F. Grünewald, I. Patmanidis, H. Abdizadeh, B. M. H. Bruininks, T. A. Wassenaar, P. C. Kroon, J. Melcr, V. Nieto, V. Corradi, H. M. Khan, J. Domański, M. Javanainen, H. Martinez-Seara, N. Reuter, R. B. Best, I. Vattulainen, L. Monticelli, X. Periole, D. P. Tieleman, A. H. de Vries, S. J. Marrink, Martini 3: A general purpose force field for coarse-grained molecular dynamics. Nat. Methods 18, 382–388 (2021). 10.1038/s41592-021-01098-3 PMC12554258 33782607

[39] J. Jumper, R. Evans, A. Pritzel, T. Green, M. Figurnov, O. Ronneberger, K. Tunyasuvunakool, R. Bates, A. Žídek, A. Potapenko, A. Bridgland, C. Meyer, S. A. A. Kohl, A. J. Ballard, A. Cowie, B. Romera-Paredes, S. Nikolov, R. Jain, J. Adler, T. Back, S. Petersen, D. Reiman, E. Clancy, M. Zielinski, M. Steinegger, M. Pacholska, T. Berghammer, S. Bodenstein, D. Silver, O. Vinyals, A. W. Senior, K. Kavukcuoglu, P. Kohli, D. Hassabis, Highly accurate protein structure prediction with AlphaFold. Nature 596, 583–589 (2021). 10.1038/s41586-021-03819-2 PMC8371605 34265844

[40] M. Morgenstern, C. D. Peikert, P. Lübbert, I. Suppanz, C. Klemm, O. Alka, C. Steiert, N. Naumenko, A. Schendzielorz, L. Melchionda, W. W. D. Mühlhäuser, B. Knapp, J. D. Busch, S. B. Stiller, S. Dannenmaier, C. Lindau, M. Licheva, C. Eickhorst, R. Galbusera, R. M. Zerbes, M. T. Ryan, C. Kraft, V. Kozjak-Pavlovic, F. Drepper, S. Dennerlein, S. Oeljeklaus, N. Pfanner, N. Wiedemann, B. Warscheid, Quantitative high-confidence human mitochondrial proteome and its dynamics in cellular context. Cell Metab. 33, 2464–2483.e18 (2021). 10.1016/j.cmet.2021.11.001 PMC8664129 34800366

[41] M. K. Kucińska, J. Fedry, C. Galli, D. Morone, A. Raimondi, T. Soldà, F. Förster, M. Molinari, TMX4-driven LINC complex disassembly and asymmetric autophagy of the nuclear envelope upon acute ER stress. Nat. Commun. 14, 3497 (2023). 10.1038/s41467-023-39172-3 PMC10264389 37311770

[42] S. Jo, T. Kim, V. G. Iyer, W. Im, CHARMM-GUI: A web-based graphical user interface for CHARMM. J. Comput. Chem. 29, 1859–1865 (2008). 10.1002/jcc.20945 18351591

[43] M. A. Lomize, I. D. Pogozheva, H. Joo, H. I. Mosberg, A. L. Lomize, OPM database and PPM web server: Resources for positioning of proteins in membranes. Nucleic Acids Res. 40, D370–D376 (2012). 10.1093/nar/gkr703 PMC3245162 21890895

[44] J. C. Phillips, D. J. Hardy, J. D. C. Maia, J. E. Stone, J. V. Ribeiro, R. C. Bernardi, R. Buch, G. Fiorin, J. Hénin, W. Jiang, R. McGreevy, M. C. R. Melo, B. K. Radak, R. D. Skeel, A. Singharoy, Y. Wang, B. Roux, A. Aksimentiev, Z. Luthey-Schulten, L. V. Kalé, K. Schulten, C. Chipot, E. Tajkhorshid, Scalable molecular dynamics on CPU and GPU architectures with NAMD. J. Chem. Phys. 153, 044130 (2020). 10.1063/5.0014475 PMC7395834 32752662

[45] J. Lee, X. Cheng, J. M. Swails, M. S. Yeom, P. K. Eastman, J. A. Lemkul, S. Wei, J. Buckner, J. C. Jeong, Y. Qi, S. Jo, V. S. Pande, D. A. Case, C. L. Brooks III, A. D. MacKerell, J. B. Klauda, W. Im, Charmm-gui input generator for namd, gromacs, amber, openmm, and charmm/openmm simulations using the charmm36 additive force field. J. Chem. Theory Comput. 12, 405–413 (2016). 10.1021/acs.jctc.5b00935 PMC4712441 26631602

[46] M. J. Abraham, T. Murtola, R. Schulz, S. Páll, J. C. Smith, B. Hess, E. Lindahl, GROMACS: High performance molecular simulations through multi-level parallelism from laptops to supercomputers. SoftwareX 1-2, 19–25 (2015).

[47] C. M. Brown, M. S. S. Westendorp, R. Zarmiento-Garcia, J. A. Stevens, B. M. H. Bruininks, S. L. Rouse, S. J. Marrink, T. A. Wassenaar, An integrative modelling approach to the mitochondrial cristae. Commun. Biol. 8, 972 (2025). 10.1038/s42003-025-08381-5 PMC12216777 40596661

[48] C. Frezza, S. Cipolat, L. Scorrano, Organelle isolation: Functional mitochondria from mouse liver, muscle and cultured fibroblasts. Nat. Protoc. 2, 287–295 (2007). 10.1038/nprot.2006.478 17406588

[49] M. Triolo, S. Wade, N. Baker, M. Khacho, Evaluating mitochondrial length, volume, and cristae ultrastructure in rare mouse adult stem cell populations. STAR Protoc. 4, 102107 (2023). 10.1016/j.xpro.2023.102107 PMC9943866 36853728

[50] P. Kotchetkov, B. Lacoste, Volumetric analysis of the blood–brain barrier after ischemic stroke by electron tomography in mice. Microcirculation 32, e70025 (2025). 10.1111/micc.70025 PMC12450457 40975889

[51] Y. Gao, J. Lee, I. P. S. Smith, H. Lee, S. Kim, Y. Qi, J. B. Klauda, G. Widmalm, S. Khalid, W. Im, CHARMM-GUI supports hydrogen mass repartitioning and different protonation states of phosphates in lipopolysaccharides. J. Chem. Inf. Model. 61, 831–839 (2021). 10.1021/acs.jcim.0c01360 PMC7902386 33442985

[52] T. Darden, L. Perera, L. Li, L. Pedersen, New tricks for modelers from the crystallography toolkit: The particle mesh Ewald algorithm and its use in nucleic acid simulations. Structure 7, R55–R60 (1999). 10.1016/s0969-2126(99)80033-1 10368306

[53] S. Miyamoto, P. A. Kollman, Settle: An analytical version of the SHAKE and RATTLE algorithm for rigid water models. J. Comput. Chem. 13, 952–962 (1992).

[54] J.-P. Ryckaert, G. Ciccotti, H. J. C. Berendsen, Numerical integration of the cartesian equations of motion of a system with constraints: Molecular dynamics of n-alkanes. J. Comput. Phys. 23, 327–341 (1977).

[55] H. C. Andersen, Rattle: A “velocity” version of the shake algorithm for molecular dynamics calculations. J. Comput. Phys. 52, 24–34 (1983).

[56] R. J. Loncharich, B. R. Brooks, R. W. Pastor, Langevin dynamics of peptides: The frictional dependence of isomerization rates of N-acetylalanyl-N’-methylamide. Biopolymers 32, 523–535 (1992). 10.1002/bip.360320508 1515543

[57] J. C. Phillips, R. Braun, W. Wang, J. Gumbart, E. Tajkhorshid, E. Villa, C. Chipot, R. D. Skeel, L. Kalé, K. Schulten, Scalable molecular dynamics with NAMD. J. Comput. Chem. 26, 1781–1802 (2005). 10.1002/jcc.20289 PMC2486339 16222654

[58] P. C. Kroon, F. Grünewald, J. Barnoud, M. van Tilburg, C. Brasnett, P. C. T. Souza, T. A. Wassenaar, S. J. Marrink, Martinize2 and Vermouth provide a unified framework for molecular topology generation. eLife 12, RP90627 (2025). 10.7554/eLife.90627 PMC12634043 41263305

[59] M. Parrinello, A. Rahman, Polymorphic transitions in single crystals: A new molecular dynamics method. J. Appl. Phys. 52, 7182–7190 (1981).

[60] M. S. S. Westendorp, J. A. Stevens, B. M. H. Bruininks, S.-J. Marrink, Bentopy: Building molecular dynamics simulations with cellular complexity and scale, Zenodo (2024); 10.5281/ZENODO.13818758.

[61] N. Michaud-Agrawal, E. J. Denning, T. B. Woolf, O. Beckstein, MDAnalysis: A toolkit for the analysis of molecular dynamics simulations. J. Comput. Chem. 32, 2319–2327 (2011). 10.1002/jcc.21787 PMC3144279 21500218

[62] Brady Johnston, Pardhav Maradani, Johannes Elferich, Russell B. Davidson, Yuxuan Zhuang, Yinying Yao, zachcp, Thibault Tubiana, Rich, Patrick Kunzmann, Olive Laprevote, TheJeran, ludovic autin, Kai Niklas Spauszus, Domenico Marson, Blastorios, Alyssa Travitz, Marcelo C. R. Melo, Hampton Copeland, elija feigl, bion howard, Tyler Reddy, Prasun Kumar, Louis Colson, Joyce Kim, Jessica A. Nash, Jamie Rowe, James Hooker, Emmanuel Ferdman, Brener Ramos, BradyAJohnston/MolecularNodes: v4.5.10, version v4.5.10, Zenodo (2026); 10.5281/ZENODO.18385790.

[63] T. Schmidt, P. Samaras, M. Frejno, S. Gessulat, M. Barnert, H. Kienegger, H. Krcmar, J. Schlegl, H.-C. Ehrlich, S. Aiche, B. Kuster, M. Wilhelm, ProteomicsDB. Nucleic Acids Res. 46, D1271–D1281 (2018). 10.1093/nar/gkx1029 PMC5753189 29106664

[64] P. Samaras, T. Schmidt, M. Frejno, S. Gessulat, M. Reinecke, A. Jarzab, J. Zecha, J. Mergner, P. Giansanti, H.-C. Ehrlich, S. Aiche, J. Rank, H. Kienegger, H. Krcmar, B. Kuster, M. Wilhelm, ProteomicsDB: A multi-omics and multi-organism resource for life science research. Nucleic Acids Res. 48, gkz974 (2019). 10.1093/nar/gkz974 PMC7145565 31665479

[65] L. Lautenbacher, P. Samaras, J. Muller, A. Grafberger, M. Shraideh, J. Rank, S. T. Fuchs, T. K. Schmidt, M. The, C. Dallago, H. Wittges, B. Rost, H. Krcmar, B. Kuster, M. Wilhelm, ProteomicsDB: Toward a FAIR open-source resource for life-science research. Nucleic Acids Res. 50, D1541–D1552 (2022). 10.1093/nar/gkab1026 PMC8728203 34791421

[66] M. Wilhelm, J. Schlegl, H. Hahne, A. M. Gholami, M. Lieberenz, M. M. Savitski, E. Ziegler, L. Butzmann, S. Gessulat, H. Marx, T. Mathieson, S. Lemeer, K. Schnatbaum, U. Reimer, H. Wenschuh, M. Mollenhauer, J. Slotta-Huspenina, J.-H. Boese, M. Bantscheff, A. Gerstmair, F. Faerber, B. Kuster, Mass-spectrometry-based draft of the human proteome. Nature 509, 582–587 (2014). 10.1038/nature13319 24870543

