# Supplementary material for: SPRIZ (CCDC127) protein conserved in deuterostome animals is a rigid OMM–IMM tether that locally sets mitochondrial IMS volume
Source: Sci Adv. 2026 Oct 14;12(42):eaef3522. doi: 10.1126/sciadv.aef3522 (PMC13666014; doi:10.1126/sciadv.aef3522)
Supplement: Supplementary file 1 [file sciadv.aef3522_sm.pdf]

Science Advances
AAAS

Supplementary Materials for

SPRIZ (CCDC127) protein conserved in deuterostome animals is a rigid OMM–IMM tether that locally sets mitochondrial IMS volume

Raman Shulinski *et al.*

Corresponding author: Luca Pellegrini, luca.pellegrini@uottawa.ca

Sci. Adv. **12**, eaef3522 (2026)
DOI: 10.1126/sciadv.aef3522

The PDF file includes:

Supplementary Methods
Figs. S1 to S15
Tables S1 and S2
Legends for data files S1 and S2
Legends for movies S1 to S7
References

Other Supplementary Material for this manuscript includes the following:

Data files S1 and S2
Movies S1 to S7

Supplementary Methods

1. Mouse Genetics and Animal Procedures

i. Generation of *Ccdc127* knockout mice
ii. Mouse genomic DNA extraction from ear biopsies
iii. Mouse genotyping PCR
iv. Animal facility transfer and ethical approvals

2. SPRIZ characterization (biochemical studies)

i. Carbonate extraction assay
ii. Protease protection assay
iii. BN-PAGE
iv. Chemical crosslinking assay
v. Co-immunoprecipitation

3. SPRIZ characterization (functional studies)

i. Calcium retention capacity (mouse liver mitochondria-enriched fractions)
ii. Mitochondrial swelling assay (mouse liver mitochondria-enriched fractions)
iii. High resolution respirometry and fatty acid oxidation assay (mouse liver mitochondria-enriched fractions)
iv. Mitochondrial calcium uptake (HeLa cells)

4. Cell Culture, transfection, and imaging

i. Cell cultures
ii. Generation of *Ccdc127*$^{-/-}$ HeLa cells
iii. Transient plasmid transfection
iv. Cell preparation for imaging
v. Cell imaging

5. Transmission electron microscopy (TEM)

i. TEM: sample preparation of mouse liver tissue
ii. TEM: sample preparation of HeLa cells
iii. TEM: image acquisition
iv. Mitochondrial morphometric EM analysis
v. Mitochondrial intermembrane space EM analysis
vi. *Cristae* ultrastructure EM analysis
vii. Quantitative morphometry analysis of mitochondria-associated membranes (MAMs)

6. Molecular dynamics simulation

i. Atomistic Molecular Dynamics Simulations
ii. Coarse-grained simulations of SPRIZ C-terminal domain
iii. Coarse-grained simulation of SPRIZ homodimer tethering OMM and IMM
iv. Molecular dynamics trajectory analysis

7. Other procedures

i. Isolation of mouse liver wrappER-associated mitochondria- and mitochondria-enriched fractions
ii. AAV8-mediated gene delivery
iii. Proteomic analysis
iv. Expression plasmid construction and purification
v. SDS-PAGE and immunoblotting
vi. *In vitro* transcription and translation
vii. SPRIZ (CCDC127) evolutionary analysis. Identification of SPRIZ homologs and phylogenetic analysis
viii. Electron tomography acquisition and quantification
ix. Cryo-ET: Tomogram 3D reconstruction
x. Radiotracer assay of IMS-accessible volume

1. Mouse Genetics and Animal Procedures

- Generation of *Ccdc127* knockout mice
- Mouse genomic DNA extraction from ear biopsies
- Mouse genotyping PCR
- Animal facility transfer and ethical approvals

Generation of *Ccdc127* knockout mice. Cryopreserved sperm from C57BL/6N mice carrying the *Ccdc127*^{tm1b} allele was obtained from the European Mouse Mutant Archive (EMMA #14537). The knockout allele features a lacZ reporter cassette and *loxP* sites disrupting the gene after exon 2 (Fig. 1B).

The mouse line was rederived at Charles River Laboratories and established in the heterozygous state. The line was subsequently shipped to the Animal Facility of the University of Ottawa, where additional genotyping verification was performed to confirm the targeted allele. Heterozygous animals were then used to establish a breeding colony, from which both heterozygous and homozygous knockout mice were generated and maintained for experimental use.

Mouse genomic DNA extraction. Genomic DNA was isolated from mouse ear biopsies using the G-SYNC™ DNA Isolation Kit (FroggaBio #GS100) according to the manufacturer's instructions. DNA concentration and purity were assessed by spectrophotometry, and samples were diluted as needed for subsequent PCR analysis.

Mouse genotyping by PCR. Genotyping PCR was performed using Platinum™ Hot Start PCR Master Mix (2×) (Invitrogen #13001013), supplemented with additional components as follows. Reactions (20 µL final volume) were carried out in a SimpliAmp™ thermal cycler (Applied Biosystems). Each reaction contained approximately 50–100 ng genomic DNA, 1× PCR buffer, 1.5 mM $MgCl_2$, 0.2 mM dNTPs (FroggaBio #D-5-10), 0.2 µL Platinum™ Taq DNA polymerase, and 0.2 µM of each primer.

Primer sequences:

- Wild-type *Ccdc127* allele: Ccdc127_96666_F (5′-TGCTCATTCTTCAAACCCCAG-3′) and Ccdc127_96666_R (5′-AGTGTTGTGTGGGAGGGAGG-3′)
- Knockout tm1b allele: Ccdc127_96666_F (5′-TGCTCATTCTTCAAACCCCAG-3′) and CAS_R1_Term (5′-TCGTGGTATCGTTATGCGCC-3′)

Amplification: initial denaturation at 94 °C for 5 min, followed by 34 cycles of 94 °C for 30 s, 58 °C for 30 s, 72 °C for 45 s, and a final extension at 72 °C for 5 min, with hold at 12 °C.

PCR products were resolved on 2% agarose gels in 1× TBE buffer, using agarose (Invitrogen #16500-500), 10× TBE buffer (Invitrogen #15581-044), and SafeView™ Classic DNA stain (ABM, #G108). Fragment sizes were determined using a Bio-Helix 50 bp Plus DNA ladder (FroggaBio #DM017-R500).

Animal housing and ethical approvals. Animals were housed at the Animal Facility of the University of Ottawa for breeding and experimental use. All animal protocols were approved by the University of Ottawa Animal Care Committee and conducted in accordance with the guidelines of the Canadian Council on Animal Care (CCAC).

2. SPRIZ characterization (biochemical studies)

- Carbonate extraction assay
- Protease protection assay
- BN-PAGE
- Chemical crosslinking assay
- Co-immunoprecipitation

SPRIZ membrane integration by carbonate extraction. WrappER-associated mitochondrial (WAM) fractions were isolated as described below. Pellets were resuspended in 1 mL of 0.1%

Triton X-100 in distilled water supplemented with protease inhibitor cocktail (PIC; Thermo Fisher Scientific #78446; 1× final) and incubated on ice for 30 min. Samples were subsequently frozen at −30 °C for approximately 1 h and thawed on ice. A 300 µL aliquot of the WAM fraction was mixed with 4× Tris-buffered saline (TBS) and stored at −30 °C as an input control.

For alkaline extraction, 700 µL of WAM was adjusted to a final volume of 3.0 mL by the addition of sodium carbonate buffer (final concentration 100 mM Na_2CO_3, pH 11.4) supplemented with PIC. Samples were incubated on ice for 30 min and subsequently centrifuged at 45,000 rpm for 1 h 5 min at 4 °C using a Beckman SW61 Ti rotor. Following ultracentrifugation, the supernatant containing carbonate-soluble proteins was carefully collected and concentrated using 3 kDa molecular weight cutoff centrifugal filters (Millipore Sigma #UFC800308). The pellet corresponding to carbonate-resistant membrane proteins was resuspended in 200 µL of distilled water supplemented with 1× RIPA buffer and PIC. Fractions were processed for downstream SDS–PAGE and immunoblot analysis.

SPRIZ topology by protease protection assay. Protease protection assays were performed in two experimental variants. Fresh mouse liver WAM fractions were isolated and resuspended in 1 mL of EB10 buffer (described below). For assays performed with constant incubation time and varying protease concentration, a 2.5% trypsin stock solution was serially diluted and incubated with 45 µL of WAM fraction for 5 min. For assays performed with constant trypsin concentration and varying incubation times, 5 µL of 0.5% trypsin was added to the WAM fraction and reactions were terminated at the indicated time points. In all cases, reactions were quenched by the addition of 50 µL of 2× NuPAGE™ LDS Sample Buffer (derived from 4× stock; Thermo Fisher Scientific #NP0007) supplemented with 0.1 M DTT, followed by heating at 95 °C for 5 min.

SPRIZ dimerization by BN-PAGE. Blue Native polyacrylamide gel electrophoresis (BN-PAGE) was performed using freshly isolated mitochondrial pellets. Pellets were resuspended in EB10 buffer (described below) and solubilized with digitonin (Invitrogen #BN2008) at detergent-to-protein ratios of 2 g, 4 g, or 8 g digitonin per gram of total protein. Samples were incubated on ice for 30 min and subsequently clarified by centrifugation at 20,000 × g for 15 min at 4 °C. Protein concentration of the supernatant was determined using the Bradford protein assay.

Coomassie G-250 (5%; Invitrogen #BN2008) was added to the solubilized samples at a final amount corresponding to one-quarter of the detergent concentration. Samples were then mixed with NativePAGE™ Sample Buffer (4×; Invitrogen #BN2003) and loaded onto NativePAGE™ Bis-Tris Mini Protein Gels (4–16%; Invitrogen #BN1002BOX). Electrophoresis was performed at 150 V for 2 h using cathode and anode buffers prepared from NativePAGE™ Running Buffer (20×; Invitrogen #BN2001) according to the manufacturer's instructions.

Following electrophoresis, proteins were transferred to PVDF membranes for 1 h at 100 V and subsequently analyzed by immunoblotting as described above.

SPRIZ dimerization by chemical crosslinking assay. Chemical crosslinking of mitochondrial proteins was performed using freshly isolated mitochondria-enriched fractions (see below).

Mitochondria were resuspended in 100 µL of EB10 (composition specified below) buffer supplemented with the indicated concentration of the thiol-reactive crosslinker bismaleimidohexane dissolved in DMSO (BMH; Thermo Fisher Scientific #22330). Samples were incubated for 2 h at room temperature with gentle agitation. Crosslinking reactions were terminated by the addition of an equal volume of 2× NuPAGE™ LDS sample buffer supplemented with 100 mM DTT. Samples were subsequently heated and analyzed by SDS–PAGE and immunoblotting.

SPRIZ dimerization by co-IP. Co-immunoprecipitation experiments were performed using transiently transfected HEK293T cells cultured in 6-well plates. Cells were harvested and lysed in 500 µL of immunoprecipitation buffer (IPB) containing 150 mM NaCl, 10 mM Tris-HCl, 1 mM EDTA, 1 mM EGTA (pH 8.0), 1% Triton X-100, 0.5% NP-40, and protease inhibitor cocktail (PIC; Thermo Fisher Scientific, cat. no. 78446; 1× final). Cell lysates were clarified by centrifugation at 15,000 × g for 10 min at 4 °C, and the resulting supernatants were used for immunoprecipitation.

For each reaction, 100 µL of clarified lysate was incubated with the indicated primary antibody at a 1:100 dilution overnight at 4 °C with constant rotation. Subsequently, 25 µL of Protein A magnetic beads (Cell Signaling Technology #S1425S) were added and incubated for 2 h at room temperature with gentle agitation. Beads were collected using a magnetic stand and washed twice with 1× RIPA buffer, followed by a final 15-min incubation in 1× RIPA buffer at room temperature. Bound proteins were eluted by incubation with 2× NuPAGE™ LDS sample buffer supplemented with 100 mM DTT and analyzed by SDS–PAGE and immunoblotting.

3. SPRIZ characterization (functional studies)

- Calcium retention capacity (mouse liver mitochondria-enriched fractions)
- Mitochondrial swelling assay (mouse liver mitochondria-enriched fractions)
- High resolution respirometry and fatty acid oxidation assay (mouse liver mitochondria-enriched fractions)
- Mitochondrial calcium uptake (HeLa cells)

Mitochondrial calcium retention capacity (CRC) was assessed using freshly isolated mouse liver mitochondria prepared as described previously (48). Mitochondrial pellets were resuspended in 1 mL of CRC buffer containing 130 mM KCl, 10 mM MOPS–Tris, 10 µM EGTA–Tris, 5 mM glutamate, 2.5 mM malate, 1 mM inorganic phosphate, and 1 µM Calcium Green™-5N hexapotassium salt (Thermo Fisher Scientific #C3737). Protein concentration was determined using the Bradford protein assay, and mitochondrial suspensions were diluted to a final concentration of 1 mg/mL.

For CRC measurements, 1 mL of the mitochondrial suspension was transferred to individual wells of a 12-well plate and fluorescence was monitored at 37 °C using a Synergy™ H1 microplate reader (BioTek) equipped with an automated injector. Calcium Green-5N fluorescence was

recorded using excitation at 506 nm and emission at 532 nm. Sequential calcium pulses were delivered by automated injection of 10 µL of 2 mM or 10 mM $CaCl_2$ every 2 min until a rapid increase in extramitochondrial calcium fluorescence was observed, indicating mitochondrial permeability transition pore opening. For statistical analysis of calcium peaks, the data were normalized to the control by setting the average control value for each peak to 100%.

Mitochondrial swelling was assessed in parallel under identical experimental conditions. Alamethicin (Sigma-Aldrich #A4665) was injected at the indicated concentration using the automated injector, and changes in mitochondrial volume were subsequently monitored by measuring light scattering at 540 nm over a 20-min period, as specified in the corresponding figure legends. Ca^{2+}-induced mitochondrial swelling was triggered by the addition of 500 µM $CaCl_2$. In negative-control reactions, $CaCl_2$ was omitted. Complete swelling was induced by the addition of 10 µM alamethicin.

Mitochondrial calcium uptake. HeLa cells expressing the mitochondria-targeted calcium reporter mtRCaMP were loaded with 2 µM Fura 2-AM (Invitrogen, # F1221) cytoplasmic calcium indicator in the presence of 0.03% Pluronic F127 and 100 µM sulfinpyrazone at 35 °C for 30 min using a 2% BSA extracellular medium (ECM) consisting of 121 mM NaCl, 5 mM $NaHCO_3$, 4.7 mM KCl, 1.2 mM KH_2PO_4, 1.2 mM $MgSO_4$, 10 mM glucose, and 10 mM Na-HEPES, pH 7.4. Cells were washed with Ca^{2+}-free 0.25% BSA ECM medium and mounted into microscope loading chambers on a thermostat-controlled (37 °C) stage of an Olympus IX70 inverted microscope with LambdaDG4 wavelength-switch xenon light source (Sutter, Novato, CA) optimized by custom Spectralyzer software. Fura2 fluorescence was recorded with 340/11 nm and 380/11 nm excitation while 577/25 nm excitation was used for the mtRCaMP using dual-band dichroic and emission filters (Chroma, customized set 59022). Images were collected on a high quantum efficiency cooled CCD camera every 0.5 s. $[Ca^{2+}]c$ measurements with Fura2 were calibrated *in vitro* by adding 1 mM $CaCl_2$, followed by 10 mM EGTA/Tris, pH 8.5. Basal fluorescent levels were measured for 60 s, after which calcium release was evoked by addition of 100 µM or 500 µM histamine. To determine $[Ca^{2+}]$, the following formula was used:

$$[Ca^{2+}]_{Cyto}\ (nM) = K_d * \frac{R - R_{min}}{R_{max} - R} * \frac{F_{max}^{380}}{F_{min}^{380}}$$

Where K_d is the Ca^{2+} dissociation constant (224 nM), R is the ratio of the fluorescence intensities at 340/380 nm excitation, R_{min} and R_{max} are the fluorescence ratios in Ca^{2+}-free and Ca^{2+}-saturated conditions, respectively. F_{max} is the ratio of fluorescence intensities measured at 380 nm excitation upon EGTA addition, and F_{min} is the ratio of fluorescence intensities measured at 380 nm upon Ca^{2+} addition. $[Ca^{2+}]$ is measured over regions of interest (cells) and cells with cytosolic distributed mtRCaMP were excluded from analyses. To determine coupling time, the delay between ½ max $[Ca^{2+}]c$ and ½ max $[Ca^{2+}]m$ was calculated for each cell.

High resolution respirometry and fatty acid oxidation assay. Liver mitochondria were isolated using an adapted protocol (48). Briefly, liver was quickly excised, rinsed in an isotonic buffer 1 [10 mM Tris–MOPS, 1 mM EGTA, and 200 mM sucrose (pH 7.4)] and then minced and resuspended

in 2-3 mL of the buffer for tissue homogenization, which was performed using a glass–Teflon homogenizer. Homogenates were centrifuged at 800 × g for 10 min at 4 °C. The supernatant was collected, transferred to a new tube and centrifuged at 8,600 × g for 10 min at 4 °C. The mitochondrial pellet was resuspended in 1–2 mL of buffer and centrifuged again at 8,600 × g for 10 min at 4 °C. The final mitochondrial pellet was resuspended in the buffer and kept on ice for immediate use. Mitochondrial protein concentration was determined using a Bradford protein assay. Characteristics of oxidative phosphorylation were then measured using aliquots of mitochondria (equivalent of 200 µg) in high-resolution respirometers (O2k; Oroboros, Innsbruck, Austria). Oxygen consumption (J_{O_2}) was assessed using SUIT protocols optimized for fatty acid oxidation and electron transport system respiration. All experiments were performed in MiR05 respiration buffer at 37 °C.

Two distinct protocols were used – one for the characterization of mitochondrial respiratory capacity under complex I and II substrate conditions (ETS), and the other to provide detailed bioenergetic information on fatty-acid oxidation (FAO) supported respiration and electron transport system function.

In the ETS protocol, substrates and inhibitors were also added sequentially to generate the respiratory states shown in the ETS panel. Following the measurement of basal respiration, complex I supported respiration (CI Leak and OXPHOS) was assessed in the presence of malate (1 mM), pyruvate (5 mM), and glutamate (10 mM), before and after ADP/Mg^{2+} (5 mM) addition. Succinate (10 mM) was added to assess CI + CII OXPHOS respiration. Oligomycin (2.5 µM) was added to determine CI + CII Leak. Maximal ETS capacity (Max) was assessed by sequential additions of FCCP (0.25 µM per bolus). Non-mitochondrial respiration (Non-Mito) was assessed following antimycin A (2.5 µM) addition. Respiratory control ratio (RCR) was calculated as the ratio of ADP-stimulated respiration (state 3) to leak respiration (state 4), serving as an indicator of mitochondrial function and/or the quality of the mitochondrial preparation. Oxygen flux was normalized to mitochondrial protein content and expressed per milligram of mitochondrial protein.

In the FAO protocol, substrates and inhibitors were added sequentially to generate the respiratory states shown in the FAO panel. Fatty acid oxidation was initiated by the addition of malate (0.1 mM) and octanoylcarnitine (10–200 µM; MOcar and OCar states). ADP/Mg^{2+} (5 mM) was then added to stimulate OXPHOS, followed by succinate (10 mM; Succ) to support convergent electron flow. Oligomycin (2.5 µM; Oligo) was used to inhibit ATP synthase (to assess non-phosphorylating respiration). Maximal electron transport system capacity was determined by stepwise additions of FCCP (0.25 µM boluses), and residual oxygen consumption was measured following inhibition of complex III with antimycin A (2.5 µM; AMA).

4. Cell Culture, transfection, and imaging

- Cell cultures
- Generation of *Ccdc127*$^{-/-}$ HeLa cells
- Transient plasmid transfection
- Cell preparation for imaging
- Cell imaging

Human cell lines. HeLa (#CCL-2) and HEK293T (#CRL-3216) cells were obtained from the American Type Culture Collection (ATCC). Cells were maintained in Dulbecco's modified Eagle's medium (DMEM; WISENT #319-005 CL) supplemented with 5% fetal bovine serum (FBS; WISENT #080-150) and 1× penicillin–streptomycin solution (Corning #30-002-CI) at 37 °C in a humidified incubator with 5% CO_2.

***Ccdc127* knockout and wild-type control HeLa cell pools**. *Ccdc127* knockout and wild-type control HeLa cell pools were generated commercially by EditCo Bio (Redwood City, CA). The CCDC127 knockout pool was produced via a multi-guide CRISPR/Cas9 strategy targeting exon 3 of CCDC127 (Ensembl transcript ENST00000296824.4) using three guide RNAs (5′-CAUCAUGUCAGGUUGGAUU-3′, 5′-AAGAGAGAAGCCUACCGU-3′, and 5′-ACGCCGAUUUUCUGAGAUCA-3′) with predicted cut sites at chr5:205954, chr5:205903, and chr5:205848. A parallel mock-transfected wild-type HeLa pool served as the isogenic control. Both pools were supplied as cryopreserved stocks at passage 2, with pre-cryopreservation viability >80% and negative mycoplasma testing.

Knockout validation was performed by the manufacturer through targeted NGS amplicon sequencing of the edited region (9,001 reads; Q30 = 90.7%) using the primers 5′-TCCAAGGACAACTGAGCGAC-3′ (forward) and 5′-GCCGAGGTCTTAAGCTTTTACTG-3′ (reverse). The CCDC127 knockout pool showed 0% remaining wild-type alleles, 100% editing efficiency, and a 100% KO score. The editing profile was dominated by large deletions, as expected from the multi-guide approach; genotypes contributing <5% of reads were excluded by the provider's analysis pipeline. Cells were thawed, recovered, and cultured according to the manufacturer's recommended conditions. These fully validated pools were used for mitochondrial calcium uptake experiments.

Transfection. HEK293T cells were seeded at ~20% confluency following overnight growth from 100% confluent cultures. Cells were grown until they reached ~50% confluency and then transfected using JETprime transfection reagent (Polyplus #101000015) according to the manufacturer's instructions. Briefly, 150 ng of plasmid DNA was mixed with 1.5 µL JETprime reagent in 170 µL JETprime buffer (Polyplus #201000003), incubated at room temperature for 10 min, and added dropwise to the cells. The culture medium was replaced with fresh complete medium 4 h after transfection. Cells were typically harvested 24 - 48 h post-transfection.

HeLa cells were seeded onto glass coverslips in 12-well plates at ~20% confluency following overnight growth from 100% confluent cultures. Cells were grown until they reached ~50%

confluency and then transfected using JETprime transfection reagent (Polyplus #101000015) according to the manufacturer's instructions. Briefly, 100 ng of plasmid DNA was mixed with 1.0 µL JETprime reagent in 170 µL JETprime buffer (Polyplus #201000003), incubated at room temperature for 10 min, and added dropwise to the cells. The culture medium was replaced with fresh complete medium 4 h after transfection. Cells were typically harvested or imaged 24–48 h post-transfection.

Cell preparation for imaging. HeLa cells grown on glass coverslips were fixed, permeabilized, and blocked as described (49). For detection of transfected HA-tagged proteins, Alexa Fluor 488–conjugated anti-HA antibody (Cell Signaling Technology #2350; 1:100) was used. Endogenous CCDC127 was detected using anti-CCDC127 antibody (Thermo Fisher Scientific #PA5-60912; 1:250). Mitochondria were labeled with anti-TOM20 antibody (Proteintech #11802-AP; 1:500). For secondary detection, Cy3-conjugated AffiniPure anti-rabbit IgG antibody (Jackson ImmunoResearch #711-165-152; 1:1,000) was applied for 1 h at room temperature in a dark, humidified chamber. Coverslips were mounted using EverBrite™ mounting medium (Biotium #23004) and allowed to dry overnight in a dark, dry environment prior to confocal imaging.

Cell imaging. HeLa cells expressing Split-GFP SPRIZ constructs were imaged using a Zeiss Observer Z1/O7 widefield microscope equipped with a 63×/1.4 NA oil Plan-Apochromat objective. The following filter sets were used: Blue (DAPI): Ex G365, FT 395 nm, Em BP 445/50 nm; Green (GFP): Ex BP 450/50 nm, FT 480 nm, Em BP 510/50 nm; Red: Ex BP 550/25 nm, FT 570 nm, Em BP 605/70 nm.

For high-resolution imaging, samples were additionally acquired on a Zeiss LSM 880 point-scanning confocal microscope operated in Airyscan mode using a 63×/1.4 NA oil Plan-Apochromat DIC M27 objective. Airyscan detection settings were as follows: DAPI: BP 420–480 + BP 495–620, detector gain 800; Alexa Fluor 488: BP 420–480 + BP 495–620, detector gain 800; Cy3: BP 420–480 + BP 495–620, detector gain 800.

5. Transmission electron microscopy (TEM)

- TEM: sample preparation of mouse liver tissue
- TEM: sample preparation of HeLa cells
- TEM: image acquisition
- Mitochondrial morphometric EM analysis
- Mitochondrial intermembrane space EM analysis
- *Cristae* ultrastructure EM analysis
- Quantitative morphometry analysis of mitochondria-associated membranes (MAMs)

TEM: sample preparation of mouse liver tissue. Liver samples were collected from the left hepatic lobe and cut into approximately 5 mm × 5 mm × 5 mm pieces prior to fixation. Tissues were fixed by immersion in 10 mL of 4% paraformaldehyde (Electron Microscopy Sciences

#15713) and 2.5% glutaraldehyde (Electron Microscopy Sciences #16020) prepared in 0.1 M sodium cacodylate buffer pH 7.4 at room temperature. After 45 min, samples were transferred to 10 mL of the fresh fixative and incubated for an additional 5 h, followed by overnight incubation in 0.1 M sodium cacodylate buffer pH 7.4 at 4 °C.

Fixed tissue was sectioned into 50-µm-thick slices using a vibratome (Leica VT1000S). Sectioning parameters were optimized empirically, with vibration frequency set to 10 and cutting speed to 3. Sections were subjected to reduced osmium postfixation by incubation for 1 h at room temperature in the dark in a freshly prepared solution consisting of equal volumes of 3% potassium ferrocyanide ($K_4Fe(CN)_6$; BioShop #PFC232.250) in 0.1 M phosphate buffer and 4% aqueous osmium tetroxide (OsO_4; Electron Microscopy Sciences #1950) (50).

Thiocarbohydrazide (TCH; Electron Microscopy Sciences #21900) was prepared by dissolving 0.1 g TCH in 10 mL Milli-Q water, incubating at 60 °C for 1 h with gentle agitation, and filtering through a 0.22-µm syringe filter. Sections were washed five times in Milli-Q water (3 min each), incubated in filtered TCH for 20 min at room temperature in the dark, washed again five times in Milli-Q water, and exposed to a second osmium step consisting of 2% OsO_4 in Milli-Q water for 30 min at room temperature, followed by five additional washes.

Samples were dehydrated through an ice-cold graded ethanol series (30%, 50%, 70%, 80%, 90%, 100%, 100%, and 100%; 2 min each). Ethanol was replaced with propylene oxide (Sigma-Aldrich #110205-1L) for 5 min to ensure complete solvent exchange. All propylene oxide steps were performed in glass vials using glass syringes.

Durcupan ACM resin (Electron Microscopy Sciences #14040) was prepared by sequential mixing of 20 g component A, 20 g component B, 0.6 g component C, and 0.4 g component D with gentle agitation. Dehydrated sections were incubated overnight at RT in freshly prepared resin, briefly warmed at 55 °C, mounted between Aclar sheets (Electron Microscopy Sciences #50426-25), and polymerized at 55 °C for 72 h.

Embedded regions were trimmed into ~2 mm^2 blocks and mounted on resin stubs using cyanoacrylate adhesive. Ultrathin sections (~80 nm) were cut using an ultramicrotome (Leica UC6), collected on 200-mesh copper grids.

TEM: sample preparation of HeLa cells. HeLa cells were harvested by trypsinization, washed three times with phosphate-buffered saline (PBS), and pelleted at 400 × g for 10 min at room temperature, with gentle resuspension after each wash. Cells were fixed overnight at 4 °C in 1 mL of 4% paraformaldehyde and 2.5% glutaraldehyde in 0.1 M sodium cacodylate buffer pH 7.4.

Fixed cells were washed three times in 1.5 mL of 0.1 M sodium cacodylate buffer pH 7.4, embedded in 4% low-melting-point agarose, sectioned using a vibratome, and processed for reduced osmium postfixation, dehydration, resin embedding, and ultrathin sectioning, as described above for mouse liver tissue samples.

TEM: image acquisition. Grids were imaged using a JEOL JEM-1400 Flash transmission electron microscope operated at 120 kV and equipped with a LaB_6 filament and a Gatan OneView 4K digital camera. Image acquisition was performed in a blinded manner with respect to experimental condition. Low-magnification micrographs were acquired at 2,000× - 4,000×, corresponding to pixel sizes of 5.47 nm/pixel (2,000×) to 2.68 nm/pixel (4,000×). High-magnification micrographs were acquired at 15,000× - 40,000×, corresponding to pixel sizes of 0.715 nm/pixel (15,000×) to 0.266 nm/pixel (40,000×).

Mitochondrial morphometric EM analysis. To quantify mitochondrial number and area in mouse liver EM micrographs, mitochondria imaged at low-magnification were segmented using napari (v0.4.18) with the empanada extension (v1.2). The MitoNet_v1 model was run with an image downsampling factor of 4, a segmentation confidence threshold of 0.50, and a center confidence threshold of 0.10, as these settings produced outputs that were most practical for downstream manual curation. All segmentation outputs were manually inspected and curated to ensure accurate mitochondrial boundary delineation to generate binary masks.

Binary masks were generated in which each particle corresponded to a single mitochondrion. Mitochondrial area and perimeter were quantified using the Analyze Particles function in Fiji/ImageJ (v1.54p). For mitochondrial abundance, the number of segmented mitochondria was determined for each image and normalized to 225 μm^2 by scaling to the imaged field area (computed from image dimensions and pixel size).

Mitochondrial intermembrane space EM analysis. For intermembrane space (IMS) quantification high-magnification TEM images (15,000× - 40,000×) were preprocessed in Fiji/ImageJ by conversion to 8-bit grayscale, application of a mean filter (radius 4 pixels), and Otsu thresholding. When automated thresholding failed to separate membrane-associated signal from background, threshold values were minimally adjusted using a uniform visual criterion applied across images with reference to the original grayscale data.

Outer and inner mitochondrial membranes were manually traced along membrane contours. Mitochondria exhibiting non-orthogonal sectioning were included when overall membrane trajectories could be reliably determined. The IMS area was calculated as the unmasked pixel area confined between traced membrane boundaries. Outer membrane tracing was additionally used to calculate mitochondrial perimeter. IMS measurements were normalized to mitochondrial perimeter and reported as the IMS area–to–perimeter ratio (fig. S6D).

TEM image acquisition and subsequent IMS analysis were performed blinded to experimental condition. The investigator acquiring the images and quantifying IMS morphology was not aware of the genotype or treatment group of the samples being analyzed. For quantification, images were acquired only from high-quality mitochondrial profiles with clearly preserved membrane contrast and reliably assignable OMM and IMM boundaries. This criterion was applied uniformly and blindly across all experimental groups, such that no ambiguous cases requiring subjective group-dependent assignment were encountered during IMS quantification. Because mitochondria were selected on the basis of image quality and membrane clarity rather than

experimental phenotype, this acquisition strategy also reduced operator bias during sampling and introduced an element of randomization.

The IMS measurement strategy was designed to minimize subjective manual estimation of IMS area. Manual tracing was used only to define the outer and inner mitochondrial membrane boundaries, thereby delimiting the region of interest within which segmented IMS pixels were quantified. This semi-automated workflow reduced operator-dependent estimation of IMS area and ensured that quantification relied primarily on segmented pixel density rather than manual freehand drawing of the IMS itself.

***Cristae* ultrastructure EM analysis.** *Cristae* were analyzed using the same high-magnification TEM images which were used for the IMS quantification. *Cristae* were manually traced in Fiji/ImageJ. Ambiguous or diffuse *cristae* were excluded from analysis.

Cristae were quantified as individual regions of interest (ROIs), from which *cristae* area and aspect ratio were measured in Fiji/ImageJ. *Cristae* density was calculated by normalizing total *cristae* area per mitochondrial area, obtained from outer membrane tracing during IMS quantification.

Quantitative morphometry analysis of mitochondria-associated membranes (MAMs). High-magnification TEM images of HeLa cells were preprocessed in Fiji/ImageJ (v1.54p) via Brightness/Contrast tool to enhance image contrast and facilitate identification of MAMs. The outer mitochondrial membrane (OMM) and ER membrane regions located within ~25 nm of the OMM were manually traced. The resulting tracings were used to quantify the number of MAMs per cell, the percentage of mitochondria containing 0, 1, or ≥2 MAM contacts, individual MAM lengths, and the percentage of mitochondrial perimeter occupied by MAMs, calculated as the cumulative MAM length normalized to the mitochondrial perimeter.

6. Molecular dynamics simulation

- Atomistic Molecular Dynamics Simulations
- Coarse-grained simulations of SPRIZ C-terminal domain
- Coarse-grained simulation of SPRIZ homodimer tethering OMM and IMM
- Molecular dynamics trajectory analysis

Atomistic Molecular Dynamics Simulations. AlphaFold3–predicted homodimeric structures of SPRIZ (UniProt Q96BQ5) and MARTINI (Fig. 4A) were prepared using CHARMM-GUI (42), with protonation states assigned according to CHARMM-GUI recommendations. Protein structures were embedded using the PPM 2.0 server into a 120 Å × 120 Å heterogeneous lipid bilayer representing the generic outer mitochondrial membrane composition with asymmetric leaflets: POPC:POPE:POPI:POPS:CHOL at 54:20:22:3:10 and 54:38:4:1:10 in the upper and lower leaflets, respectively, as defined in the OPM (Orientations of Proteins in Membranes) database (43).

The system was solvated with TIP3P water molecules and ionized to a final concentration of 0.15 M NaCl. Hydrogen mass repartitioning (51) was applied to enable a 4-fs integration time step.

All simulations were performed using NAMD 3 (44) with the CHARMM36 and CHARMM36m force fields (45). A three-stage relaxation protocol was applied as described in the NAMD tutorial for membrane protein simulations. First, the system underwent 7,500 steps of energy minimization followed by 10 ns of equilibration with restraints focused on the lipid tails. Second, an additional 7,500 minimization steps were performed, followed by 10 ns of equilibration with restraints applied to the protein while allowing membrane lipids and solvent to relax. Third, the system was minimized for 7,500 steps and equilibrated for 50 ns with backbone restraints applied to the protein.

Following equilibration, 3 independent production simulations were performed for 500 ns for each system.

In production simulations, nonbonded interactions were calculated using Lennard-Jones potentials for van der Waals forces and Coulombic interactions for electrostatics with a 12 Å cutoff and a switching function applied at 10 Å. Long-range electrostatics were treated using the particle mesh Ewald (PME) method (52) with a grid spacing of 1 Å. Water molecules were constrained using the SETTLE algorithm (53), and bonds involving hydrogen atoms were constrained using the SHAKE (54) and RATTLE (55) algorithms. Temperature was maintained at 310 K using a Langevin thermostat (56), and pressure was controlled at 1 atm using a modified Nosé–Hoover Langevin piston method (57).

Coarse-grained simulations of SPRIZ C-terminal domain. Coarse-grained (CG) molecular dynamics simulations were performed using wild-type and loops mutant C-terminal domain of SPRIZ (amino acids 115–260) generated by truncation of the full-length AlphaFold3–predicted SPRIZ homodimer model. Truncation of the full-length SPRIZ homodimer and introduction of loops mutations (L176D, F177D, L178D, V222D, and W223D) were performed in PyMOL.

Atomistic structures were converted to CG representations using the martinize2.py (58) with the MARTINI 3.0 force field (38), including elastic network restraints (500 kJ mol^{-1} nm^{-2}) between all chains in a structure. A 130 × 130 Å heterogeneous lipid bilayer was constructed using the insane.py script based on the generic inner mitochondrial membrane composition reported by the OPM server (43), with lipid distributions of POPC:POPE:POPS:POPI:CDL2, 58:37:3:5:11. The system was solvated and ionized to a final concentration of 0.15 M NaCl.

Wild-type and loops mutant SPRIZ C-terminal domain homodimer models were positioned identically at 15 Å above the membrane surface prior to equilibration.

CG simulations were performed using GROMACS 2025.3 (46). Systems underwent an initial soft-core energy minimization for 40,000 steps using a lambda value of 0.01, followed by steepest descent minimization for 100,000 steps or until convergence to 10 kJ mol^{-1} nm^{-1}. Subsequently, NPT equilibration was conducted for 150 ns.

Production simulations were carried out for 3 µs using a 20-fs time step. Temperature was maintained at 300 K using the velocity-rescale (V-rescale) thermostat, and pressure was controlled at 1 bar using a semi-isotropic Parrinello–Rahman barostat (59). Electrostatic interactions were treated using the reaction field method with a cutoff of 1.1 nm, while van der Waals interactions were truncated at 1.1 nm using the potential-shift Verlet scheme. The Verlet cutoff scheme was applied with neighbor lists updated every 20 steps. Ten independent replicas were performed per condition, yielding a total simulated time of 30 µs per condition.

Coarse-grained simulations of SPRIZ homodimer tethering OMM and IMM. The AlphaFold3–predicted wild type and loops mutant SPRIZ homodimer structures were converted to CG representations using martinize2.py (58) with the MARTINI 3.0 force field (38), including elastic network restraints (500 kJ mol^{-1} nm^{-2}) between all chains in a structure. To facilitate solvent exchange across the outer mitochondrial membrane and ensure system stability (47), two copies of the voltage-dependent anion channel protein (VDAC; UniProt: P21796) were also converted to CG representations and incorporated into the OMM-like membrane as described (47). Both SPRIZ and VDAC proteins were oriented relative to the membrane using the PPM 3.0 web server prior to system assembly.

Protein orientations were optimized using the Bentopy packing workflow (60) based on moment-of-inertia–guided rearrangement prior to membrane insertion. The 120 × 120 Å OMM-like membrane was constructed using insane.py as a heterogeneous lipid bilayer with asymmetric leaflet distributions based on the generic mitochondrial outer membrane composition according to the OPM database: POPC:POPE:POPI:POPS:CHOL; 54:20:22:3:10, POPC:POPE:POPI:POPS:CHOL; 54:38:4:1:10 in upper and lower leaflets, respectively. Lipids occupying VDAC pore regions introduced by ring-based membrane packing were manually removed to prevent steric artifacts using PyMOL (47).

Following OMM-like membrane assembly, a second membrane representing the inner mitochondrial membrane (IMM) was added using insane.py with lipid distributions according to the OPM database: POPC:POPE:POPS:POPI:CDL2, 58:37:3:5:11. The IMM was positioned approximately 15 Å from the SPRIZ C-terminal domain. The resulting dual-membrane system was solvated and ionized to a final concentration of 0.15 M NaCl. System topologies were generated by adapting procedures described in a recent approach (47); this involved manually preserving lipid ordering, and creating custom index groups defining the proteins, OMM, IMM, and solvent/ions.

CG simulations were performed using GROMACS 2025.3 (46) under the same simulation conditions, minimization, and equilibration parameters as described for the coarse-grained simulations of the SPRIZ C-terminal domain. Following equilibration, production simulations consisted of three independent runs of 5 µs each for both wild-type and loops mutant systems, yielding a total simulation time of 15 µs per condition.

Molecular dynamics trajectory analysis. Trajectory analyses were performed using the MDAnalysis Python package (61). Angular distributions from atomistic simulations and distance-

based analyses from coarse-grained simulations of the SPRIZ C-terminal domain were computed using MDAnalysis.

Root-mean-square deviation (RMSD) was calculated for all simulations following correction for periodic boundary conditions and recentering around the protein. For coarse-grained trajectories, recentering was performed using GROMACS tools, while atomistic trajectories were processed using VMD. Final trajectory renderings and molecular visualization movies were generated using Blender Molecular Nodes (62).

For video rendering in Blender using Molecular Nodes, coarse-grained simulations were smoothed with a window of 100 frames and rendered at 60 frames per second, using every 16th frame for the SPRIZ C-terminal conical domain and every 24th frame for the full-length SPRIZ tethering OMM and IMM in GROMACS. The system was recentered on the protein prior to rendering.

7. Other procedures

- Isolation of mouse liver wrappER-associated mitochondria- and mitochondria-enriched fractions
- AAV8-mediated gene delivery
- Proteomic analysis
- Expression plasmid construction and purification
- SDS-PAGE and immunoblotting
- *In vitro* transcription and translation
- SPRIZ (CCDC127) evolutionary analysis. Identification of SPRIZ homologs and phylogenetic analysis
- Electron tomography acquisition and quantification
- Cryo-ET: Tomogram 3D reconstruction
- Radiotracer assay of IMS-accessible volume

Isolation of mouse liver wrappER-associated mitochondria- and mitochondria-enriched fractions. Mouse liver WrappER-associated mitochondrial (WAM) fractions were isolated according to a previously published protocol (32) and the pellet was resuspended in 1 mL of EB10 buffer (250 mM sucrose, 10 mM Tris-HCl pH 7.4, 1 mM $MgCl_2$, 0.1 mM EGTA). Mitochondria-enriched fractions were subsequently obtained by differential centrifugation of WAM fractions in 25% Percoll at 95,000 × g for 35 min.

AAV8-mediated gene delivery. Codon-optimized SPRIZ, SPRIZ loops mutant or MARTINI sequences were cloned into the pAAV.TBG.PI.Null.bGH expression vector (Addgene plasmid #105536) under control of the liver-specific thyroxine-binding globulin (TBG) promoter. Recombinant AAV8 particles were produced by Franklin Biolabs and purified by two rounds of

CsCl gradient ultracentrifugation, followed by desalting and titer determination (final titer > 6 × 10^{13} genome copies [GC] mL^{-1}).

Six-month-old mice were administered AAV8 vectors by intraperitoneal injection at a dose of 1.8 × 10^{11} GC diluted in 100 µL of sterile phosphate-buffered saline (PBS). Experimental analyses were performed 21 days post-injection.

Proteomic analysis. Mitochondria-enriched fractions were obtained from livers of 8-week-old mice. Control samples were derived from *Ccdc127*$^{+/+}$ mice, and knockout samples from *Ccdc127*$^{-/-}$ mice. The mitochondrial pellet was resuspended in 10 mM Tris-HCl buffer containing 10 mM EGTA, 250 mM sucrose. Sodium deoxycholate was added to a final concentration of 1% (w/v), followed by vortexing and sonication for 20 s using a probe sonicator (1 s on, 1 s off cycle). Proteins were precipitated by addition of 1 mL ice-cold acetone and incubation overnight at −20 °C. Samples were centrifuged at 10,000 × g for 15 min at 4 °C, after which supernatants were removed and protein pellets were resuspended in 50 µL of 50 mM ammonium bicarbonate. Due to incomplete pellet solubilization, only the soluble fraction was used for downstream analysis. Protein concentration was determined using a rapid BCA assay. Proteins were reduced with dithiothreitol (DTT) and alkylated with iodoacetamide, followed by overnight digestion with trypsin at a 1:50 enzyme-to-protein ratio at 37 °C. Resulting peptides were purified using C18 StageTips, dried by vacuum centrifugation, and resuspended in LC loading solvent. Approximately 0.5 µg of peptides was injected per analysis.

Peptide samples were analyzed using a Vanquish Neo UHPLC system coupled to an Orbitrap Astral mass spectrometer via an EASY-Spray ion source. Peptides were separated on an 8 cm × 150 µm inner diameter capillary column (1.7 µm particle size; IonOpticks Aurora Rapid XT) using a linear gradient of 5–34% solvent B over 15 min (solvent B: 100% acetonitrile in 0.1% formic acid) at a flow rate of 1 µL min^{-1}, followed by a column wash at 85% solvent B for 2 min at 2 µL min^{-1}, resulting in a total runtime of 17 min. The column was maintained at 50 °C.

Spray voltage was set to 1,800 V and capillary temperature to 280 °C. Data were acquired in positive ion mode using profile acquisition with EASY-IC internal lock mass calibration.

Full MS scans were acquired in the Orbitrap at a resolution of 180,000 over a mass range of 380–980 m/z, with an AGC target of 500% and a maximum injection time of 5 ms. DIA acquisition consisted of 150 non-overlapping isolation windows of 4 m/z covering the same precursor range, with fragment ion detection in the Astral analyzer using an AGC target of 500% and a maximum injection time of 3.5 ms. Normalized higher-energy collisional dissociation (HCD) collision energy was set to 25%.

Raw DIA files were processed using DIA-NN (v2.0). A predicted spectral library was generated by in silico digestion of the *Mus musculus* UniProt reference proteome (Proteome ID UP000000589; 54,747 entries; June 2025 release), with deep learning–based spectral prediction enabled. Trypsin/P was specified as the digestion enzyme with a maximum of two missed cleavages allowed. Carbamidomethylation of cysteine was set as a fixed modification, while methionine

oxidation and N-terminal methionine excision were included as variable modifications (up to two per peptide).

Peptides ranging from 7 to 30 amino acids and precursor charge states from 2+ to 4+ within the 380–980 m/z range were considered. Fragment ion mass range was set to 100–2000 m/z. Match-between-runs (MBR) was enabled, protein inference was performed at the protein name level, and all other parameters were kept at default settings.

Protein digestion, mass spectrometry analyses and protein identification were performed by the Proteomics Platform of the CHU de Québec-Université Laval Research Center, Québec City, Canada.

Expression plasmid construction and purification. Plasmids for human cell transfection were synthesized by Twist Bioscience. Coding sequences were cloned into a BetaGlobin WPRE Neo expression vector under the control of the CMV promoter. Plasmid DNA was purified using the PureLink HiPure Plasmid MaxiPrep kit (Invitrogen, #K210007) according to the manufacturer's instructions and quantified by absorbance using a NanoDrop spectrophotometer (Thermo Scientific). Plasmid preparation and quantification were performed at the Genomic Engineering and Molecular Biology (GEMb) Core Facility, Faculty of Medicine, University of Ottawa.

SDS-PAGE and immunoblotting. Total protein concentration was determined using the Bradford protein assay. Protein samples were resolved by SDS–PAGE using precast gels according to the manufacturer's instructions (Thermo Fisher Scientific). Proteins were transferred to PVDF membranes (Immobilon, Millipore; 0.45 µm pore size) for 40 min at 100 V in transfer buffer containing 20% methanol, 320 mM glycine, and 20 mM Tris-base (pH 8.4).

Membranes were blocked for 60 min at room temperature with 8% bovine serum albumin (BSA) in Tris-buffered saline containing 0.1% Tween-20 (TBS-T). Primary antibodies were incubated overnight at 4 °C in 5% BSA in TBS-T. The primary antibodies used in this study were the following: anti-CCDC127 (1:1000; ThermoFisher #PA5-60912), anti-AIFM1 (1:25000; Cell Signaling #5318S), anti-MnSOD (1:25000; Enzo Life Sciences #ADI-SOD-110-D), anti-Calnexin (1:20000; Biosciences #SDC-108B), anti-OPA1 (1:50000; BD Biosciences #612607), anti-Synj2bp (1:20000; Sigma-Aldrich #HA000866), anti-Calreticulin (1:60000; Cell Signaling #2891S), anti-TOM20 (1:40000; Proteintech #11802 -1-AP), anti-HA (1:15000; Cell Signaling #3724S), anti-FLAG (1:10000; Cell Signaling #8146S), anti-MICU1 (1:500; Sigma Aldrich#HPA034779), anti-MICU2 (1:500; Abcam#ab101465), anti-MCU (1:1000; Cell Signaling#14997), and anti-EMRE (1:1000; Bethyl#A300-BL19208).

After washing, membranes were incubated with HRP-conjugated secondary antibodies diluted in 5% BSA in TBS-T for 1 h at room temperature. The secondary antibodies used were anti-mouse IgG (1:5000; Cell Signaling #7076S) and anti-Rabbit IgG (1:5000; Cell Signaling #7074S).

Protein bands were visualized by chemiluminescence using the SuperSignal ELISA Femto substrate (Thermo Fisher Scientific) and imaged with a VersaDoc 3000 CCD imaging system (Bio-Rad Laboratories).

***In vitro* transcription and translation** were performed using the PURExpress *in vitro* Protein Synthesis Kit (New England Biolabs, #E6800) according to the manufacturer's instructions with minor modifications. Reaction mixtures were assembled on ice in a final volume of 25 µL. 10 µL of Solution A, 7.5 µL of Solution B, 7.25 µL of nuclease-free water, and 0.25 µL of plasmid DNA were combined. Plasmid DNA was used at a concentration of 1 mg/mL, corresponding to 0.25 µg DNA per reaction.

Prior to assembly, all reagents were thawed on ice. Solution A was mixed thoroughly, whereas Solution B was mixed gently by inversion. Reaction components were combined, gently mixed, and briefly pulse-centrifuged to collect the contents at the bottom of the tube. Reactions were incubated at 37 °C for 2 h. Reactions were terminated by placing tubes on ice, followed by the addition of 25 µL of RIPA buffer. Samples were stored at −20 °C until further analysis by SDS–PAGE and immunoblotting.

SPRIZ (CCDC127) evolutionary analysis. **Identification of SPRIZ homologs and phylogenetic analysis**. Eukaryotic genome assemblies were retrieved from NCBI and filtered to include assemblies annotated as RefSeq "reference genome" and with an assembly level of either "Complete Genome" or "Chromosome." This yielded 7,025 assemblies. To reduce redundancy, one representative assembly per eukaryotic family was selected based on the largest number of annotated proteins, resulting in a final comparative dataset of 605 assemblies representing 31 phyla.

Initial vertebrate and invertebrate SPRIZ homologs were identified from the NCBI clustered non-redundant protein database using Web BLASTP. Vertebrate and invertebrate SPRIZ sequences were aligned separately using FAMSA. These two alignments were then used as profile queries for MMseqs2 searches against the annotated protein sets from the selected assemblies, using a sensitivity value of 7.5 and an e-value threshold of 1×10^{-6}. This search identified 711 distinct candidate SPRIZ homologs in 292 assemblies. Homologs were detected in all sampled Deuterostomia except Tunicata, as well as in one Bryozoa assembly and both available Nemertea assemblies.

To improve taxonomic coverage outside crown vertebrates, the same SPRIZ profiles were also used to search the NCBI clustered non-redundant protein database with MMseqs2 using the same parameters. Non-Gnathostomata hits were clustered at 90% sequence identity, and distinct hits were added to the dataset. This additional search recovered 19 proteins from 15 assemblies, including additional homologs from non-gnathostome deuterostomes and isolated protostome species.

Because some assemblies contained multiple predicted SPRIZ paralogs or isoforms, SPRIZ homologs were clustered within each assembly at 90% sequence identity, and one representative

sequence was selected from each cluster. This produced an initial representative set of 363 SPRIZ homologs. Sequences that were poorly aligned, partial, or likely to represent misassembled or misannotated proteins were removed after alignment inspection, yielding a final dataset of 352 SPRIZ homologs.

Final protein sequences were aligned with FAMSA. Alignment sites with a weighted gap fraction greater than two-thirds and homogeneity below 0.01 were removed prior to phylogenetic reconstruction. A maximum-likelihood phylogenetic tree was inferred using IQ-TREE. The best-fitting substitution model was selected with IQ-TREE ModelFinder and corresponded to Q.mammal+F+R6. Branch support was estimated using the aBayes method implemented in IQ-TREE.

Electron tomography acquisition and quantification. For three-dimensional electron tomography, 150 nm thick sections were collected on formvar-carbon coated slot grids and stained with uranyl acetate and Sato's lead. A series of double-tilted images (±60°) was acquired using a Talos L120C transmission electron microscope operated at 120 kV. Images were acquired at 22,000× magnification, corresponding to a pixel size of 0.644 nm, using Tomography 5 software (FEI, Thermo Fisher Scientific). Tilt series were processed in IMOD. Following alignment, tomograms were reconstructed by weighted back-projection and the two reconstructed volumes were binned and combined to generate the final dual-axis tomograms (pixel size 1.287 nm).

3D reconstruction of the outer and inner mitochondrial membranes (OMM and IMM) was performed using 3dmod (v5.1.11). The OMM and IMM were manually traced as separate objects using the drawing tools. The resulting contour sets were converted into surface meshes using the Meshing tool in the Model View Object Editing window. The reconstructed meshes were exported as OBJ files and imported into Blender, where Laplacian smoothing was applied prior to final image rendering.

OMM–IMM distances were quantified from 3D electron tomography reconstructions using segmented outer and inner mitochondrial membranes. Segmented membrane objects were exported from IMOD and processed using a custom Python script. For each voxel assigned to the OMM, the nearest IMM voxel was identified in three-dimensional space using a nearest-neighbor search, and the corresponding Euclidean distance was calculated. Distances were compiled for each reconstructed mitochondrion and used to generate distance distributions and summary statistics. Where indicated, representative OMM-to-IMM nearest-neighbor vectors were visualized on the segmented reconstructions.

Cryo-ET: Tomogram 3D reconstruction. 3D visualization of the cryo-ET tomogram combined utilization of UCSF ChimeraX (v1.12) and Blender (v5.0.1). In ChimeraX, the Volume Viewer tool was used to invert volume intensity values, converting electron-dense features from negative to positive density values. A surface representation was then generated from the positive density values using the Volume Viewer surface settings (style = surface, step = 1, level = −95). Disconnected isosurface components were removed using the Hide Dust function (size threshold

= 1000). The resulting surface mesh was exported as an OBJ file and imported into Blender, where Laplacian smoothing was applied prior to final image rendering.

Radiotracer assay of IMS-accessible volume. The mitochondrial intermembrane space-accessible volume was estimated using a radiotracer-based time-course assay with ^{14}C-mannitol (American Radiolabeled Chemicals, #ARC3338) and ^{14}C-dextran (American Radiolabeled Chemicals, #ARC0127A). ^{14}C-mannitol was used as a small molecule that can access the mitochondrial intermembrane space, whereas 10 kDa ^{14}C-dextran was used as an exclusion control to estimate residual extramitochondrial buffer carried over after washing.

Freshly isolated mitochondria were resuspended in assay buffer containing 250 mM sucrose, 10 mM Tris-HCl pH 7.4, and 0.1 mM EGTA, and divided into two equal aliquots for parallel incubation with either ^{14}C-mannitol or ^{14}C-dextran. Each mitochondrial suspension was mixed 1:1 with the corresponding radiotracer solution to a final volume of 200 µL and a final activity of 1 µCi/mL. Samples were incubated for 5, 10, 15, 20, or 30 min at RT with constant shaking.

At each time point, mitochondria were pelleted by centrifugation at 10,000 × g for 5 min. The first supernatant was collected, and the mitochondrial pellet was washed once with 500 µL assay buffer, followed by a second centrifugation at 10,000 × g for 5 min. The wash supernatant was collected, and the final mitochondrial pellet was resuspended in 100 µL of assay buffer. Radioactivity in the final mitochondrial pellet was quantified using a Tri-Carb 4910TR Liquid Scintillation Counter. The same mitochondrial preparation used for radiotracer measurement was also used for protein quantification by BCA assay, allowing normalization of radiotracer signal to mitochondrial protein content.

Supplementary Figure 1

A

Maximum-likelihood phylogeny of SPRIZ homologs across Bilateria

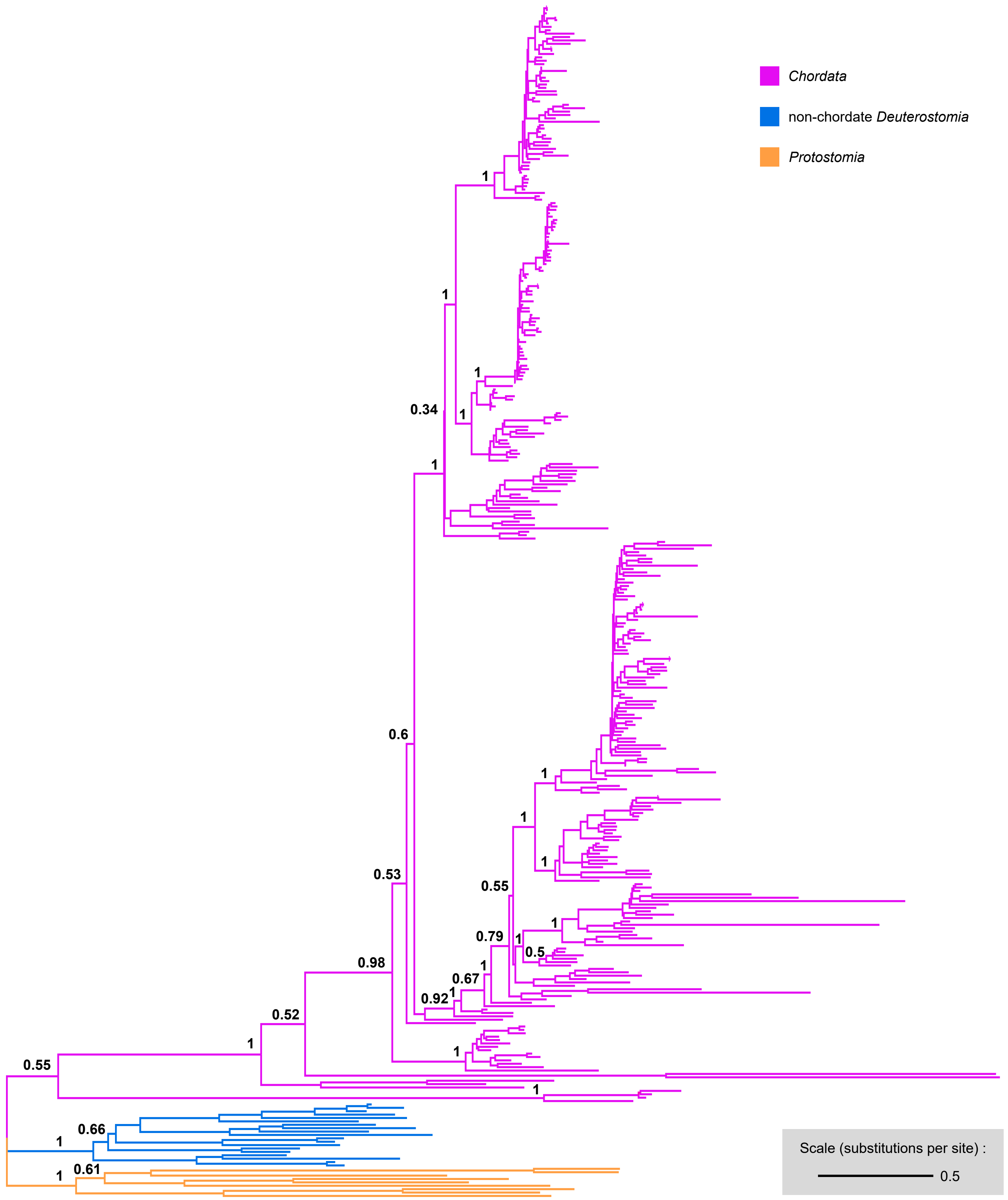

Supplementary Figure 1

B

Maximum-likelihood phylogeny of SPRIZ homologs across *Chordata*

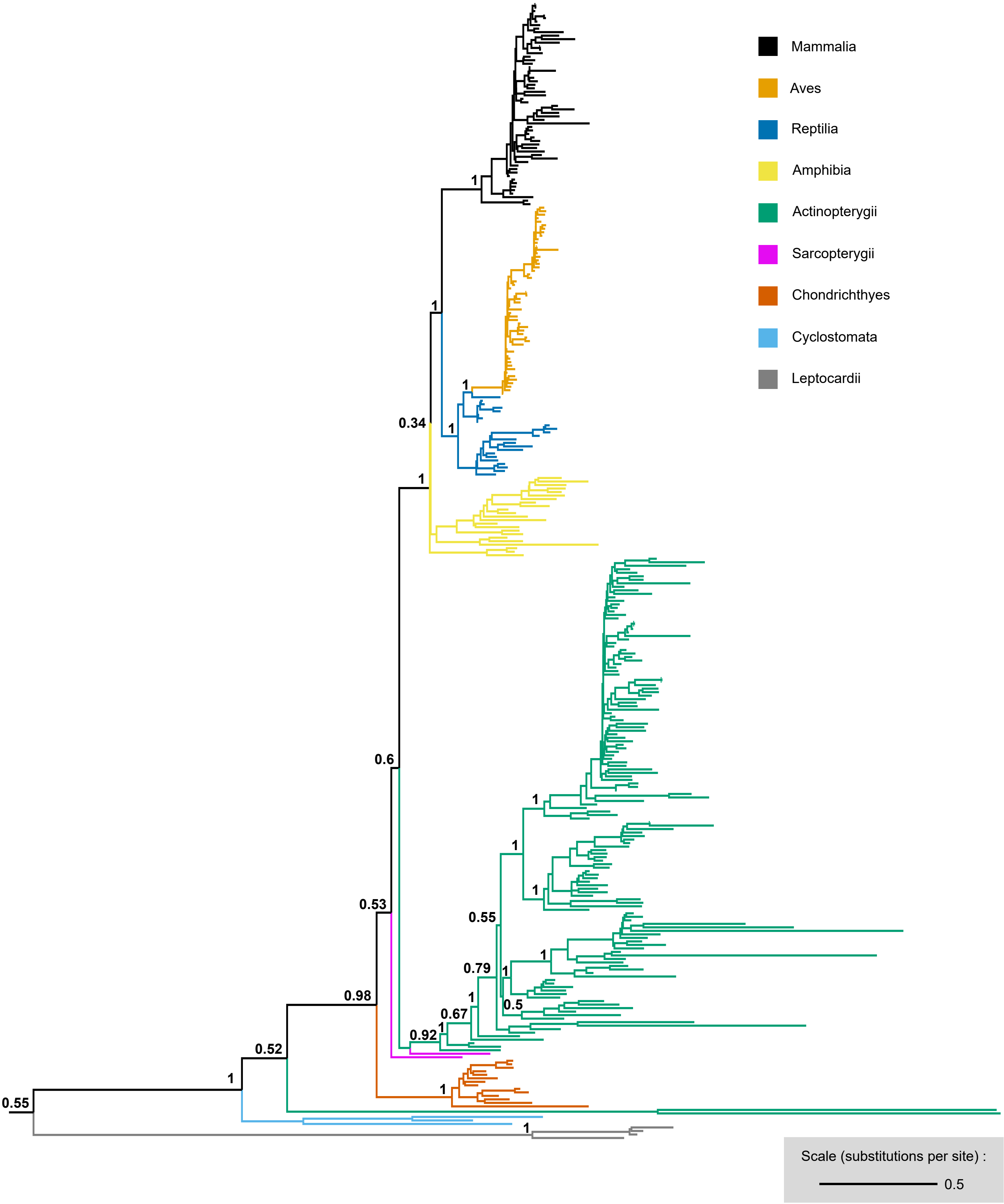

Supplementary Figure 1

C Maximum-likelihood phylogeny of SPRIZ homologs across non-chordate *Deuterostomia* and *Protostomia*

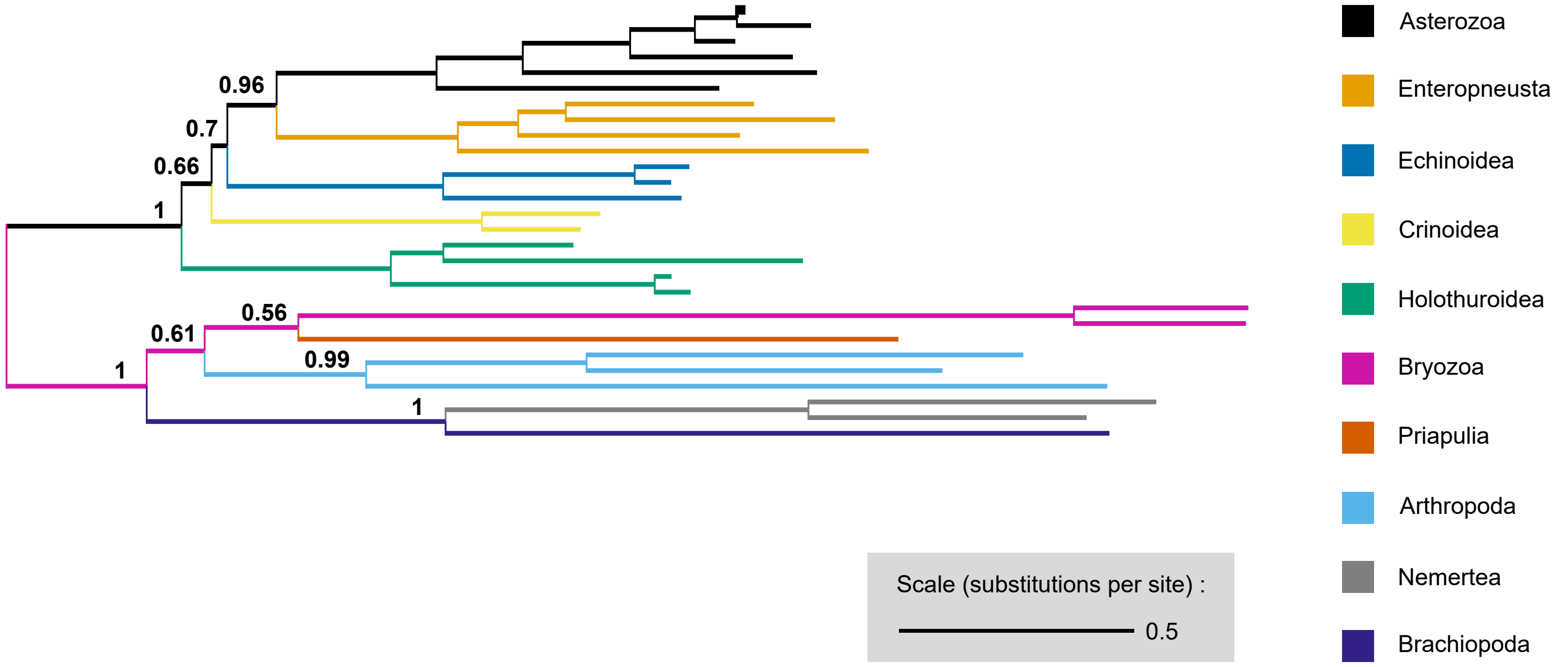

Fig. S1. Phylogenetic analysis of SPRIZ.

(A) Maximum-likelihood phylogeny of SPRIZ homologs across Bilateria. Branches are colored according to major bilaterian groups: Chordata (magenta), non-chordate Deuterostomia (blue), and Protostomia (orange). Node values indicate bootstrap branch support. Scale bar indicates substitutions per site. **(B)** Maximum-likelihood phylogeny of SPRIZ homologs across Chordata. Branches are colored according to major chordate groups: Mammalia (black), Aves (orange), Reptilia (blue), Amphibia (yellow), Actinopterygii (green), Sarcopterygii (magenta), Chondrichthyes (vermillion), Cyclostomata (sky blue), and Leptocardii (gray). Node values indicate bootstrap branch support. Scale bar indicates substitutions per site. **(C)** Maximum-likelihood phylogeny of SPRIZ homologs across non-chordate Deuterostomia and Protostomia. Branches are colored according to taxonomic group: Asterozoa (black), Enteropneusta (orange), Echinoidea (blue), Crinoidea (yellow), Holothuroidea (green), Bryozoa (purple), Priapulia (vermillion), Arthropoda (sky blue), Nemertea (gray), and Brachiopoda (dark navy/indigo). Node values indicate bootstrap branch support. Scale bar indicates substitutions per site.

Supplementary Figure 2

Comparative bioinformatics of SPRIZ shows vertebrate-specific preservation of coiled-coil rod length and C-terminal IMM-grafting loops absent from invertebrate orthologs

A SPRIZ (CCDC127) protein evolutionary conservation in vertebrates (consensus logo)

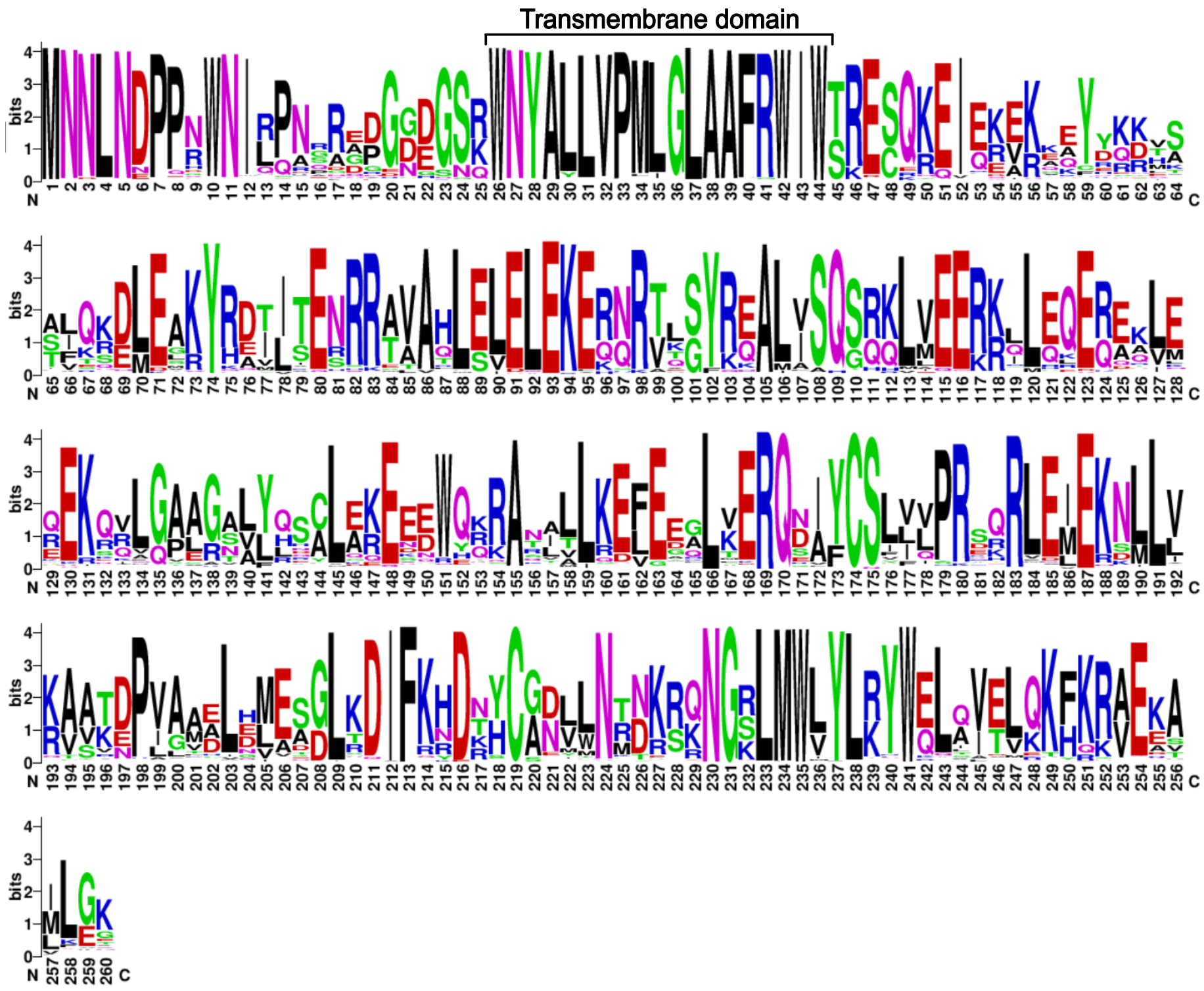

B AlphaFold3 models of horseshoe crab and mouse SPRIZ (CCDC127) orthologs

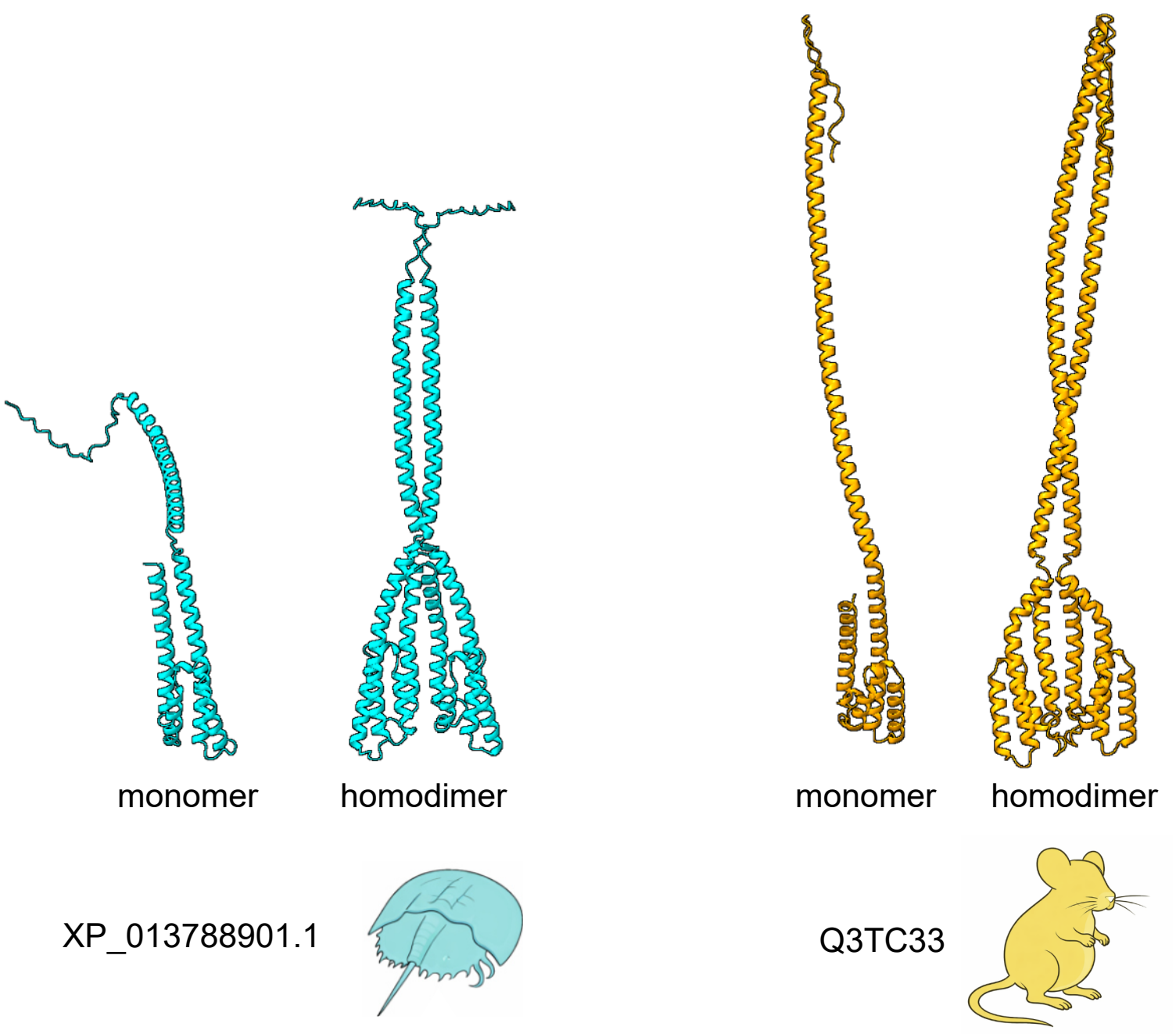

Supplementary Figure 2

Comparative bioinformatics of SPRIZ shows vertebrate-specific preservation of coiled-coil rod length and C-terminal IMM-grafting loops absent from invertebrate orthologs

C Pairwise protein sequence alignment of mouse and horseshoe crab SPRIZ (CCDC127) orthologs

```
Mouse              ---------MNNLNDPPNWNIRPNARADGGDGS-KWNYALLVPM---LGLAAFRWIWSRESQKEIEKARKAYHQRTAAFQQDLEAKYHAVISEHRRAVAQLSLELEKEQNRTSSFREALI   107
Horseshoe_crab     MDKSSKGFGWNDLNIPPTST------GPRDEKSSLKHIGVLFNLATPIALGYVYWKCQQEKTK----LKTQHSEEVQAFKKHLESV----ASQQ-------------GYVKASTFVST--    91
                             *:** **. .     .  .: *   : .:*. :  :.*. . *  .:*. *     :. : :.. **::.**:      *::              ::*:* .:

Mouse              SQGRKLAEEKKLLEQERAQIKQEKSRLQPLRNVYLSCLQEEDDWQRRAQHVLKEVGEALEERQNIYCSLIIPRSARLE--LEKSLLVRTSVDPVAADLEMAAGLSDIFKHD-KHCGDVWN   224
Horseshoe_crab     ---------------------------------IDEALREEENKSKEMLEELHKVEHQIKSILDIRCSRINPWKTRERREKQERDILKNSLTGILAPLEMYDGLQHIFHYDHLHCGGGFH   178
                                                  ..*:**:: .:.  . *::* . ::.  :* ** * * .:* .   ::  :::.*:  : * ***  **..**::*  ***. ::

Mouse              TNKRQNGKLMWMYLKYWELLVELKKFKKVEKVILEK-----   260     Q3TC33
Horseshoe_crab     K----NGQLYRVWIIHWRSLIIAHRDRAIKQQIEKLKATHG   215     XP_013788901.1
                   .    **:*  ::: :*. *:  :: : ::: * :
```

D Multiple sequence alignment of representative vertebrate and invertebrate SPRIZ (CCDC127) orthologs

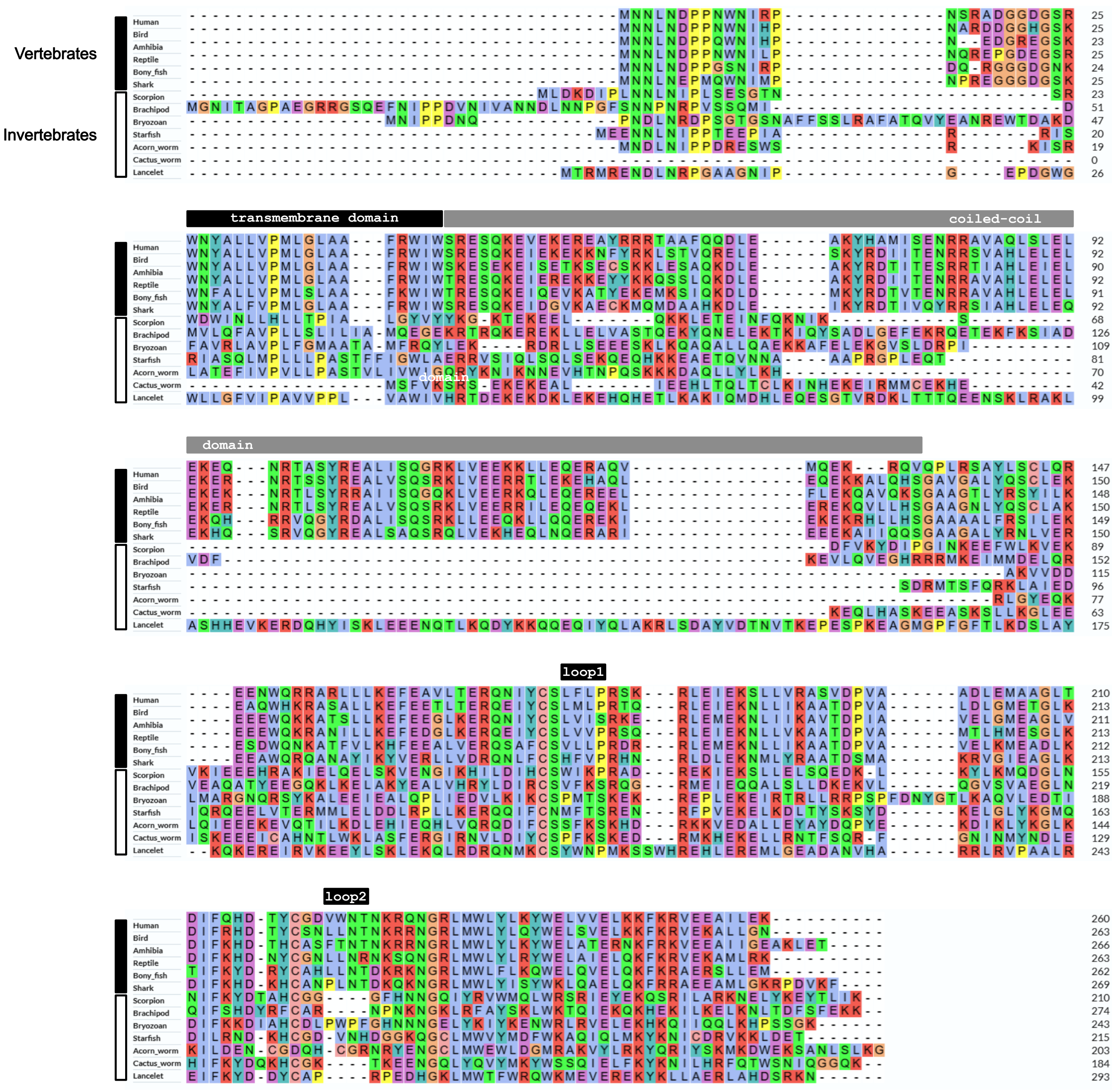

Fig. S2. Comparative bioinformatics of SPRIZ shows vertebrate-specific preservation of coiled-coil rod length and C-terminal IMM-grafting loops absent from invertebrate orthologs.

(A) Consensus sequence logo of vertebrate SPRIZ (CCDC127) orthologs, generated from multiple sequence alignment of near-universally conserved vertebrate homologs. The logo highlights highly conserved residues across the full protein length (~260 amino acids), with the N-terminal transmembrane domain bracketed. Bit height indicates conservation strength at each position; colors follow standard physicochemical grouping (e.g., polar green, basic blue, acidic red, hydrophobic black/yellow). The logo demonstrates deep primary-sequence conservation over >500 million years of vertebrate evolution. **(B)** AlphaFold3-predicted structural models of SPRIZ orthologs from distant bilaterian species: horseshoe crab (*Limulus polyphemus*, XP_013788901.1) and mouse (Q3TC33). Monomer (left) and homodimer (right) conformations are shown for each, illustrating the conserved elongated coiled-coil architecture that supports homodimerization and rigid rod-like projection. **(C)** Pairwise sequence alignment of SPRIZ (CCDC127) orthologs from mouse (*Mus musculus*, Q3TC33) and horseshoe crab (*Limulus polyphemus*, XP_013788901.1). The alignment is shown in blocks with Clustal-style annotation below each position: (*) indicates identical residues, (:) indicates strongly similar residues (high-scoring substitution, e.g., both hydrophobic or both positively charged), (.) indicates weakly similar residues (lower-scoring but related substitution), (-) indicate no similarity or gaps introduced to optimize alignment of conserved regions. The alignment highlights moderate overall sequence conservation despite the deep evolutionary divergence (~550–600 million years since the protostome–deuterostome split), with scattered patches of identity and similarity, particularly in regions corresponding to the coiled-coil domain and parts of the C-terminus. Numerous gaps reflect lineage-specific insertions and deletions. Residue numbers are indicated at the end of each line for both sequences. This limited sequence similarity contrasts with the high structural conservation observed in the AlphaFold3 models (panel B), illustrating preservation of 3D architecture and functional properties over primary sequence. **(D)** Multiple sequence alignment of representative vertebrate and invertebrate SPRIZ (CCDC127) orthologs. One representative sequence was selected from each major invertebrate clade and from the vertebrate clade. Residues are colored according to the Clustal color scheme. Predicted structural vertebrate-conserved features are indicated above the alignment, including the N-terminal transmembrane domain, the extended coiled-coil region, and the two loop regions (loop-1 and loop-2) that coordinate IMM insertion. The alignment highlights strong conservation of the transmembrane domain and coiled-coil core across vertebrates, with greater sequence divergence in the coiled-coil and loop regions among invertebrates.

Supplementary Figure 3

SPRIZ (CCDC127) localizes to mitochondria as an integral OMM protein whose coiled-coil and C-terminal regions are exposed to the IMS

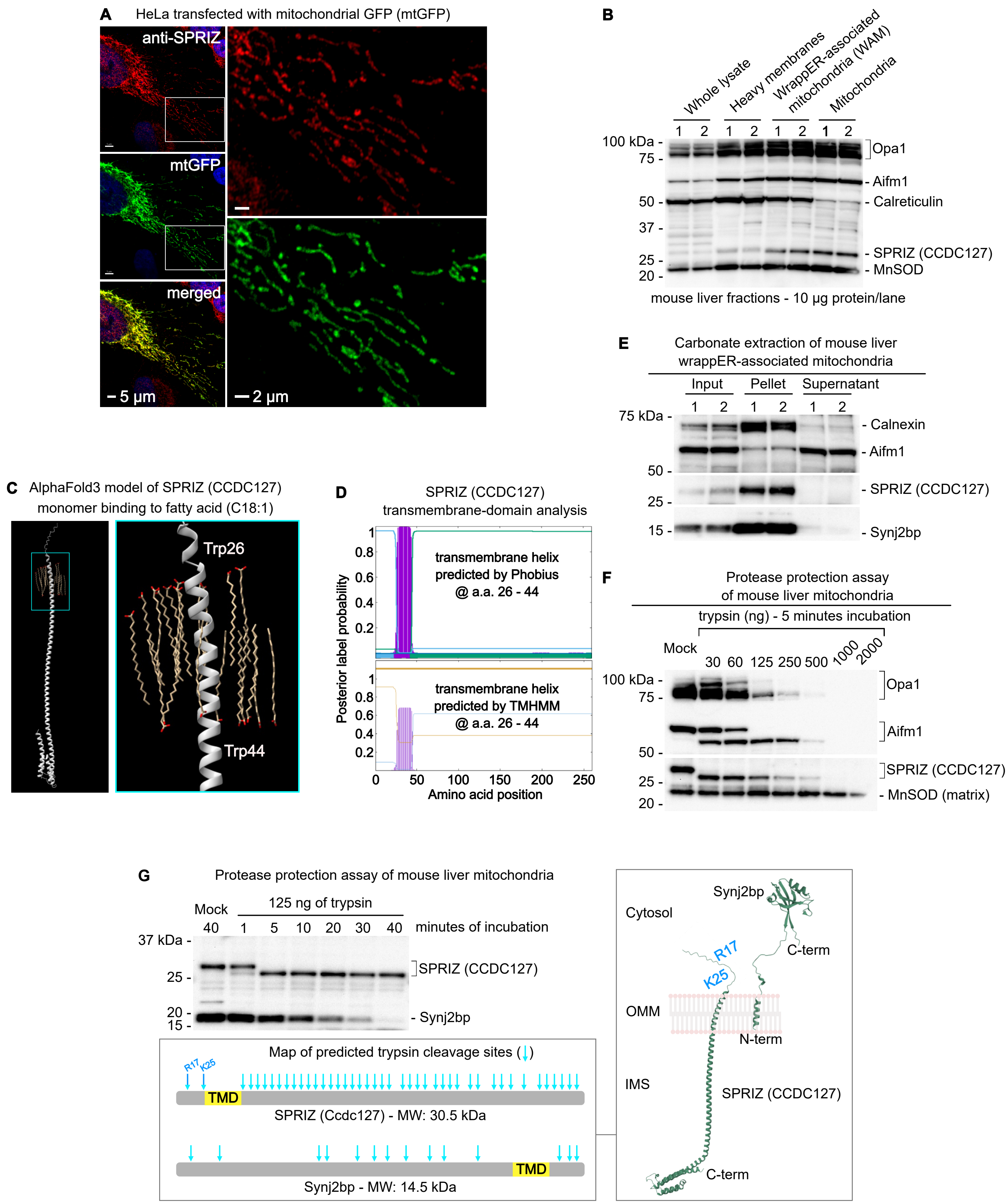

Fig. S3. SPRIZ (CCDC127) localizes to mitochondria as an integral OMM protein whose coiled-coil and C-terminal regions are exposed to the IMS.

(A) Immunofluorescence localization of endogenous SPRIZ (CCDC127) in HeLa cells transfected with mitochondrial matrix-targeted GFP (mtGFP; a gift of Heidi McBride - McGill University). Cells were stained with a commercially available anti-CCDC127 (SPRIZ) antibody (red) and imaged for mtGFP (green); merged image shows extensive colocalization in a filamentous mitochondrial network. Insets show zoomed regions (scale bars: 5 µm main, 2 µm insets). **(B)** Immunoblot analysis of SPRIZ (CCDC127) enrichment in subcellular fractions from mouse liver. Equal protein loading (10 µg/lane) from whole lysate, heavy membranes, wrappER-associated mitochondria (WAM), and purified mitochondria. Markers: OPA1 and AIFM1 (mitochondrial), calreticulin (wrappER), MnSOD/SOD2 (mitochondrial matrix). SPRIZ is highly enriched in mitochondrial fractions. **(C)** AlphaFold3 structural model of SPRIZ (CCDC127) monomer showing predicted interaction with oleic acid (C18:1 fatty acid) at the predicted transmembrane helix. **(D)** Transmembrane helix prediction for SPRIZ (CCDC127) using Phobius (top) and TMHMM (bottom). Both tools predict a single transmembrane helix (purple bar) spanning amino acids ~26–44 with high posterior probability, consistent with OMM anchoring. **(E)** Sodium carbonate extraction (pH 11.4) of wrappER-associated mitochondria-enriched fractions from mouse liver, separating integral membrane proteins (pellet) from peripheral and soluble proteins (supernatant). Input, pellet, and supernatant fractions were immunoblotted for calnexin (wrappER integral), AIFM1 (IMS), SPRIZ (CCDC127), and Synj2bp (OMM-associated). SPRIZ partitions to the pellet, confirming integral membrane association. **(F)** Protease protection assay of mouse liver mitochondria. Isolated mouse liver mitochondria were incubated with increasing amounts of trypsin (30–2,000 ng) for 5 min or left untreated (Mock), followed by immunoblotting for Opa1, Aifm1, SPRIZ (CCDC127), and the matrix protein MnSOD. **(G)** Time-course protease protection assay on isolated mouse liver mitochondria with 125 ng trypsin (0–40 min incubation). SPRIZ (CCDC127) shows immediate appearance of a slightly lower MW band due to cleavage at two predicted trypsin sites (R^{17} and K^{25}) in the short cytosol-exposed N-terminal tail, followed by progressive degradation of the bulk IMS-exposed domain. The N-terminal fragment remains largely protected, while Synj2bp (OMM-anchored control with larger cytosolic domain) is digested more rapidly and completely. Bottom panel: Schematic of predicted trypsin cleavage sites (↓) relative to transmembrane domains (TMD, yellow). Right panel: AlphaFold3 model of SPRIZ and Synj2bp anchored in the OMM for topology and interpretative comparison.

Supplementary Figure 4

Specificity validation of the commercial anti-CCDC127 antibody on endogenous and recombinant SPRIZ

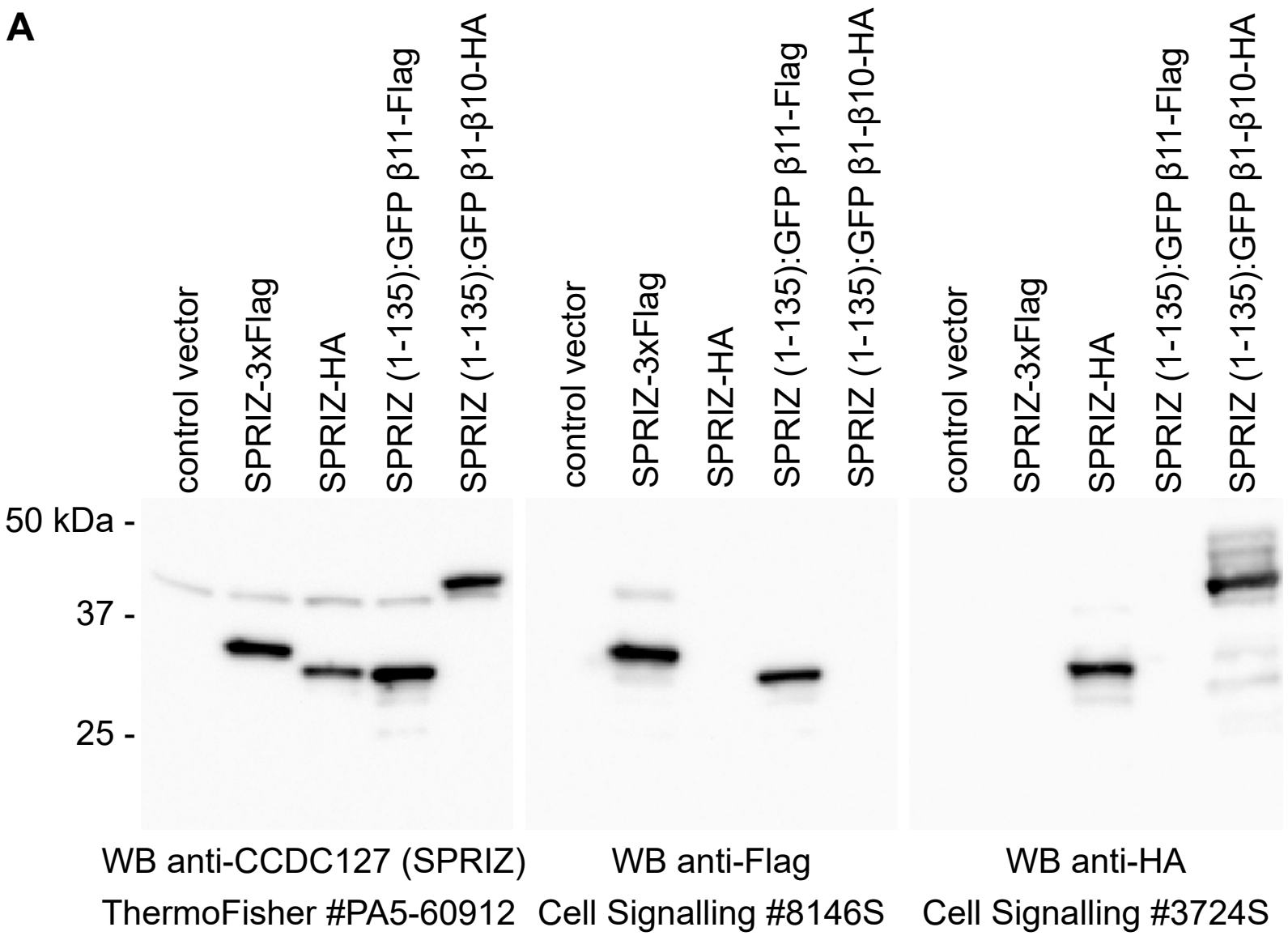

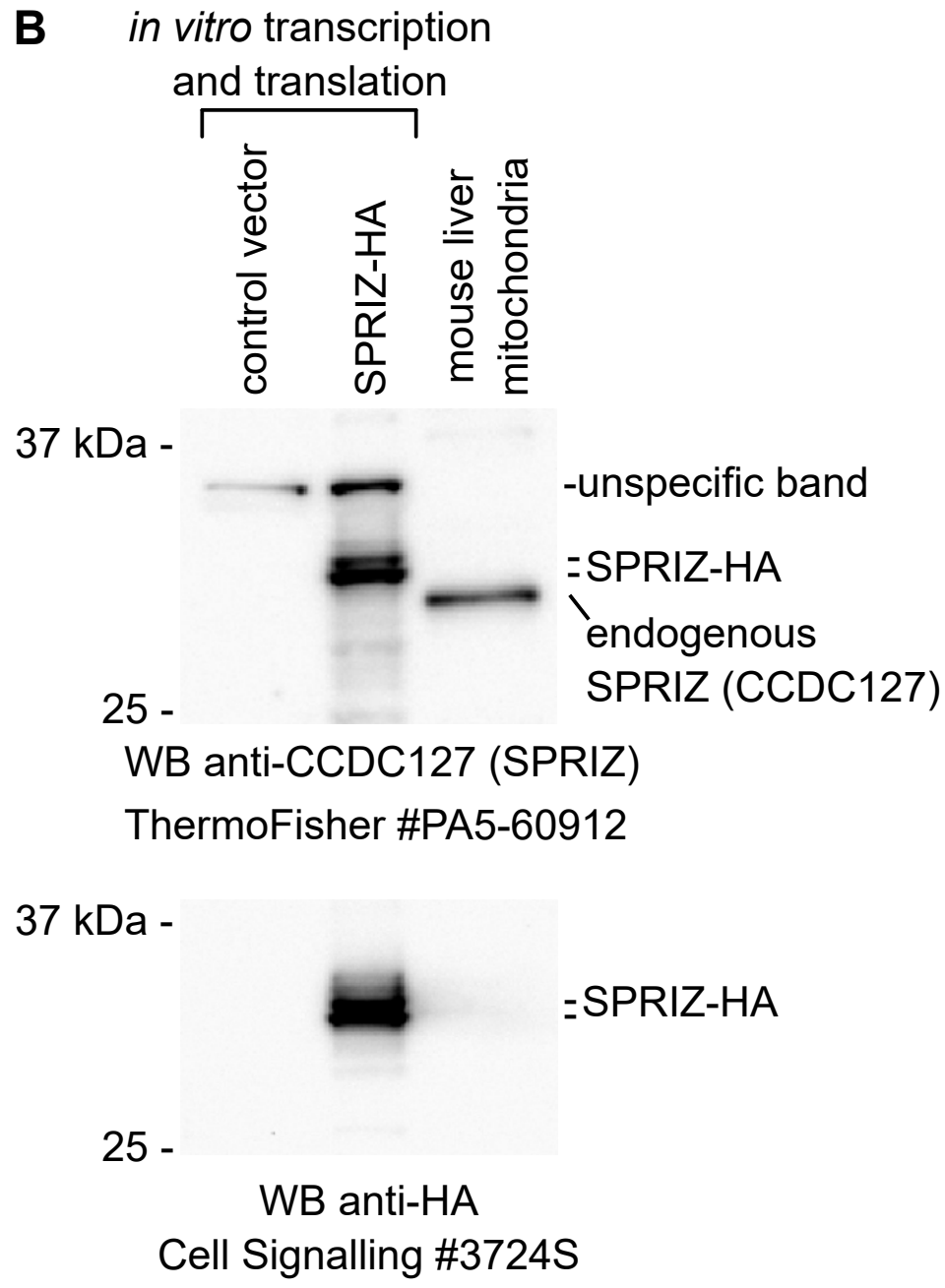

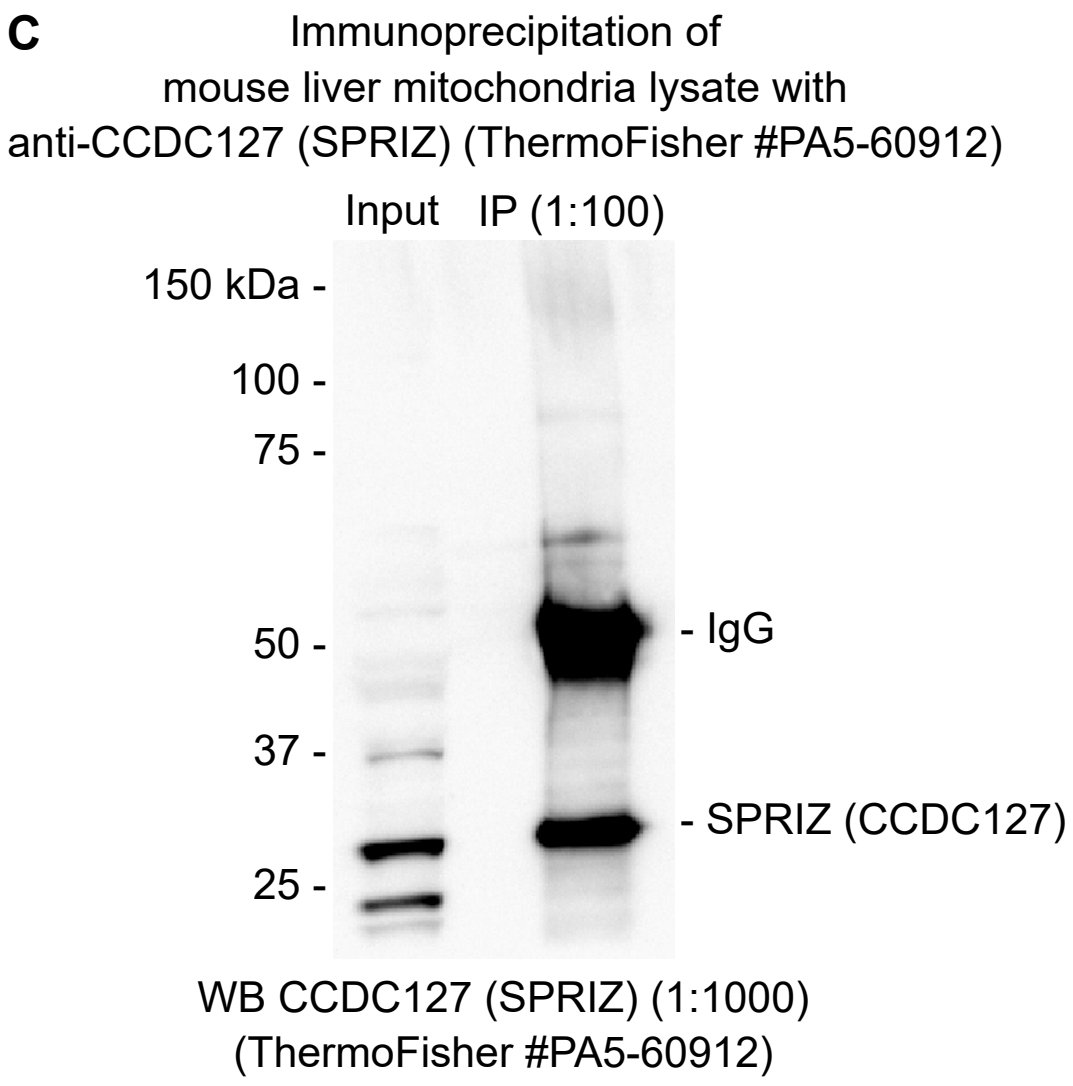

Fig. S4. Specificity validation of the commercial anti-CCDC127 antibody on endogenous and recombinant SPRIZ.

(A) Western blot validation of the commercially available anti-CCDC127 antibody (Thermo Fisher #PA5-60912) and tagged expression constructs in transfected cells. Lysates from HEK293 cells transfected with control vector, SPRIZ-3xFlag, SPRIZ-HA, $SPRIZ_{1-135}$-sfGFP(β_{11})x7-Flag, $SPRIZ_{1-135}$-sfGFP(β_{1-10})-HA, or combinations with Flag/HA tags were probed with anti-CCDC127 (left), anti-Flag (middle), and anti-HA (right). The antibody specifically detects full-length SPRIZ and N-terminal truncation constructs with no cross-reactivity to GFP or empty vector controls. **(B)** *In vitro* transcription and translation (IVT) of SPRIZ constructs followed by immunoblotting. Coupled IVT reactions (control vector, control-HA, SPRIZ-HA) and isolated mouse liver mitochondria were resolved and blotted with anti-CCDC127 (Thermo Fisher #PA5-60912; top) and anti-HA (bottom). The antibody detects endogenous SPRIZ in mitochondria (~30 kDa) and the IVT-produced SPRIZ-HA (~32 kDa, shifted by tag), with a consistent unspecific band in IVT reactions serving as loading/negative control. No SPRIZ signal is detected in the control vector reaction (left lane). **(C)** Immunoprecipitation specificity of the commercially available anti-CCDC127 antibody (Thermo Fisher #PA5-60912) from lysates of mouse liver mitochondria-enriched fractions. Input and immunoprecipitated material (using anti-CCDC127 at 1:100) were resolved and immunodetected with the same antibody (1:1000). The antibody efficiently immunoprecipitates endogenous SPRIZ (~30 kDa) along with IgG heavy chain (~50 kDa). **(D)** Anti-CCDC127 antibody specificity in mouse heart mitochondria. Western blot of mitochondrial lysates from *Ccdc127*$^{+/+}$ and *Ccdc127*$^{-/-}$ hearts probed with anti-CCDC127 antibody. The specific SPRIZ band is present only in wild-type samples and completely absent in knockout samples, whereas non-specific bands migrating around 37 kDa are detected in both genotypes.

Supplementary Figure 5

***Ccdc127* mRNA expression across 30 adult mouse tissues according to the ProteomicsDB resource and 54 cell lines according to Protein Atlas**

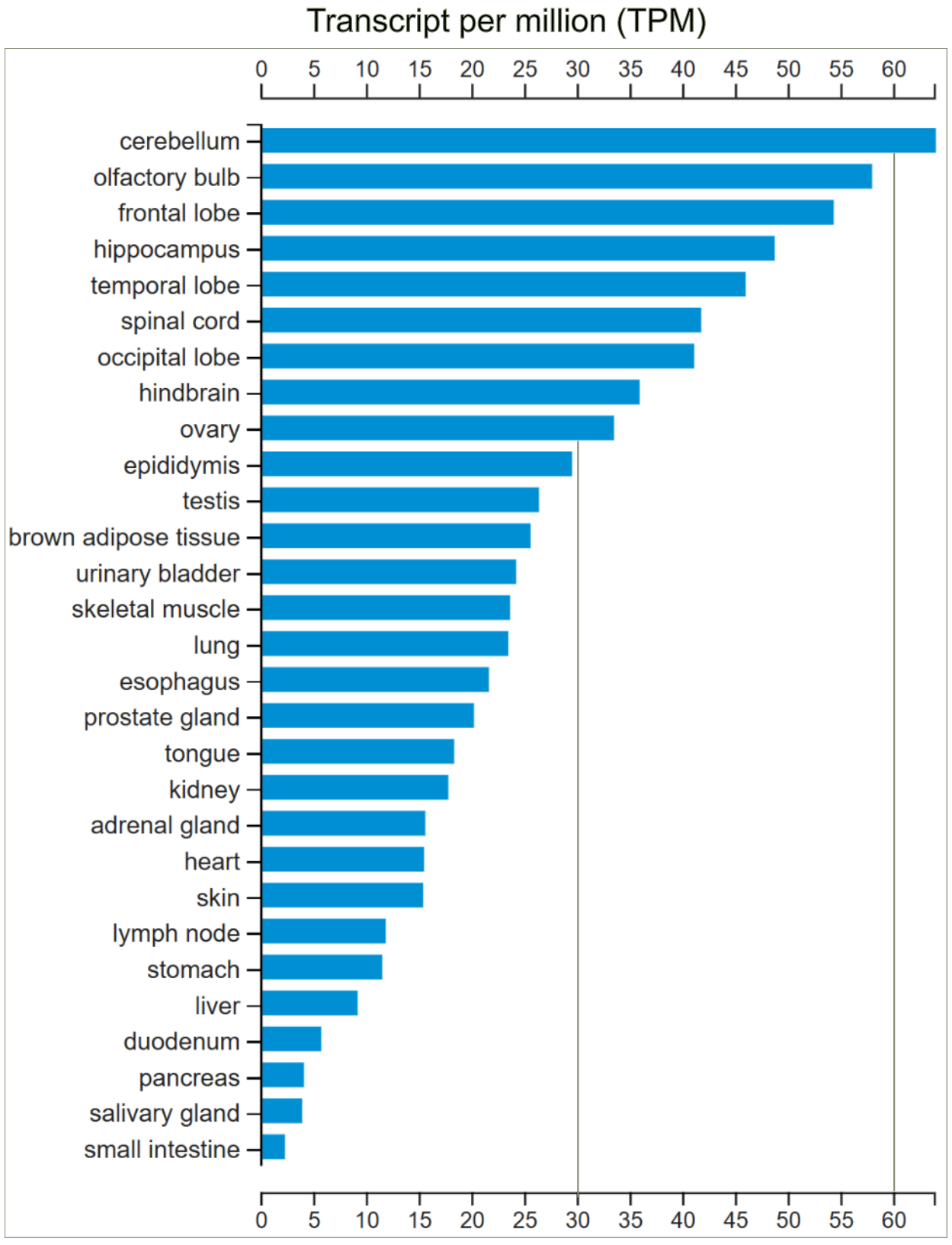

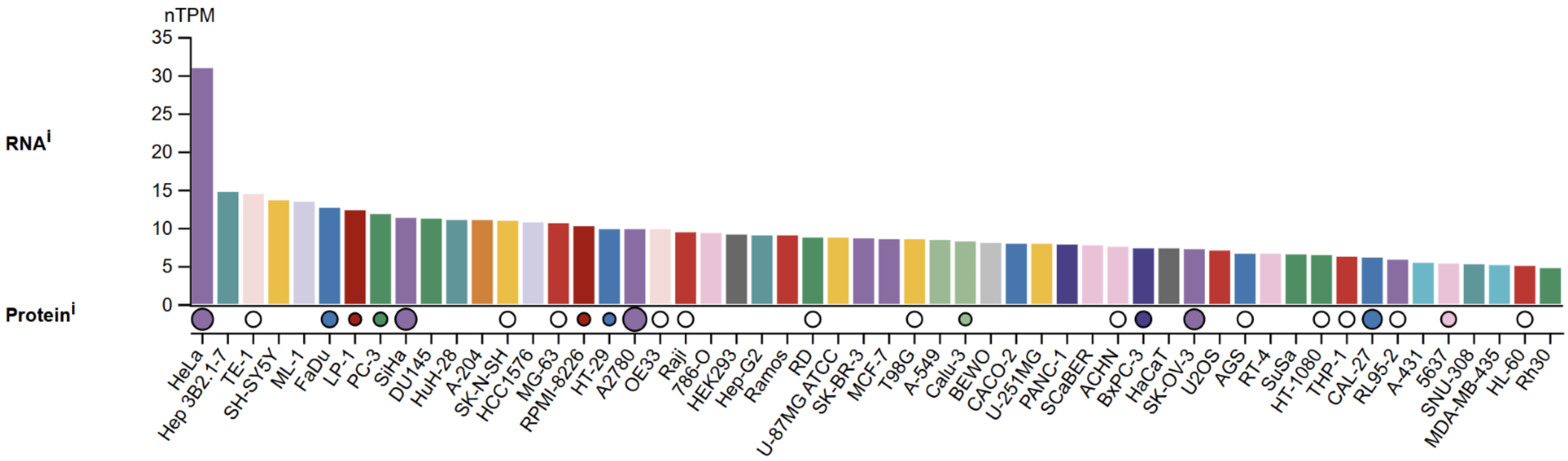

Fig. S5. ***Ccdc127*** **mRNA expression across 30 adult mouse tissues according to the ProteomicsDB resource and 54 cell lines according to Protein Atlas.**

Horizontal bar chart showing Ccdc127 mRNA levels in transcripts per million (TPM) across 30 adult mouse tissues, ordered from highest to lowest expression (data retrieved from the ProteomicsDB database (63-66)). Expression is highest in the cerebellum and other brain regions, moderate in reproductive tissues, brown adipose tissue, urinary bladder, and skeletal muscle, and lowest in gastrointestinal tissues and liver. Lower panel shows Ccdc127 transcript and protein evidence across 54 human cell lines according to the Human Protein Atlas.

Supplementary Figure 6

Extended quantitative proteomics and ultrastructural morphometry of liver mitochondria in *Ccdc127* knockout mice

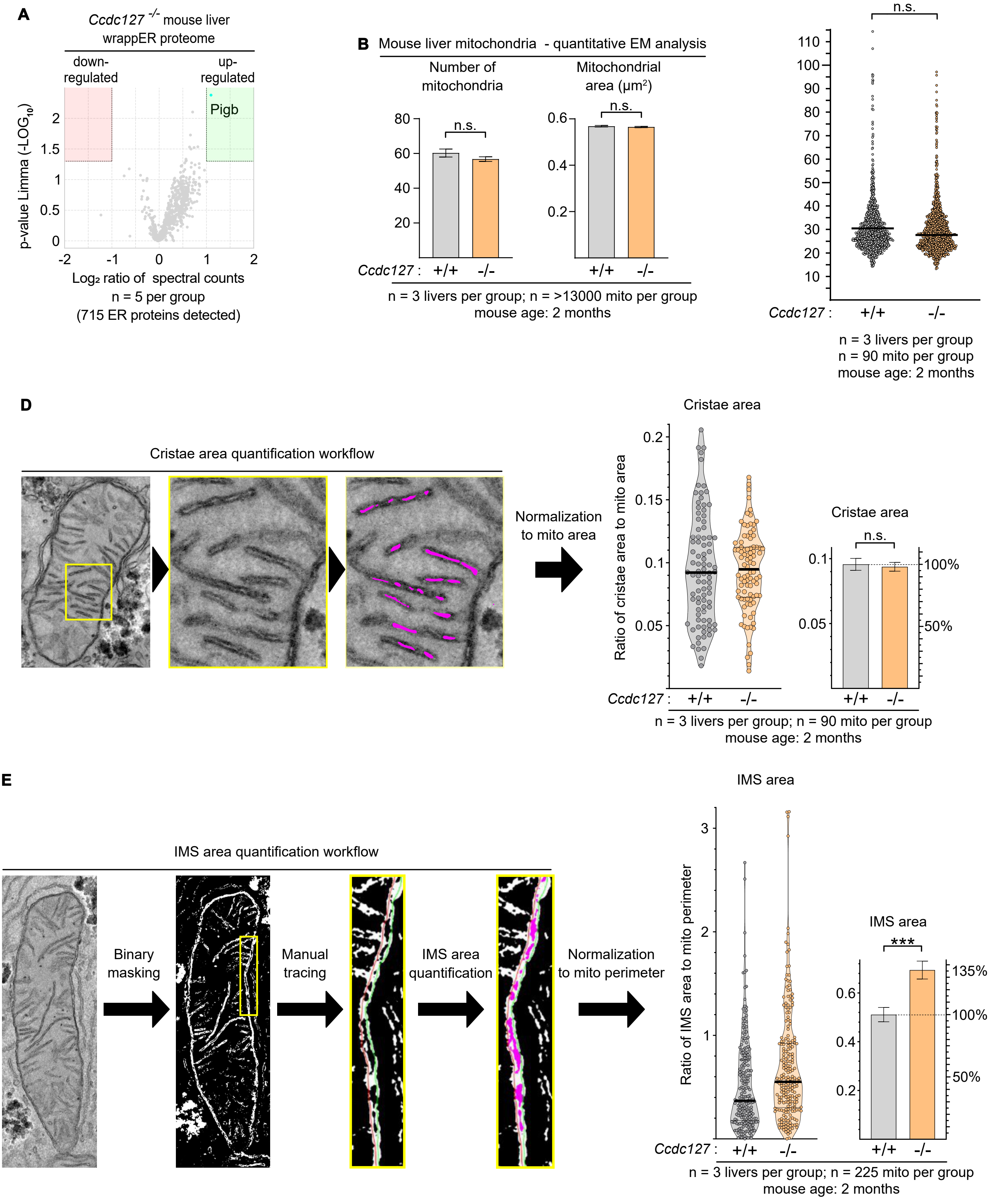

Supplementary Figure 6

Extended quantitative proteomics and ultrastructural morphometry of liver mitochondria in *Ccdc127* knockout mice

F Representative electron micrographs of wild-type (*Ccdc127* $^{+/+}$) mouse liver mitochondria

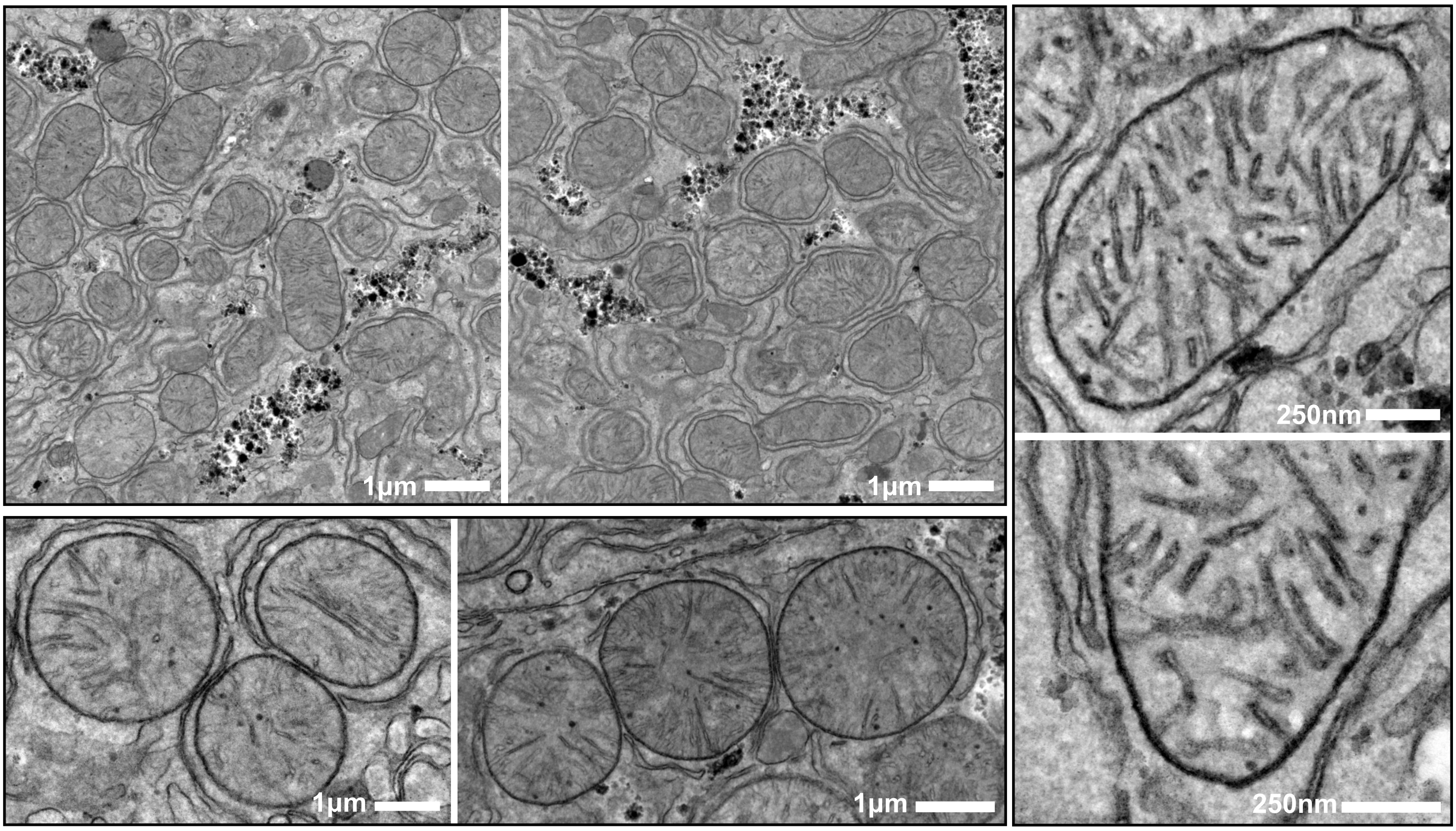

Fig. S6. Extended quantitative proteomics and ultrastructural morphometry of liver mitochondria in *Ccdc127* knockout mice.

(A) Volcano plot of quantitative label-free proteomics from mouse liver wrappER-associated mitochondria-enriched fractions (control vs. *Ccdc127* knockout; *n* = 5 per group) showing no major upregulation or downregulation of ER proteins (thresholds at p <0.05 and $|\log_2$ fold change$|$ >1 indicated by dashed lines and colored areas). **(B)** Quantitative EM morphometry comparing wild-type (+/+) and *Ccdc127* knockout (-/-) liver mitochondria: number of mitochondria per 225 μm^2 tissue area (left), mean mitochondrial area (right) (*n* = 3 livers per group, >13,000 mitochondria per group analyzed; n.s., not significant by unpaired t-test). **(C)** Quantification of mitochondrial *cristae* width in hepatocyte mitochondria from *Ccdc127* wild-type (+/+) and knockout (−/−) mice. Individual dots represent measurements of individual *cristae*; horizontal bars indicate the mean (n.s., not significant by unpaired t-test; *n* = 3 livers per group; *n* = 90 mitochondria per group). **(D)** Workflow for quantitative EM analysis of mitochondrial *cristae*. Representative TEM images were subjected to manual tracing of *cristae* area, followed by quantification of total *cristae* area and normalization to mitochondrial area, yielding the *cristae* area–to–mitochondrial area metric used in Fig. 1L and fig. S12A-B. **(E)** Workflow for quantitative EM analysis of the IMS. Representative TEM image is subjected to binary masking, tracing of the outer and inner mitochondrial membranes, masking/quantification of intermembrane space, and statistical analysis, yielding the IMS area-to-perimeter metric used in Fig. 1M, Fig. 6C, fig. S12E. **(F)** Representative transmission electron micrographs of liver mitochondria from 8-week-old wild-type (*Ccdc127*$^{+/+}$) mice at multiple magnifications, illustrating preserved mitochondrial ultrastructure and *cristae* organization.

Supplementary Figure 7

Design of tagged SPRIZ expression constructs and sequence/structural conservation of SPRIZ orthologs across mammals and amphibians

A pCDNA3.1 SPRIZ - sfGFP β1-β10 - HA

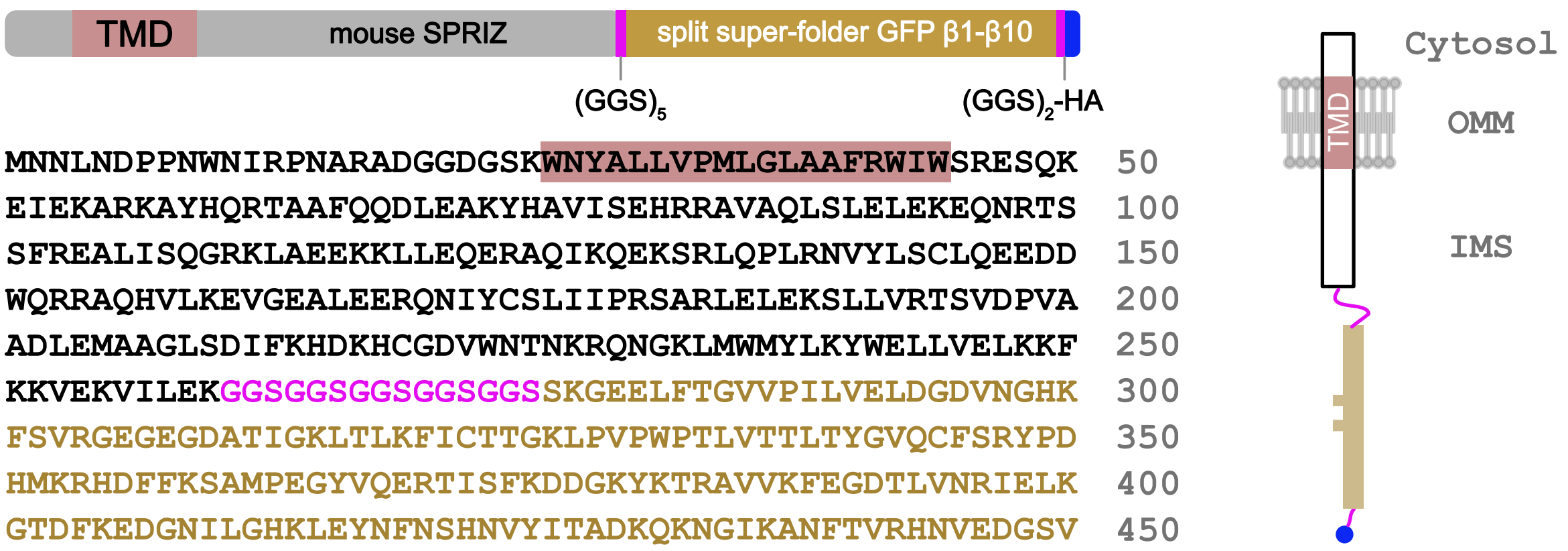


```
MNNLNDPPNWNIRPNARADGGDGSKWNYALLVPMLGLAAFRWIWSRESQK  50
EIEKARKAYHQRTAAFQQDLEAKYHAVISEHRRAVAQLSLELEKEQNRTS  100
SFREALISQGRKLAEEKKLLEQERAQIKQEKSRLQPLRNVYLSCLQEEDD  150
WQRRAQHVLKEVGEALEERQNIYCSLIIPRSARLELEKSLLVRTSVDPVA  200
ADLEMAAGLSDIFKHDKHCGDVWNTNKRQNGKLMWMYLKYWELLVELKKF  250
KKVEKVILEKGGSGGSGGSGGSGGSSKGEELFTGVVPILVELDGDVNGHK  300
FSVRGEGEGDATIGKLTLKFICTTGKLPVPWPTLVTTLTYGVQCFSRYPD  350
HMKRHDFFKSAMPEGYVQERTISFKDDGKYKTRAVVKFEGDTLVNRIELK  400
GTDFKEDGNILGHKLEYNFNSHNVYITADKQKNGIKANFTVRHNVEDGSV  450
QLADHYQQNTPIGDGPVLLPDNHYLSTQTVLSKDPNEKGGSGGSYPYDVP  500
DYA  503
```

B pcDNA3.1 SPRIZ - sfGFP β11 - Flag

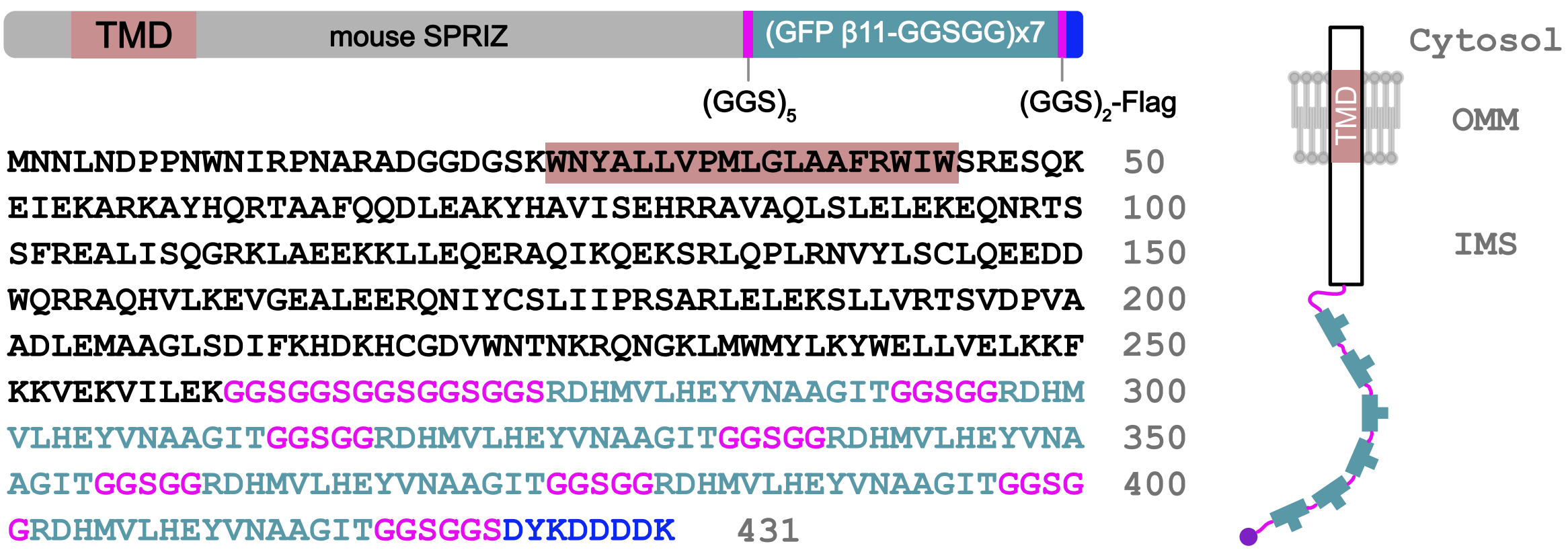


```
MNNLNDPPNWNIRPNARADGGDGSKWNYALLVPMLGLAAFRWIWSRESQK  50
EIEKARKAYHQRTAAFQQDLEAKYHAVISEHRRAVAQLSLELEKEQNRTS  100
SFREALISQGRKLAEEKKLLEQERAQIKQEKSRLQPLRNVYLSCLQEEDD  150
WQRRAQHVLKEVGEALEERQNIYCSLIIPRSARLELEKSLLVRTSVDPVA  200
ADLEMAAGLSDIFKHDKHCGDVWNTNKRQNGKLMWMYLKYWELLVELKKF  250
KKVEKVILEKGGSGGSGGSGGSGGSRDHMVLHEYVNAAGITGGSGGRDHM  300
VLHEYVNAAGITGGSGGRDHMVLHEYVNAAGITGGSGGRDHMVLHEYVNA  350
AGITGGSGGRDHMVLHEYVNAAGITGGSGGRDHMVLHEYVNAAGITGGSG  400
GRDHMVLHEYVNAAGITGGSGGSDYKDDDDK  431
```

C pCDNA3.1 SPRIZ-Flag

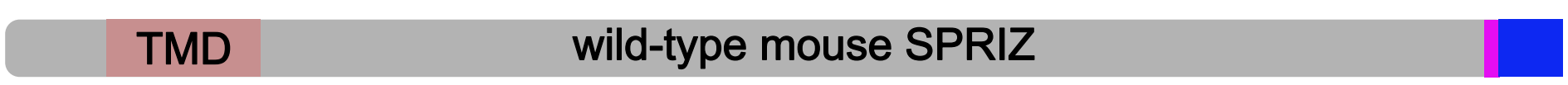

transmembrane domain (TMD)

```
MNNLNDPPNWNIRPNARADGGDGSKWNYALLVPMLGLAAFRWIWSRESQK  50
EIEKARKAYHQRTAAFQQDLEAKYHAVISEHRRAVAQLSLELEKEQNRTS  100
SFREALISQGRKLAEEKKLLEQERAQIKQEKSRLQPLRNVYLSCLQEEDD  150
WQRRAQHVLKEVGEALEERQNIYCSLIIPRSARLELEKSLLVRTSVDPVA  200
ADLEMAAGLSDIFKHDKHCGDVWNTNKRQNGKLMWMYLKYWELLVELKKF  250
KKVEKVILEKGGDYKDDDDK
```

Predicted SPRIZ-Flag MW: 31.6 kDa

Supplementary Figure 7

Design of tagged SPRIZ expression constructs and sequence/structural conservation of SPRIZ orthologs across mammals and amphibians

D

pCDNA3.1 SPRIZ-3xFlag

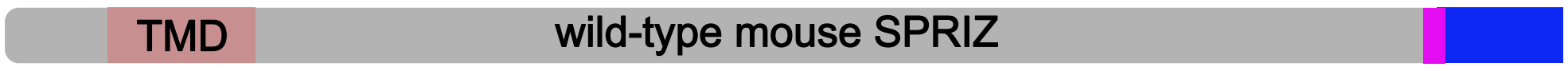

transmembrane domain (TMD)

```
MNNLNDPPNWNIRPNARADGGDGSKWNYALLVPMLGLAAFRWIWSRESQK  50
EIEKARKAYHQRTAAFQQDLEAKYHAVISEHRRAVAQLSLELEKEQNRTS 100
SFREALISQGRKLAEEKKLLEQERAQIKQEKSRLQPLRNVYLSCLQEEDD 150
WQRRAQHVLKEVGEALEERQNIYCSLIIPRSARLELEKSLLVRTSVDPVA 200
ADLEMAAGLSDIFKHDK*HCGDVWNTNKRQNGKLMWMYLKYWELLVELKKF 250
KKVEKVILEKGSENLYFQGSDYKDHDGDYKDHDIDYKDDDDK
```

Predicted SPRIZ-3xFlag MW: 34.2 kDa

E

pCDNA3.1 Xenopus-SPRIZ-Flag

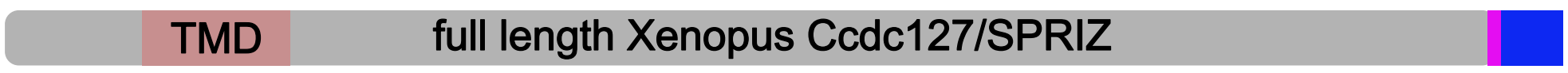

transmembrane domain (TMD)

```
MNNLNDPPQWNIHPNEDGREGSKWNYALLVPMLGLAAFRWIWSKESEKEI  50
SETKSECSKKLESAQKDLEAKYRDTITESRRTIAHLEIELEKEKNRTLSY 100
RRAIISQGQKLVEERKQLEQEREELFLEKQAVQKSGAAGTLYRSYILKEE 150
EWQKKATSLLKEFEEGLKERQNIYCSLVISRKERLEMEKNLIIKAVTDPI 200
AVELGMEAGLVDIFKHDTHCASFTNTNKRRNGRLMWLYLKYWELATERNK 250
FRKVEEAIIGEAKLETGGDYKDDDDK
```

Predicted Xenopus SPRIZ-Flag MW: 32.3 kDa

F

pCDNA3.1 SPRIZ-HA

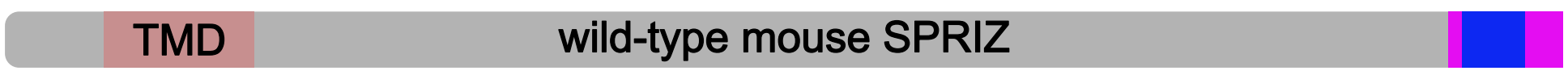

transmembrane domain (TMD)

```
MNNLNDPPNWNIRPNARADGGDGSKWNYALLVPMLGLAAFRWIWSRESQK  50
EIEKARKAYHQRTAAFQQDLEAKYHAVISEHRRAVAQLSLELEKEQNRTS 100
SFREALISQGRKLAEEKKLLEQERAQIKQEKSRLQPLRNVYLSCLQEEDD 150
WQRRAQHVLKEVGEALEERQNIYCSLIIPRSARLELEKSLLVRTSVDPVA 200
ADLEMAAGLSDIFKHDKHCGDVWNTNKRQNGKLMWMYLKYWELLVELKKF 250
KKVEKVILEKGGYPYDVPDYALESRGPV
```

Predicted SPRIZ-HA MW: 32.3 kDa

Supplementary Figure 7

Design of tagged SPRIZ expression constructs and sequence/structural conservation of SPRIZ orthologs across mammals and amphibians

G Pairwise protein sequence alignment of mammalian and amphibian SPRIZ / Ccdc127 orthologs

```
                                        transmembrane domain (TMD)
Xenopus  MNNLNDPPQWNIHPNE--DGREGSKWNYALLVPMLGLAAFRWIWSKESEKEISETKSECS
Mouse    MNNLNDPPNWNIRPNARADGGDGSKWNYALLVPMLGLAAFRWIWSRESQKEIEKARKAYH   60
         ********:***:**    ** :**********************:**:***.:::.

Xenopus  KKLESAQKDLEAKYRDTITESRRTIAHLEIELEKEKNRTLSYRRAIISQGQKLVEERKQL
Mouse    QRTAAFQQDLEAKYHAVISEHRRAVAQLSLELEKEQNRTSSFREALISQGRKLAEEKKLL   120
         ::   : *:******:  .*:* **::*:*.:*****:*** *:*.*:****:**.**:* *

Xenopus  EQEREELFLEKQAVQKSGAAGTLYRSYILKEEEWQKKATSLLKEFEEGLKERQNIYCSLV
Mouse    EQERAQIKQEKSRLQP---LRNVYLSCLQEEDDWQRRAQHVLKEVGEALEERQNIYCSLI   180
         **** ::  **. :*       .:* * : :*::**::*  :***. *.*:*********:

Xenopus  ISRKERLEMEKNLIIKAVTDPIAVELGMEAGLVDIFKHDTHCASFTNTNKRRNGRLMWLY
Mouse    IPRSARLELEKSLLVRTSVDPVAADLEMAAGLSDIFKHDKHCGDVWNTNKRQNGKLMWMY   240
         *.*. ***:**.*:::: .**:*.:* * *** ******.**...  *****:**:***:*

Xenopus  LKYWELATERNKFRKVEEAIIGEAKLET  NP_001016568.1  Identity:   57 %
Mouse    LKYWELLVELKKFKKVEKVILEK-----  Q3TC33          Similarity: 73 %
         ****** .* :**:***:.*: :
```

H AlphaFold3 models

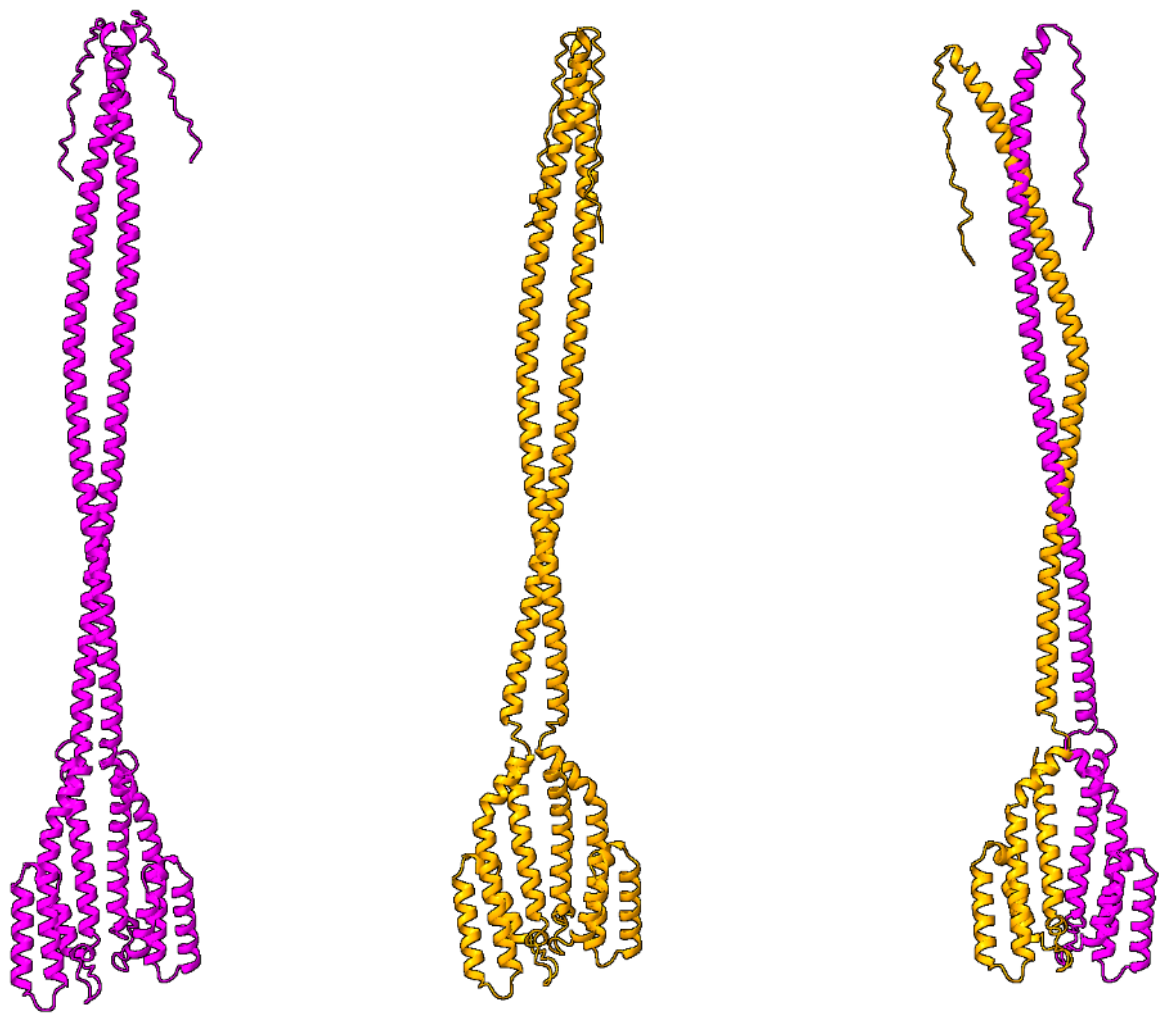

Xenopus SPRIZ homodimer — mouse SPRIZ homodimer — Xenopus / mouse SPRIZ hybrid homodimer

Supplementary Figure 7

Design of tagged SPRIZ expression constructs and sequence/structural conservation of SPRIZ orthologs across mammals and amphibians

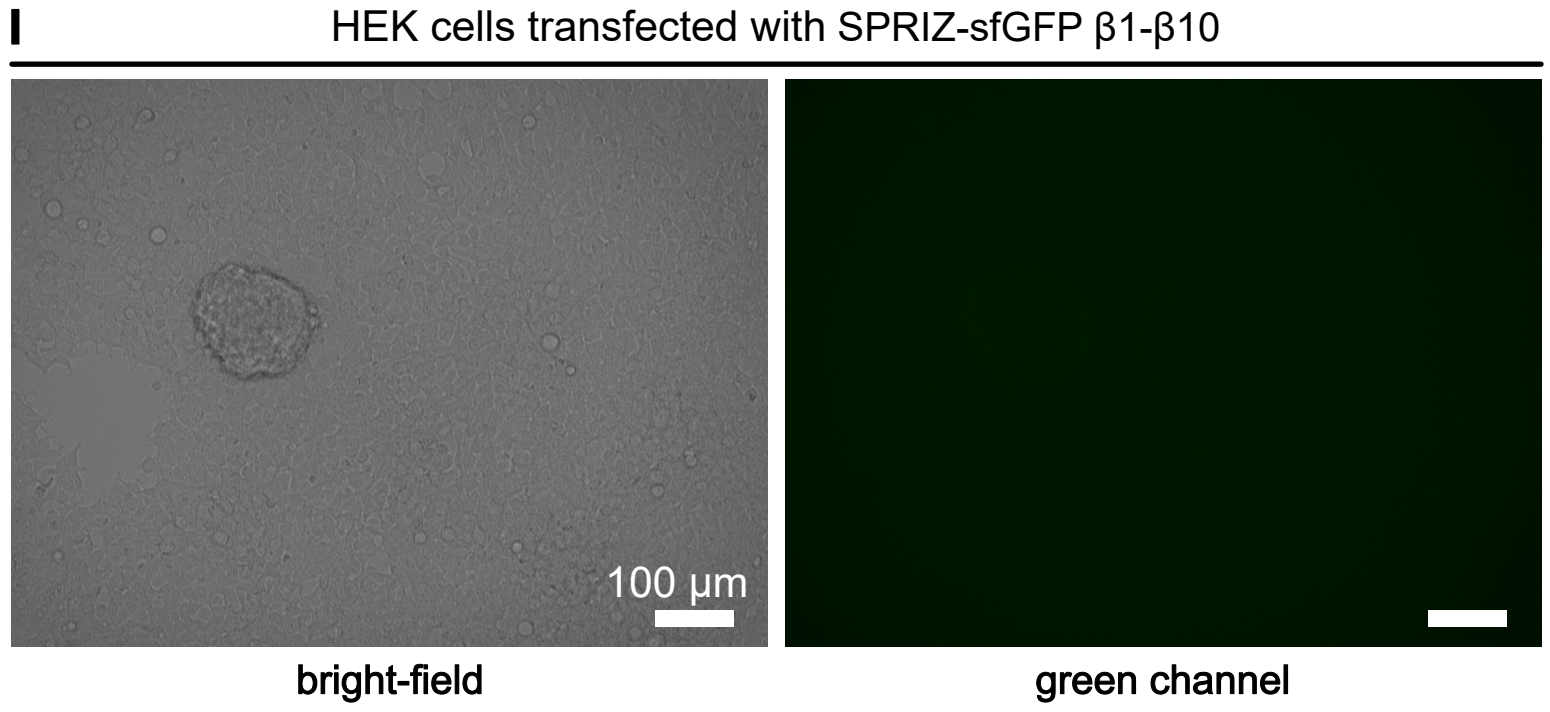

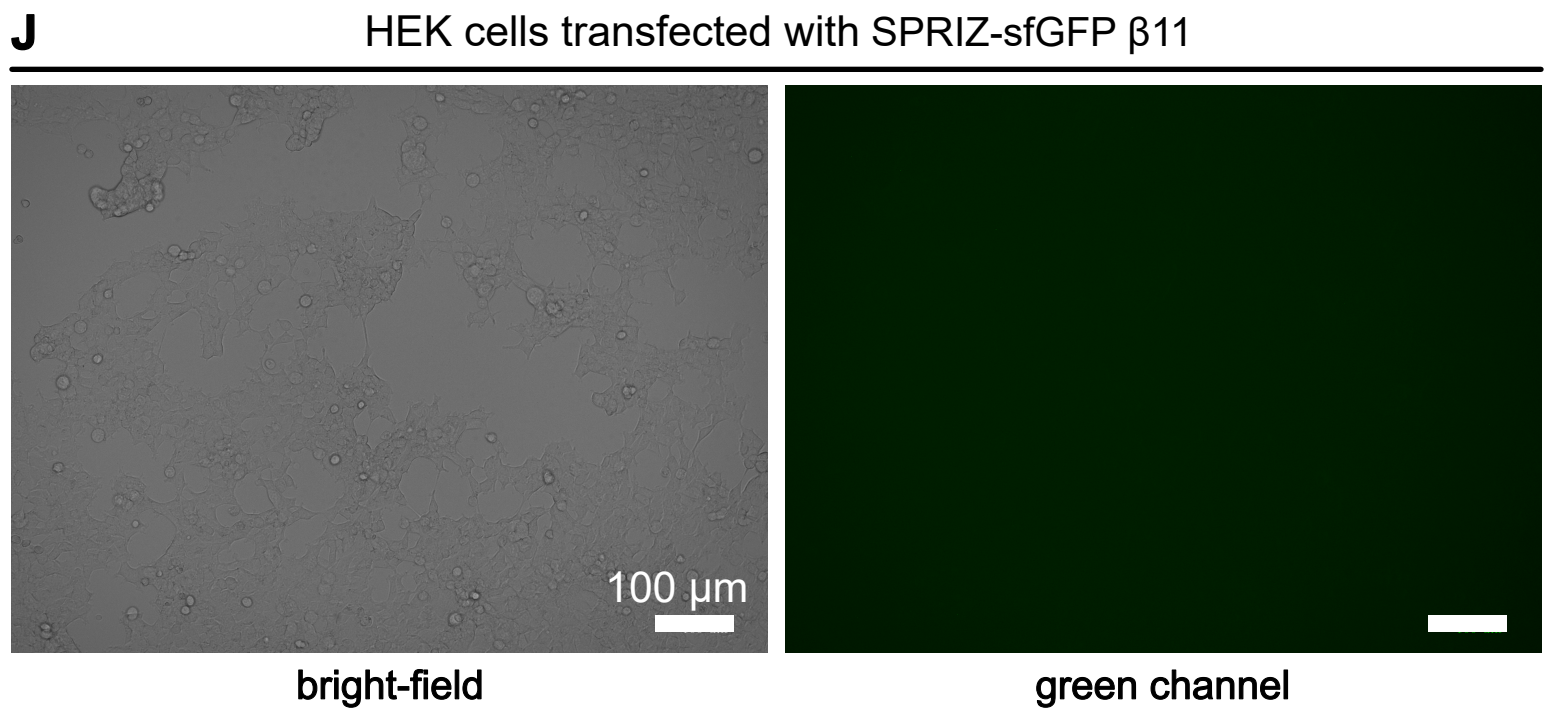

Fig. S7. Design of tagged SPRIZ expression constructs and sequence/structural conservation of SPRIZ orthologs across mammals and amphibians.

(A–F) Schematics of SPRIZ (CCDC127) expression constructs in the pcDNA3.1 vector. Each construct shows domain architecture (N-terminal transmembrane domain in brown), tag position and sequence, flexible linker ($(GGS)_n$ or equivalent), predicted molecular weight (MW, in kDa), and amino-acid numbering. All constructs anchor SPRIZ to the outer mitochondrial membrane (OMM) via the N-terminal TMD, with the coiled-coil and C-terminal domains facing the intermembrane space (IMS; right-hand topology diagram). **(G)** Pairwise sequence alignment of mouse and *Xenopus* SPRIZ orthologs. Clustal-style alignment highlights 57% sequence identity and 73% similarity (mouse Q3TC33 vs. *Xenopus* NP_001016568.1). The transmembrane domain (TMD) is highlighted in brown; conserved residues are marked (* identical, : conserved substitution, . semi-conserved). The alignment demonstrates strong conservation across the TMD and coiled-coil regions, supporting functional orthology in vertebrates. **(H)** AlphaFold3 models of SPRIZ homodimers. Ribbon representations show the *Xenopus* SPRIZ homodimer (magenta), mouse SPRIZ homodimer (yellow/orange), and a hybrid *Xenopus*/mouse SPRIZ homodimer. The models illustrate high structural similarity, compatible dimer interfaces, and conserved elongated coiled-coil architecture despite sequence divergence shown in panel G. **(I–J)** Negative controls for the split-superfolder GFP complementation assay. HEK293 cells were transfected with SPRIZ-sfGFP(β1–10) alone (I) or SPRIZ-sfGFP(β11) alone (J). Bright-field and green-channel images show absence of detectable GFP fluorescence when either split-GFP fragment is expressed individually. Scale bars, 100 µm.

Supplementary Figure 8

Molecular dynamics analysis of flexibility in the C-terminal conical domain of the SPRIZ homodimer

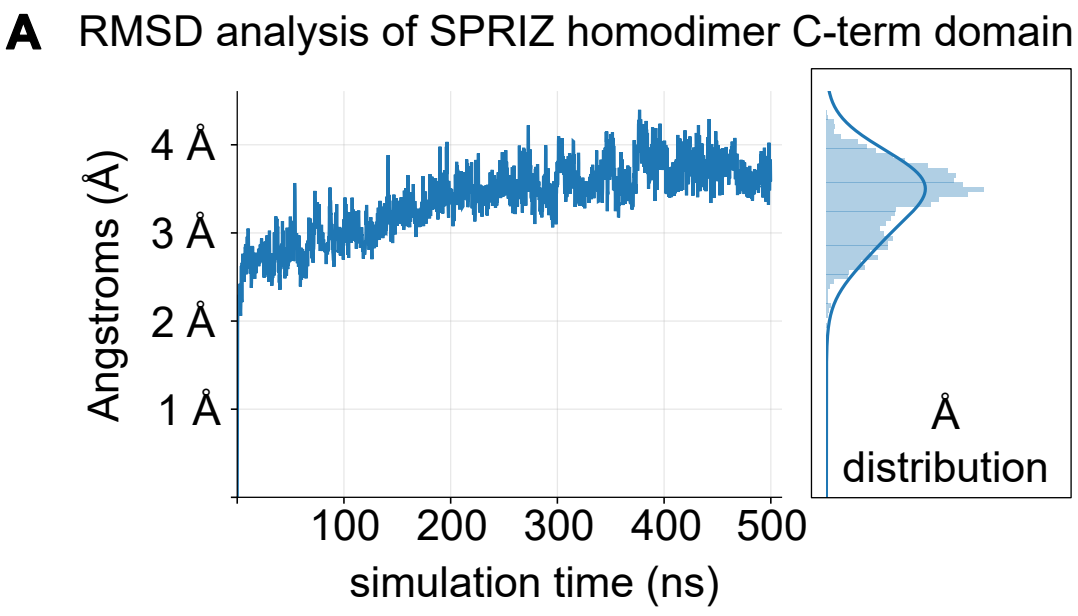

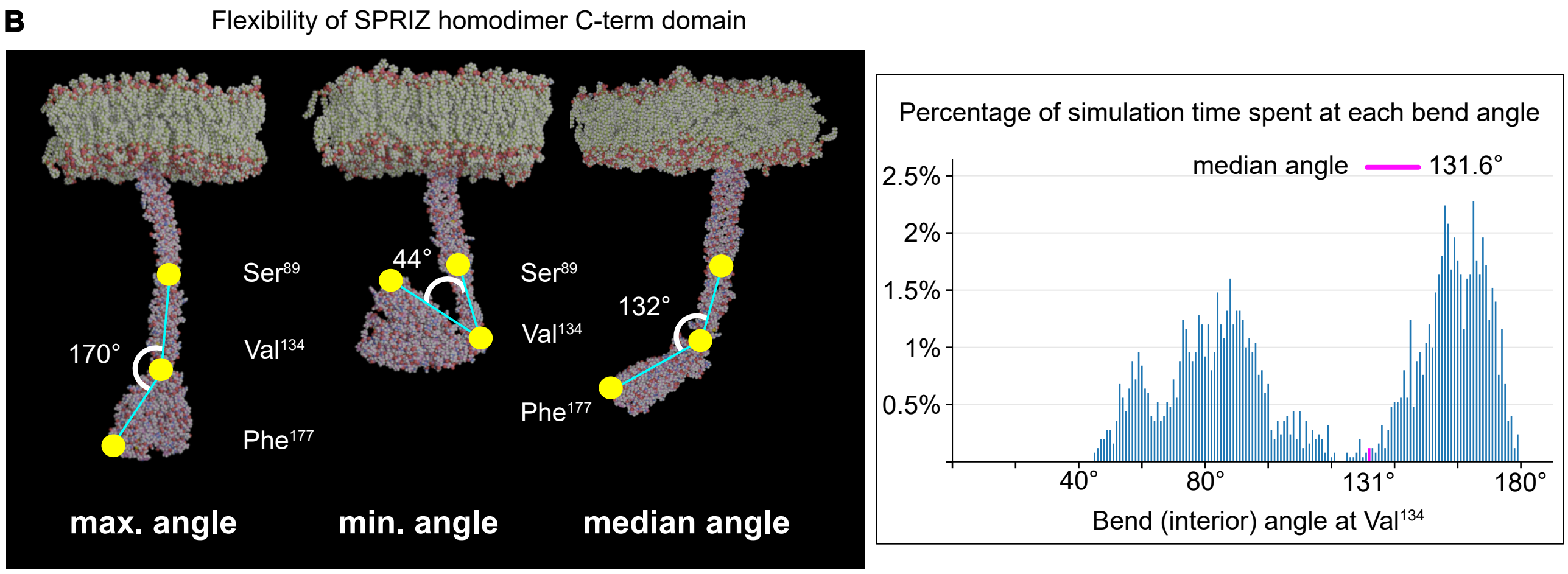

Fig. S8. Molecular dynamics analysis of flexibility in the C-terminal conical domain of the SPRIZ homodimer.

(A) Backbone RMSD of the SPRIZ homodimer C-terminal conical domain over 500 ns of all-atom MD simulation (see fig. 2H for the full homodimer trajectory). After initial equilibration, RMSD stabilizes at 3–3.5 Å with minor fluctuations up to ~4 Å. Inset shows the RMSD distribution across the trajectory (centered at ~3.2–3.3 Å), indicating high conformational stability of the C-terminal conical domain. **(B)** Bend-angle analysis of the SPRIZ homodimer C-terminal conical region. Left: Representative simulation snapshots showing the maximum (170°), minimum (44°), and median (131.6°) interior bend angles measured at Val^{134} (reference points: Ser^{89}, Val^{134}, Phe^{177}). The coiled-coil is shown in surface representation with key residues labeled. Right: Histogram of interior bend angles at Val^{134} across the trajectory (median 131.6°, magenta line). The broad, bimodal distribution demonstrates greater flexibility and dynamic bending in the C-terminal conical domain compared with the rigid coiled-coil core.

Supplementary Figure 9

STEP 1: Localization of a SPRIZ-like OMM–IMM bridging structure in a cryo-electron tomography

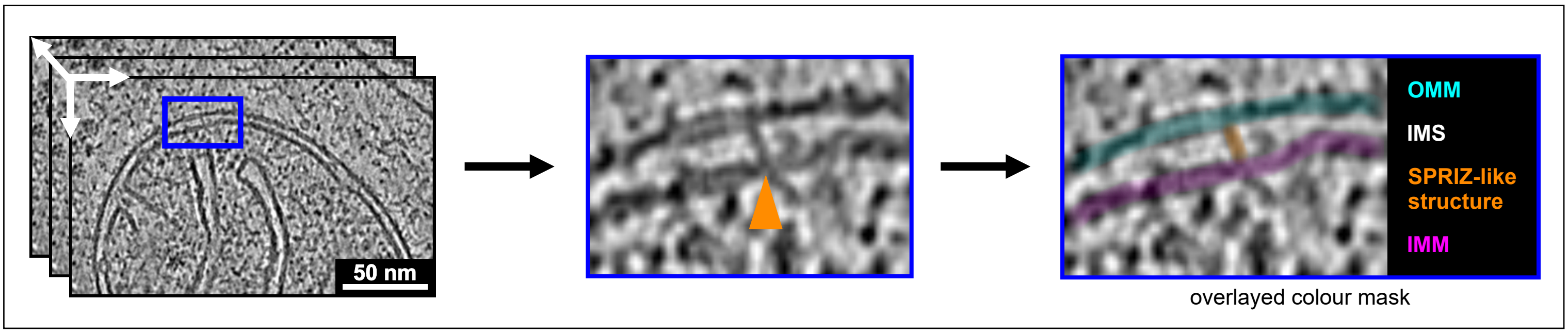

STEP 2: Tracking a SPRIZ-like structure across serial virtual sections of the cryo-electron tomography

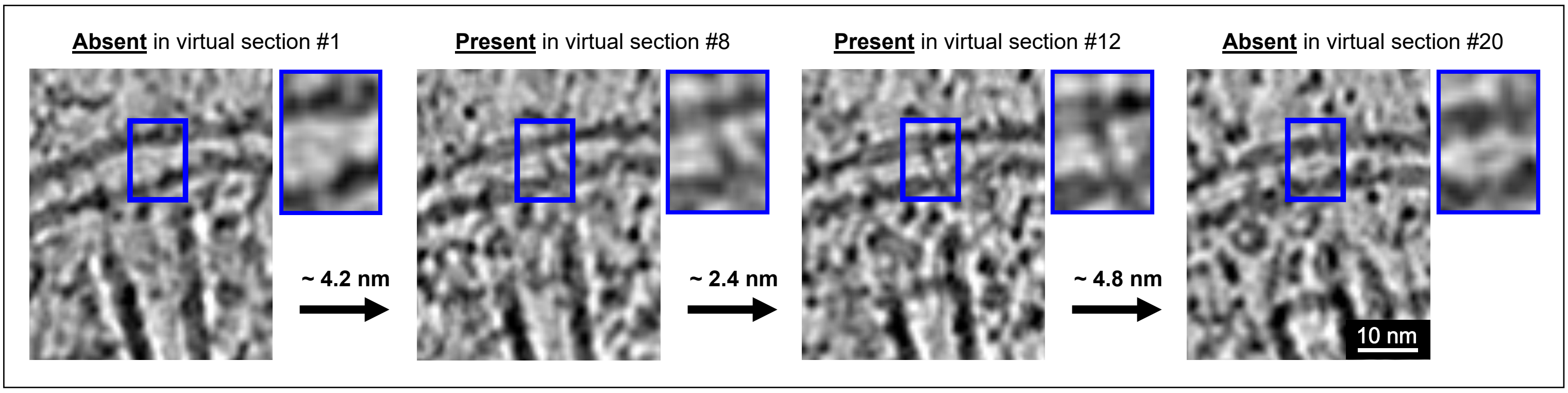

STEP 3: Generation of a 3D isosurface model from the cryo-electron tomography

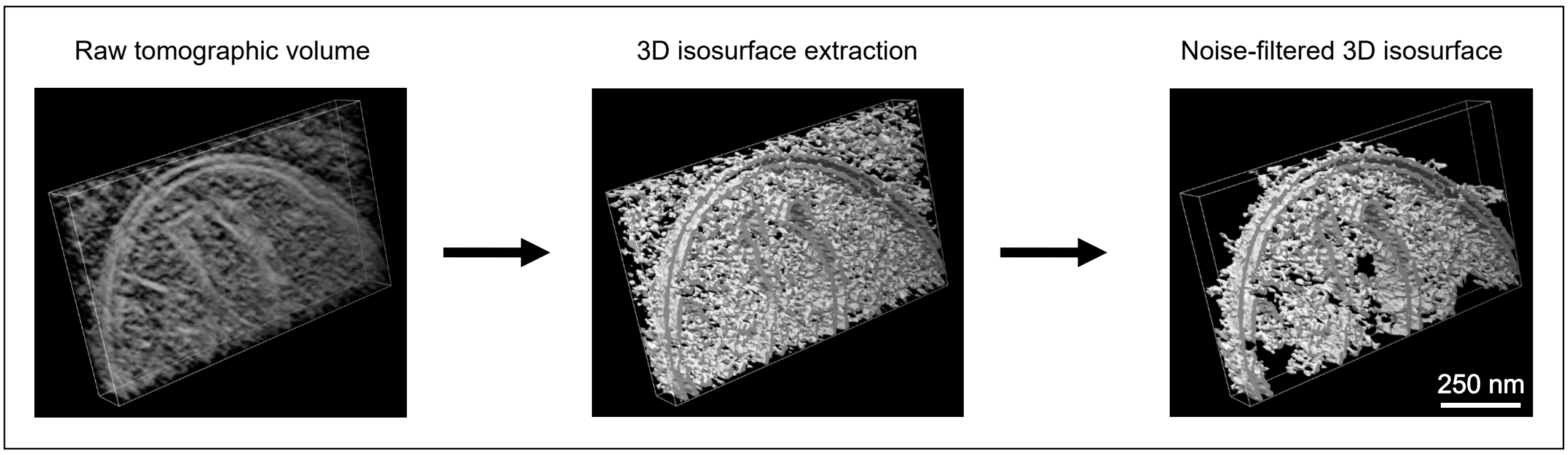

STEP 4: 3D reconstruction of a SPRIZ-like structure from cryo-electron tomography

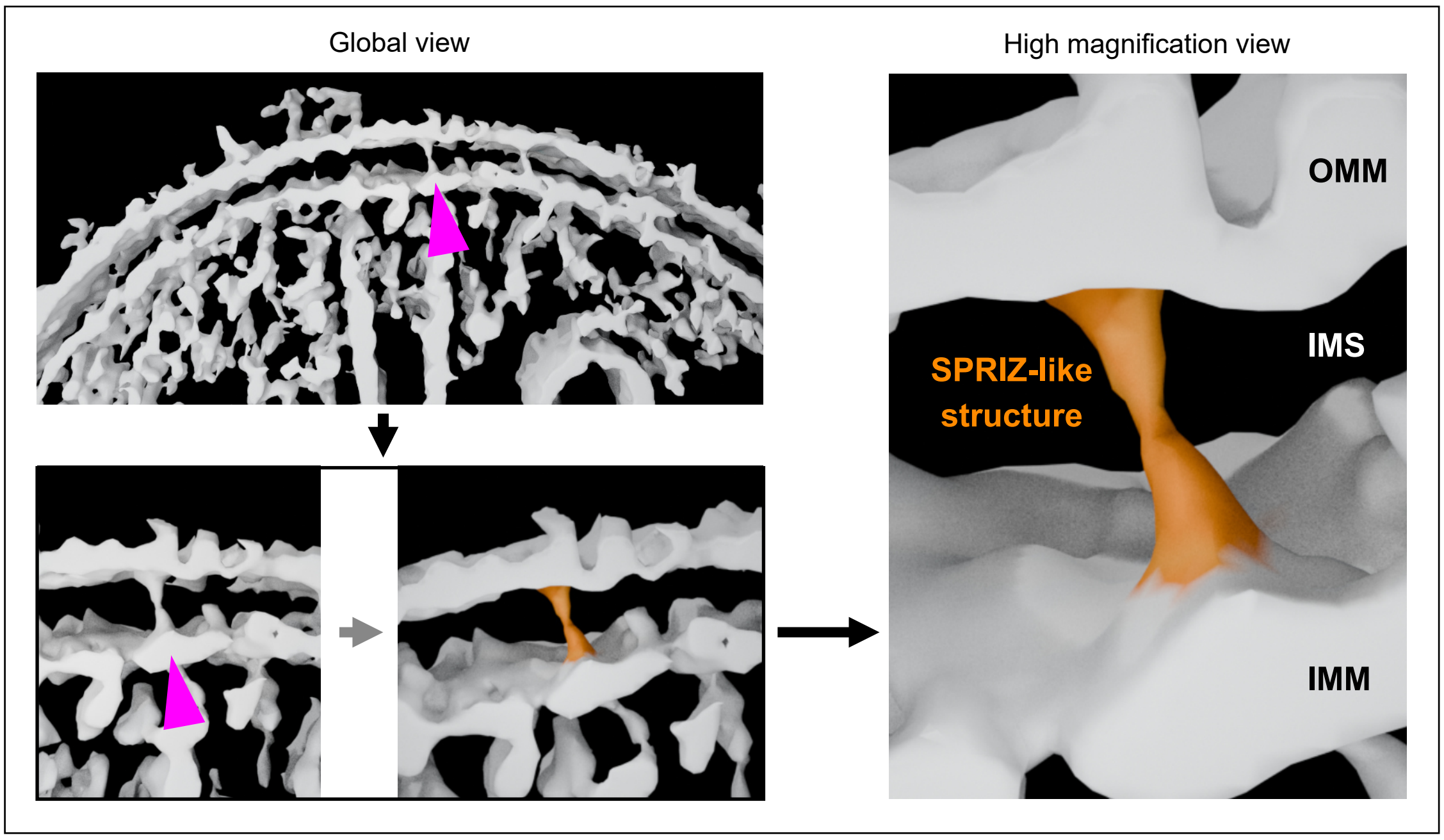

Fig. S9. Identification and 3D reconstruction of a SPRIZ-like OMM–IMM bridging structure in cryo-electron tomography data (41).

Step 1: Localization of a putative SPRIZ-like structure bridging the OMM and IMM in a cryo-electron tomogram. A region of interest containing a tether structure is enlarged and shown with an overlaid colour mask, highlighting the OMM, IMM, IMS, and the SPRIZ-like bridging density. **Step 2:** Tracking of the SPRIZ-like density across serial virtual sections of the same tomographic volume. The structure is absent from virtual section #1, becomes visible in sections #8 and #12, and is no longer detected in section #20, consistent with a discrete density spanning a limited z-depth within the tomogram. Approximate distances between the displayed virtual sections are indicated. **Step 3:** Generation of a 3D isosurface model from the cryo-electron tomographic volume. The raw tomographic volume was subjected to 3D isosurface extraction and subsequent noise filtering to visualize membrane-associated densities within the reconstructed mitochondrial region. **Step 4:** 3D reconstruction of the SPRIZ-like structure from the cryo-electron tomography dataset. Global and magnified views show the candidate density connecting the OMM and IMM across the IMS. The SPRIZ-like structure is highlighted in orange, while mitochondrial membranes are shown in grey/white. Data were derived from cryo-electron tomography datasets reported in TMX4-driven LINC complex disassembly and asymmetric autophagy of the nuclear envelope upon acute ER stress (41).

Supplementary Figure 10

Molecular dynamics analysis of the MARTINI homodimer showing high rigidity in the coiled-coil domain and constrained flexibility in the C-terminal conical domain

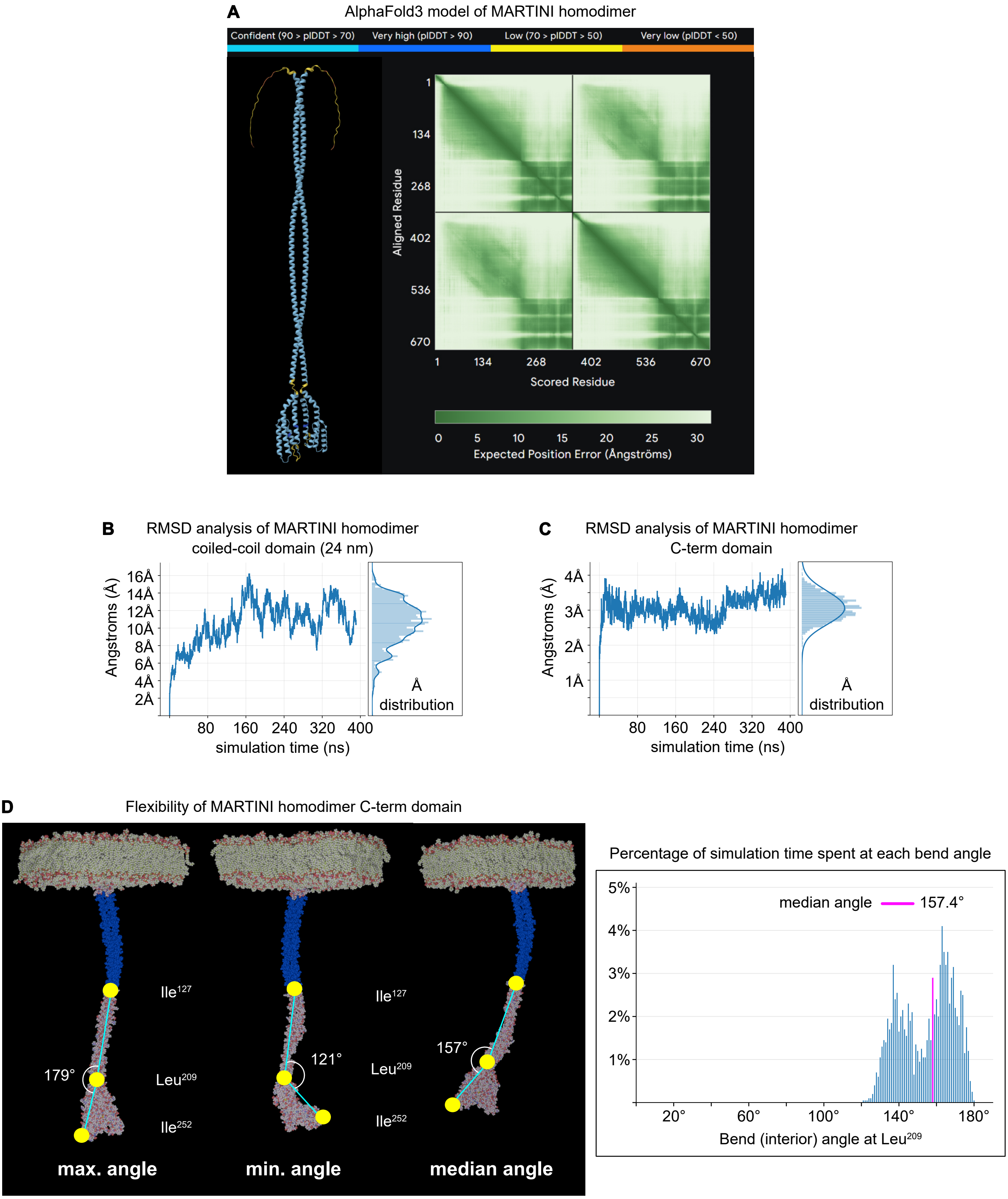

Fig. S10. Molecular dynamics analysis of the MARTINI homodimer showing high rigidity in the coiled-coil domain and constrained flexibility in the C-terminal conical domain.

(A) AlphaFold3 model of the MARTINI homodimer colored by per-residue confidence (pLDDT) and corresponding Predicted Aligned Error (PAE) matrix. High-confidence regions (coiled-coil rod and C-terminal conical domain) contrast with lower-confidence segments in the flexible linker. Pattern closely resembles SPRIZ (fig. 2B). **(B)** All-atom MD RMSD time course for the MARTINI coiled-coil domain (24 nm) over 400 ns, with RMSD distribution (right). The domain remains stable. **(C)** All-atom MD RMSD time course for the MARTINI C-terminal conical domain over the same trajectory, with RMSD distribution (right). **(D)** Bend-angle analysis of the C-terminal conical domain. Representative snapshots and histogram of interior bend angles at Leu^{209} (reference points Ile^{127}, Leu^{209}, Ile^{252}) show a median of ~157.4°, demonstrating the hinge-like, constrained flexibility of the distal conical module compared with the rigid coiled-coil core (fig. 4H).

Supplementary Figure 11

Ca^{2+} imaging, MCU complex abundance, and MAM contact analysis in SPRIZ- and MARTINI-expressing HeLa cells

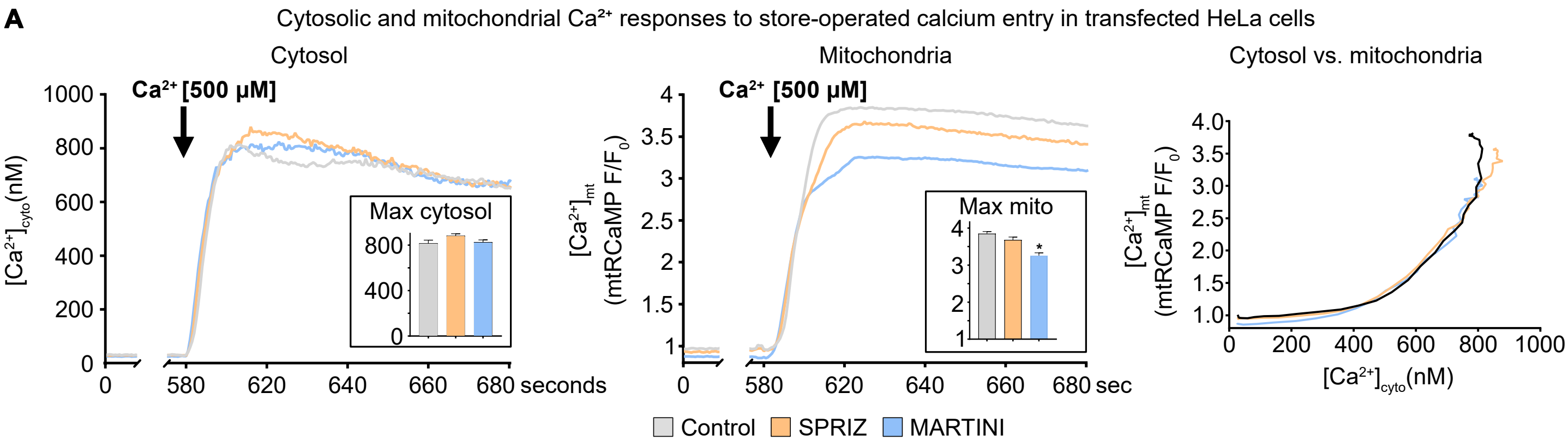

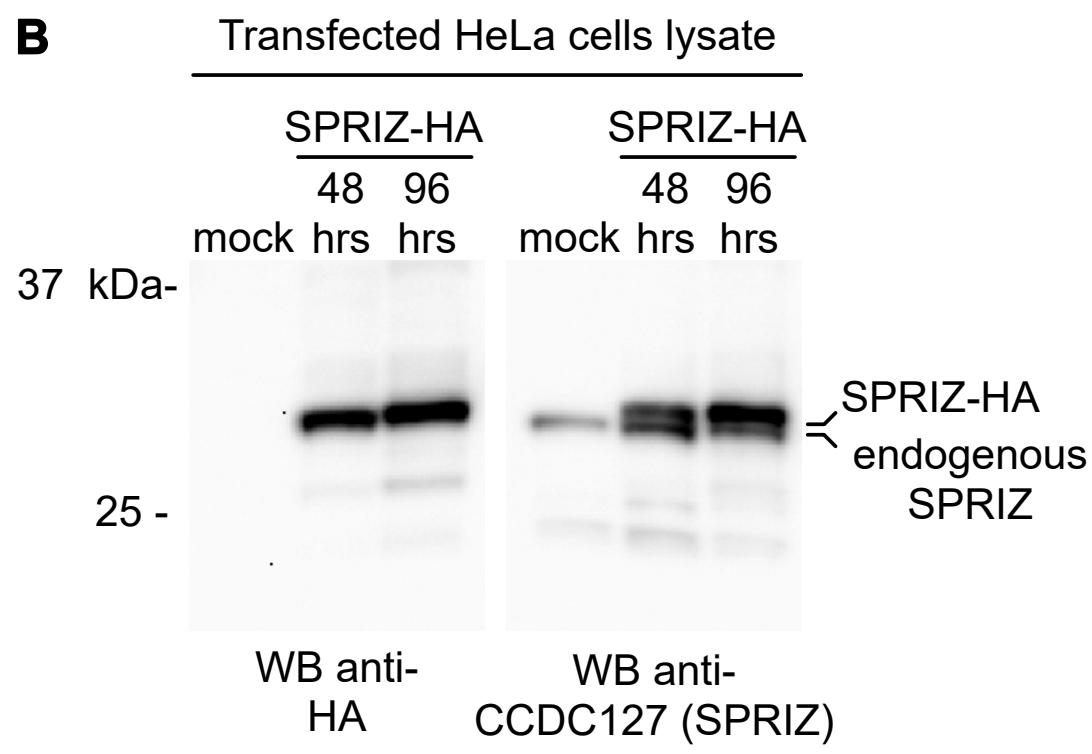

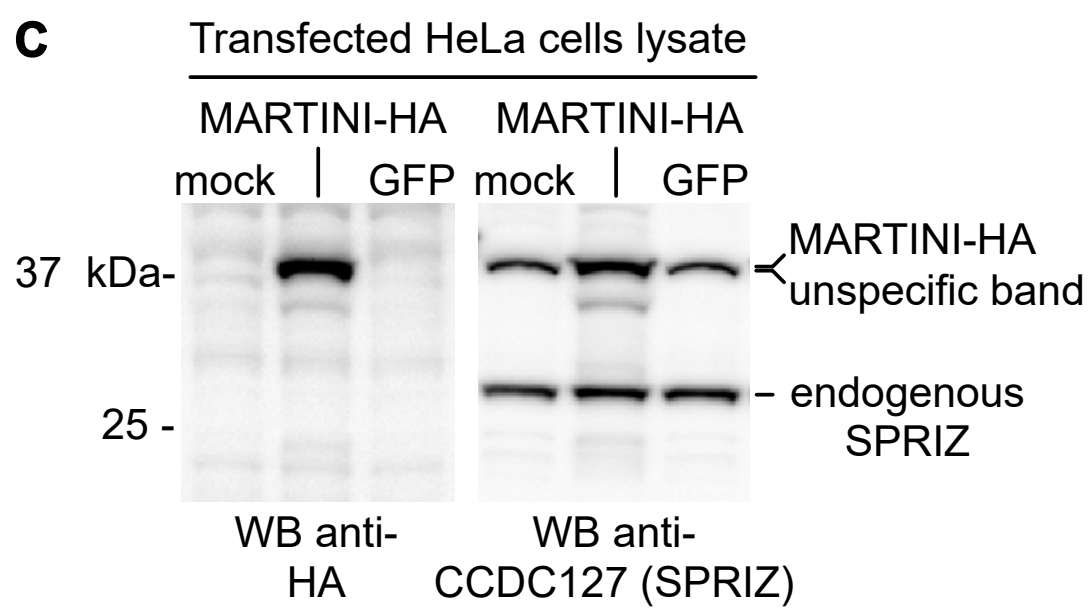

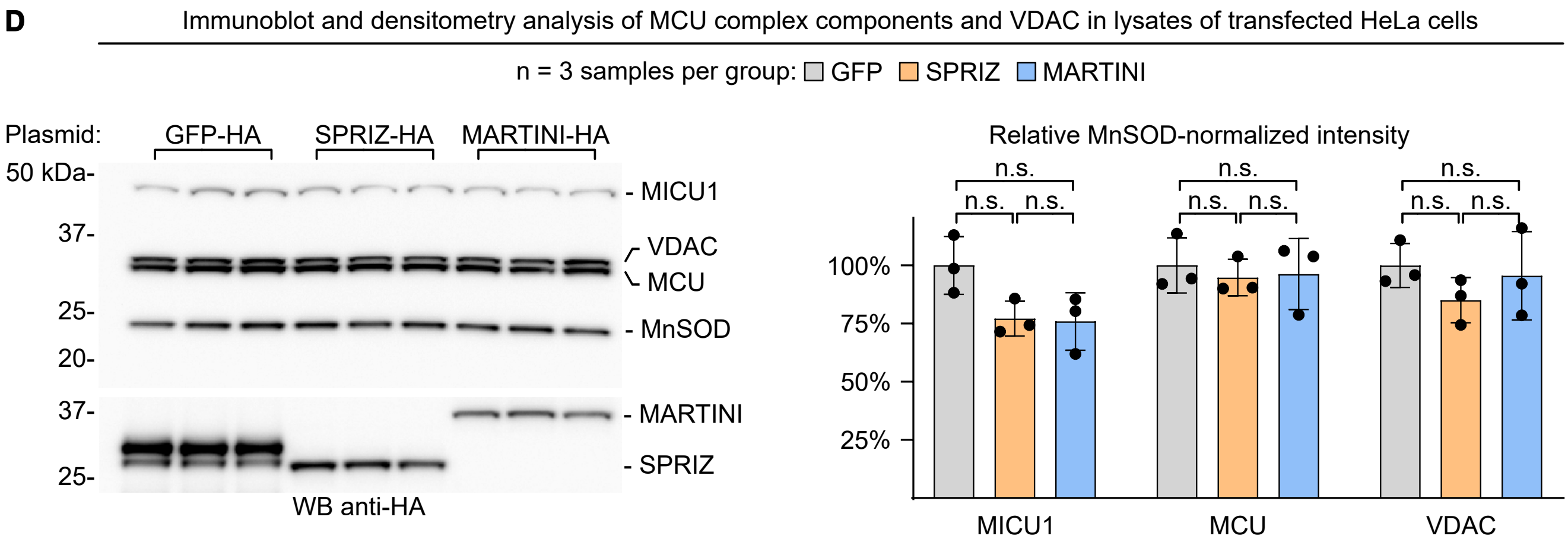

Supplementary Figure 11

Ca²⁺ imaging, MCU complex abundance, and MAM contact analysis in SPRIZ- and MARTINI-expressing HeLa cells

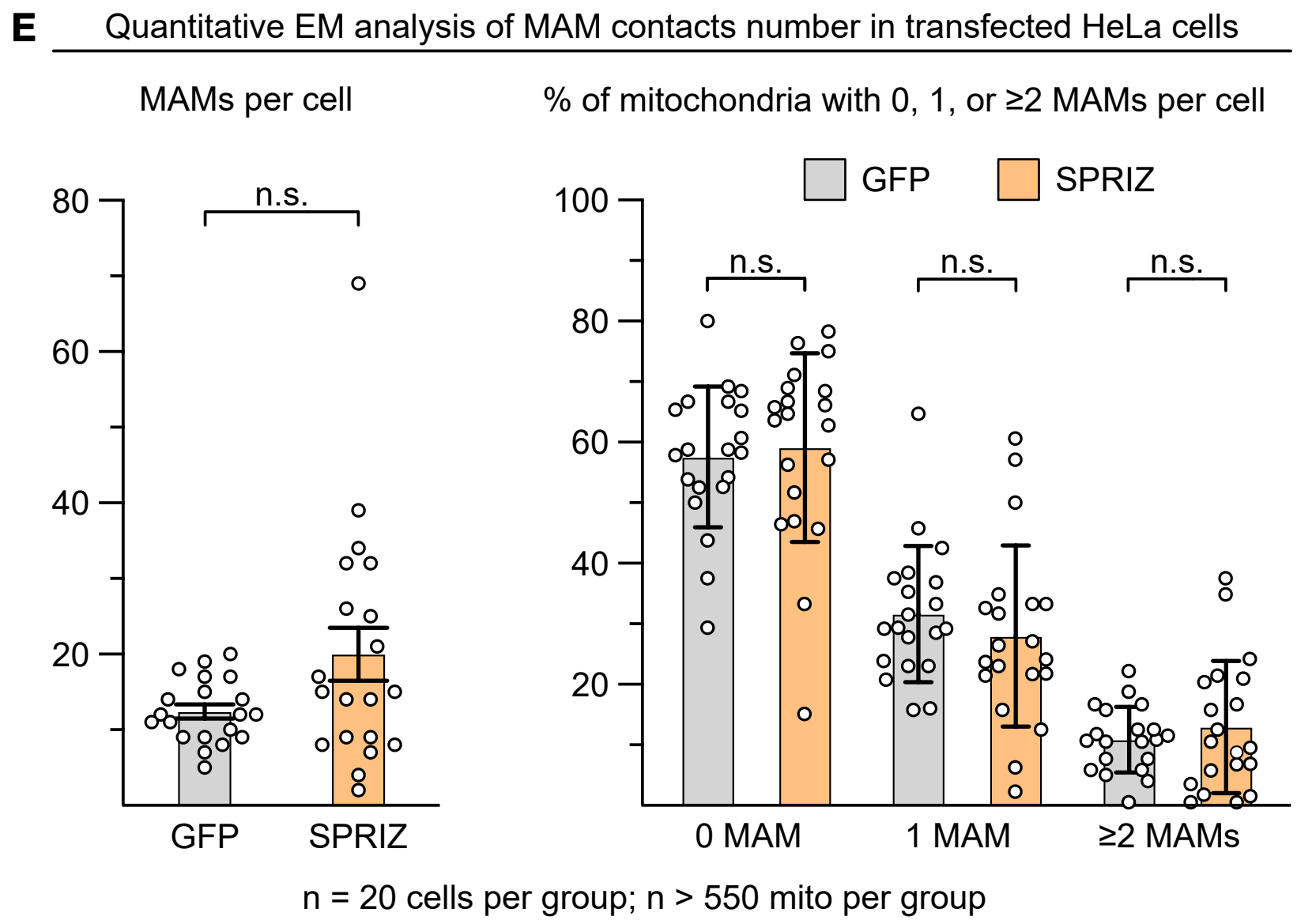

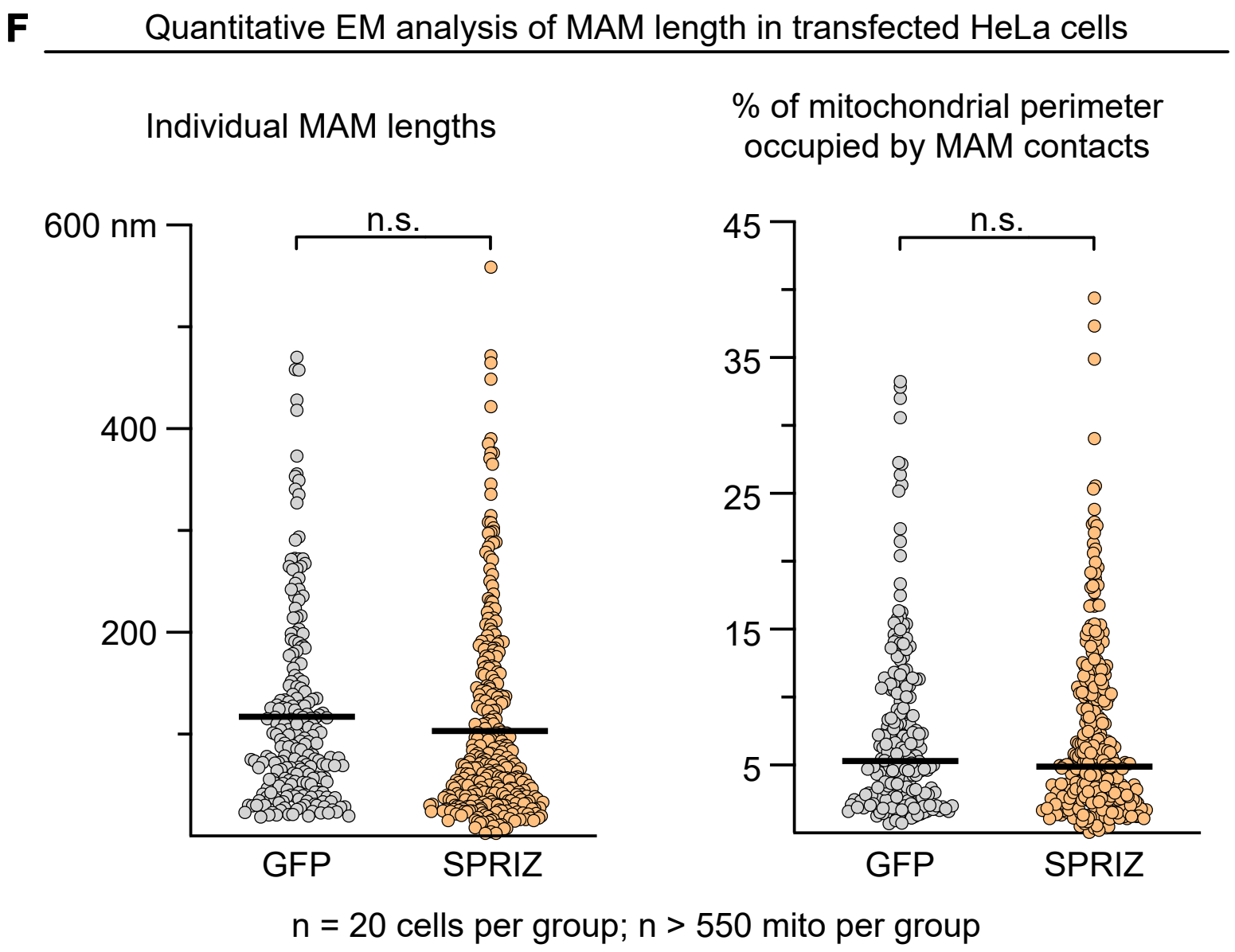

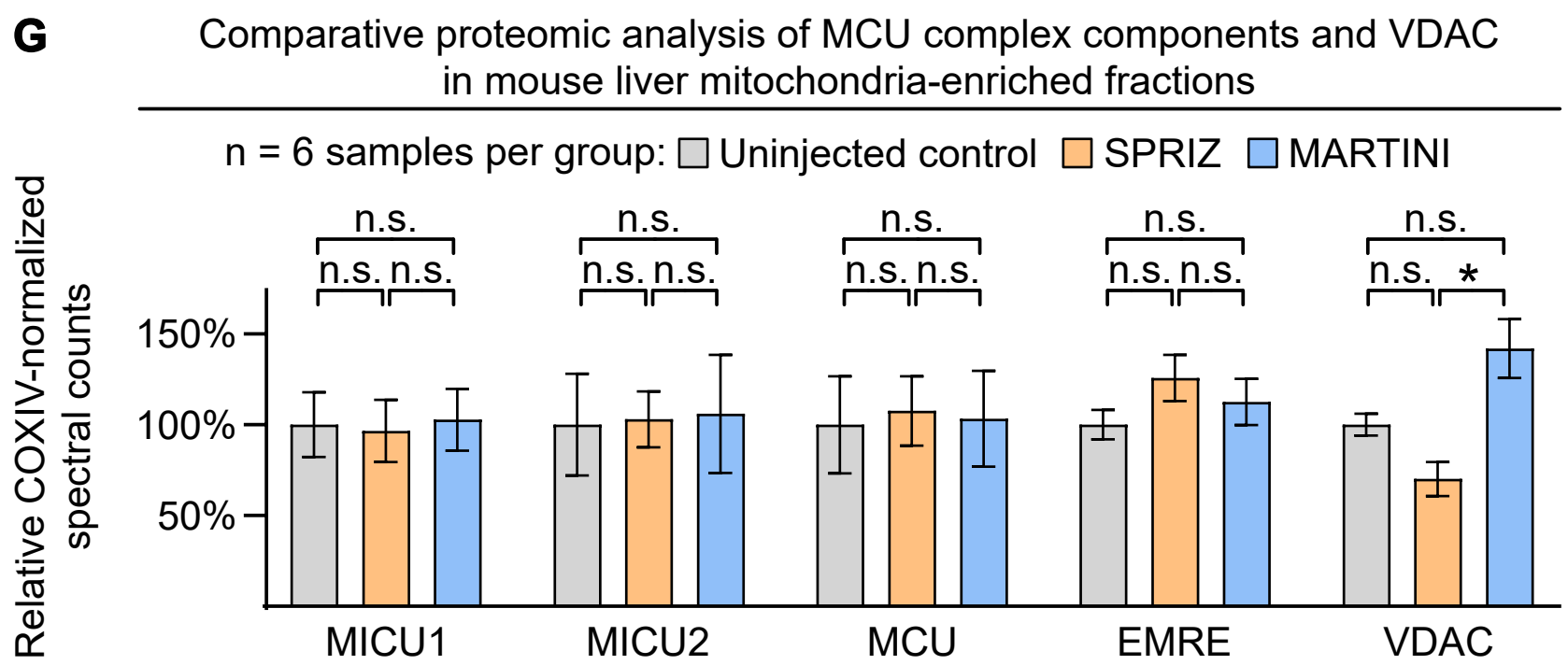

Fig. S11. Ca^{2+} imaging, MCU complex abundance, and MAM contact analysis in SPRIZ- and MARTINI-expressing HeLa cells.

(A) Cytosolic (left) and mitochondrial (middle) Ca^{2+} responses to store-operated calcium entry following addition of extracellular Ca^{2+} (500 µM). Mean traces are shown for control (gray), SPRIZ (orange), and MARTINI (blue). Insets show peak responses (mean ± SEM). Right: cytosol-versus-mitochondria phase plot showing mitochondrial Ca^{2+} signal as a function of cytosolic Ca^{2+} elevation. Peak-response quantification and pairwise comparisons are indicated in the insets (n = 3 independent biological replicates; >50 cells quantified per replicate; *, $p < 0.05$ by one-way ANOVA with Tukey's multiple-comparisons test). **(B)** Time-course of SPRIZ-HA expression (48 and 96 h post-transfection). Anti-HA detects exogenous SPRIZ-HA (upper band), whereas anti-CCDC127 (SPRIZ) detects both exogenous SPRIZ-HA and endogenous SPRIZ (lower band). **(C)** Validation of MARTINI-HA expression using parallel anti-HA and anti-CCDC127 (SPRIZ) immunoblots. MARTINI-HA is readily detected and does not alter endogenous SPRIZ levels; GFP-transfected cells serve as transfection control. **(D)** Immunoblot and densitometry analysis of MCU complex components and VDAC in lysates from control GFP-, SPRIZ-, and MARTINI-transfected HeLa cells. Left: representative immunoblot showing MICU1, VDAC, MCU, MnSOD, and HA-tagged GFP, SPRIZ, or MARTINI expression. MnSOD serves as mitochondrial loading control. Right: quantification of MICU1, MCU, and VDAC band intensities normalized to MnSOD and expressed relative to GFP control. Individual protein groups and pairwise comparisons are indicated on the graph (n = 3 samples per group; n.s., not significant by two-way ANOVA with Tukey's multiple-comparisons test; data are shown as mean ± SD). **(E)** Quantitative EM analysis of mitochondria-associated membrane (MAM) contact number in GFP- and SPRIZ-transfected HeLa cells. Left: number of MAM contacts per cell. Individual dots represent values from individual cells; bars show mean ± SEM. Right: percentage of mitochondria with 0, 1, or ≥2 MAM contacts per cell. Individual dots represent values from individual cells; bars show mean ± SEM (n = 20 cells per group; >550 mitochondria analyzed per group; n.s., not significant by unpaired t-test for MAMs per cell and two-way ANOVA with Sidák's multiple-comparisons test for MAM distribution). **(F)** Quantitative EM analysis of MAM contact length in GFP- and SPRIZ-transfected HeLa cells. Left: individual MAM contact lengths. Right: percentage of mitochondrial perimeter occupied by MAM contacts. Individual dots represent individual MAM contacts or mitochondria; horizontal bars indicate the mean (n = 20 cells per group; >550 mitochondria analyzed per group; n.s., not significant by unpaired t-test). **(G)** Comparative proteomic analysis of MCU complex components and VDAC in mouse liver mitochondria-enriched fractions isolated from uninjected control, SPRIZ-expressing, and MARTINI-expressing mice. Quantification shows COXIV-normalized spectral counts for MICU1, MICU2, MCU, EMRE, and VDAC, expressed relative to uninjected control. Individual protein groups and pairwise comparisons are indicated on the graph (n = 6 samples per group; *, $p < 0.05$, n.s., not significant by two-way ANOVA with Tukey's multiple-comparisons test; data are shown as mean ± SEM).

Supplementary Figure 12

Ultrastructural analyses of SPRIZ- and MARTINI-expressing mouse liver

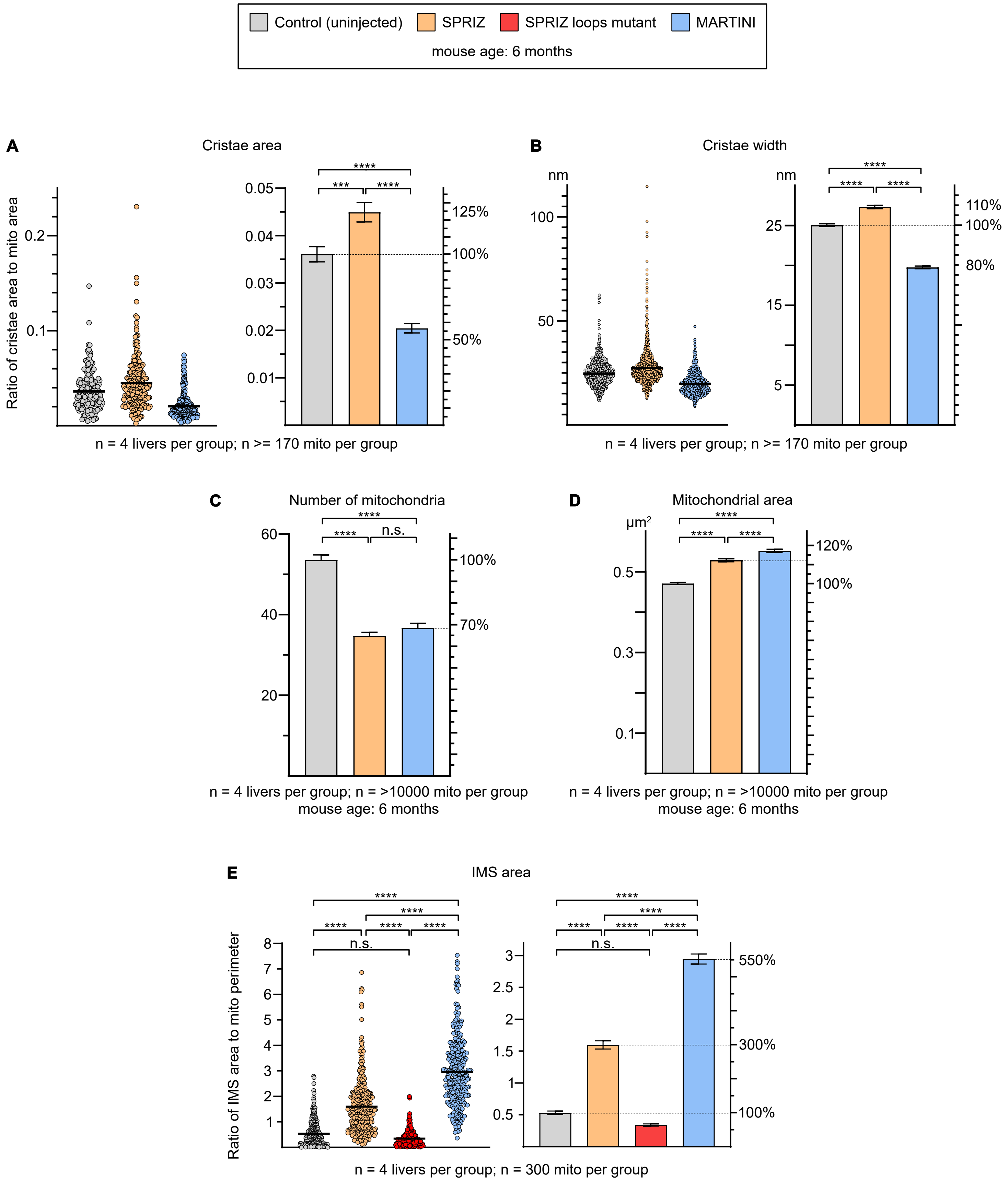

Supplementary Figure 12

Ultrastructural analyses of SPRIZ- and MARTINI-expressing mouse liver

G 3D reconstruction of the outer ( ) and inner ( ) boundary membranes from electron tomography analysis

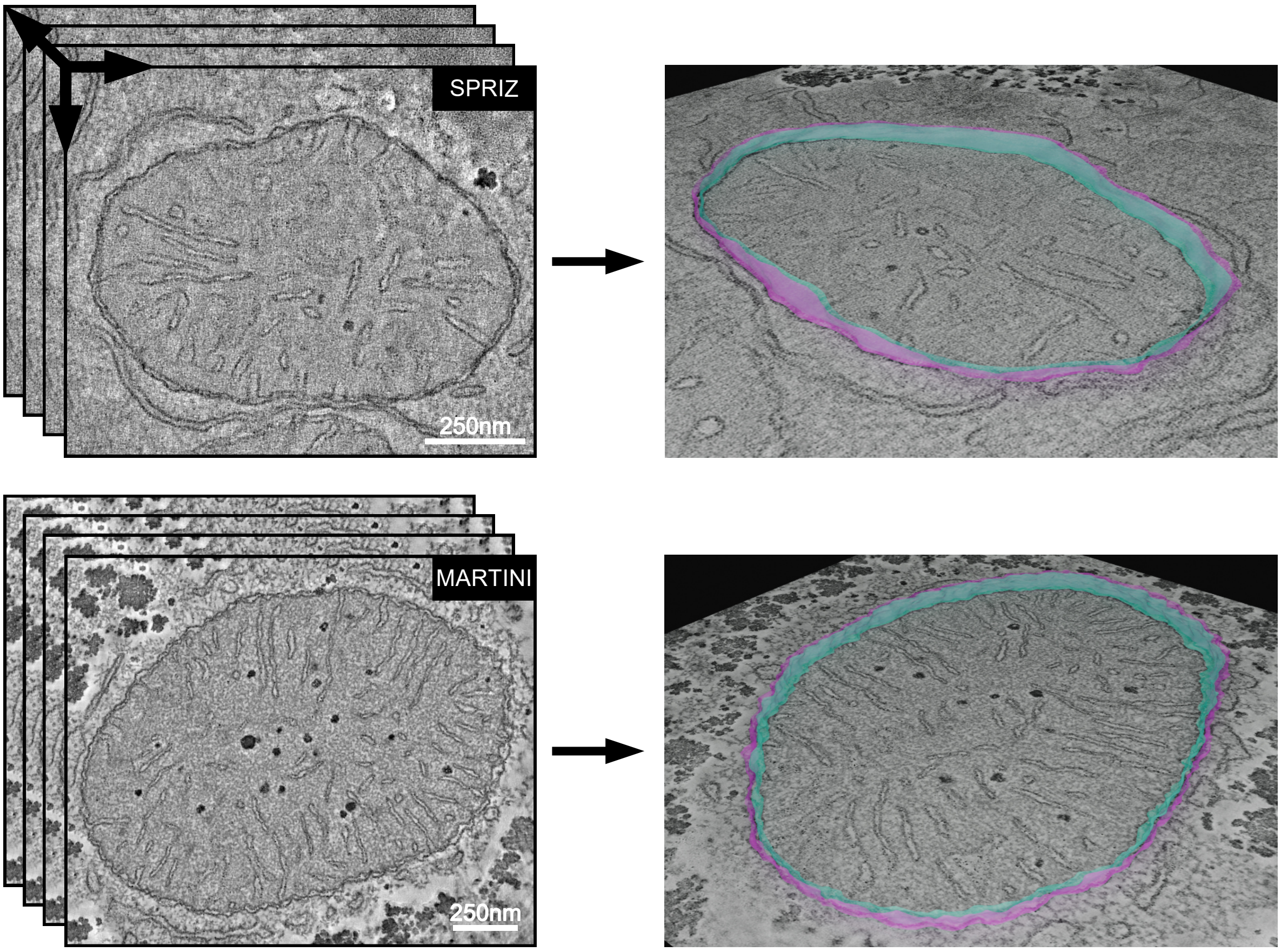

H SPRIZ- and MARTINI-induced mitochondrial membrane undulations by volume EM

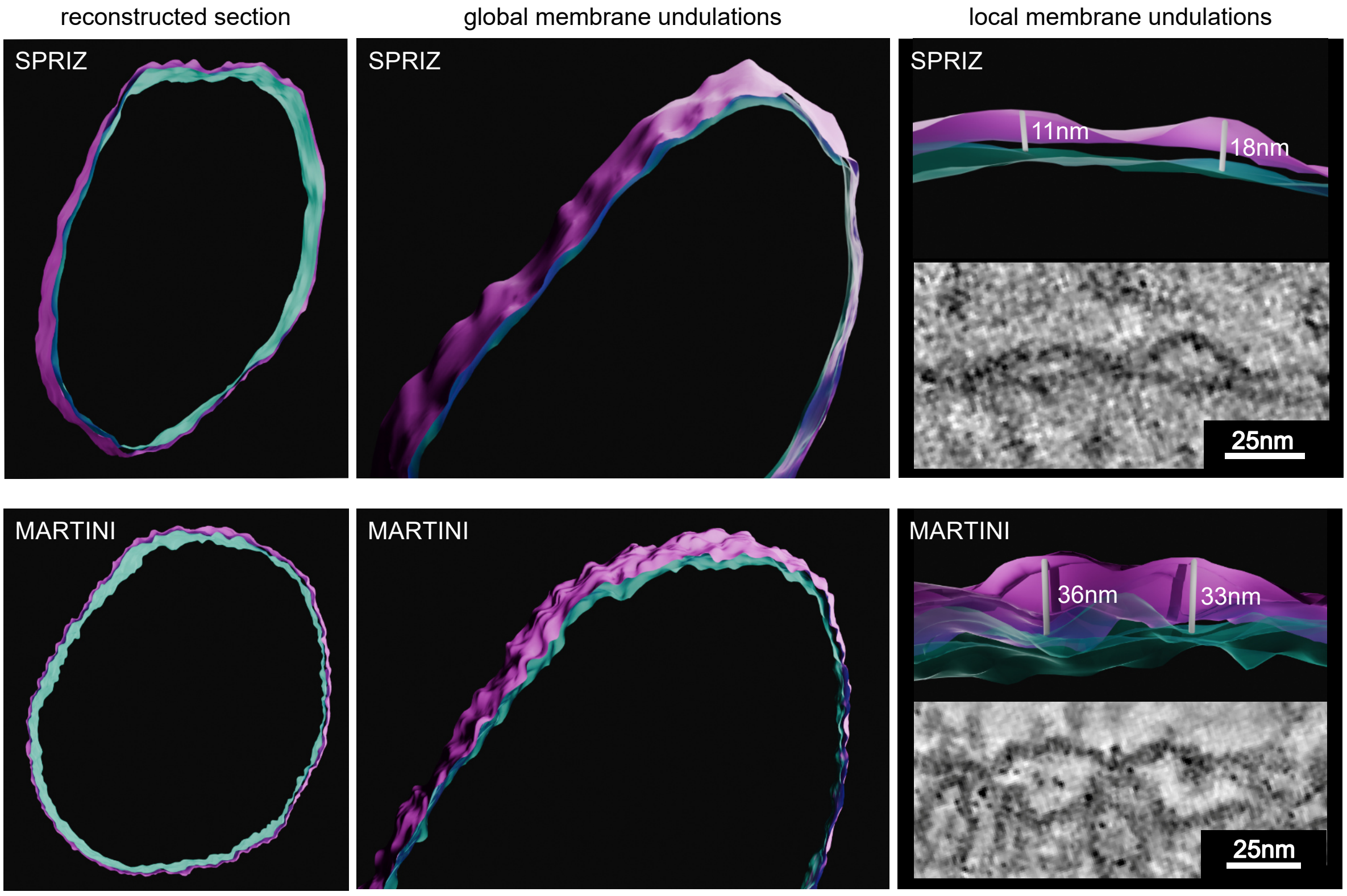

Supplementary Figure 12

Ultrastructural analyses of SPRIZ- and MARTINI-expressing mouse liver

F Representative mouse liver mitochondria

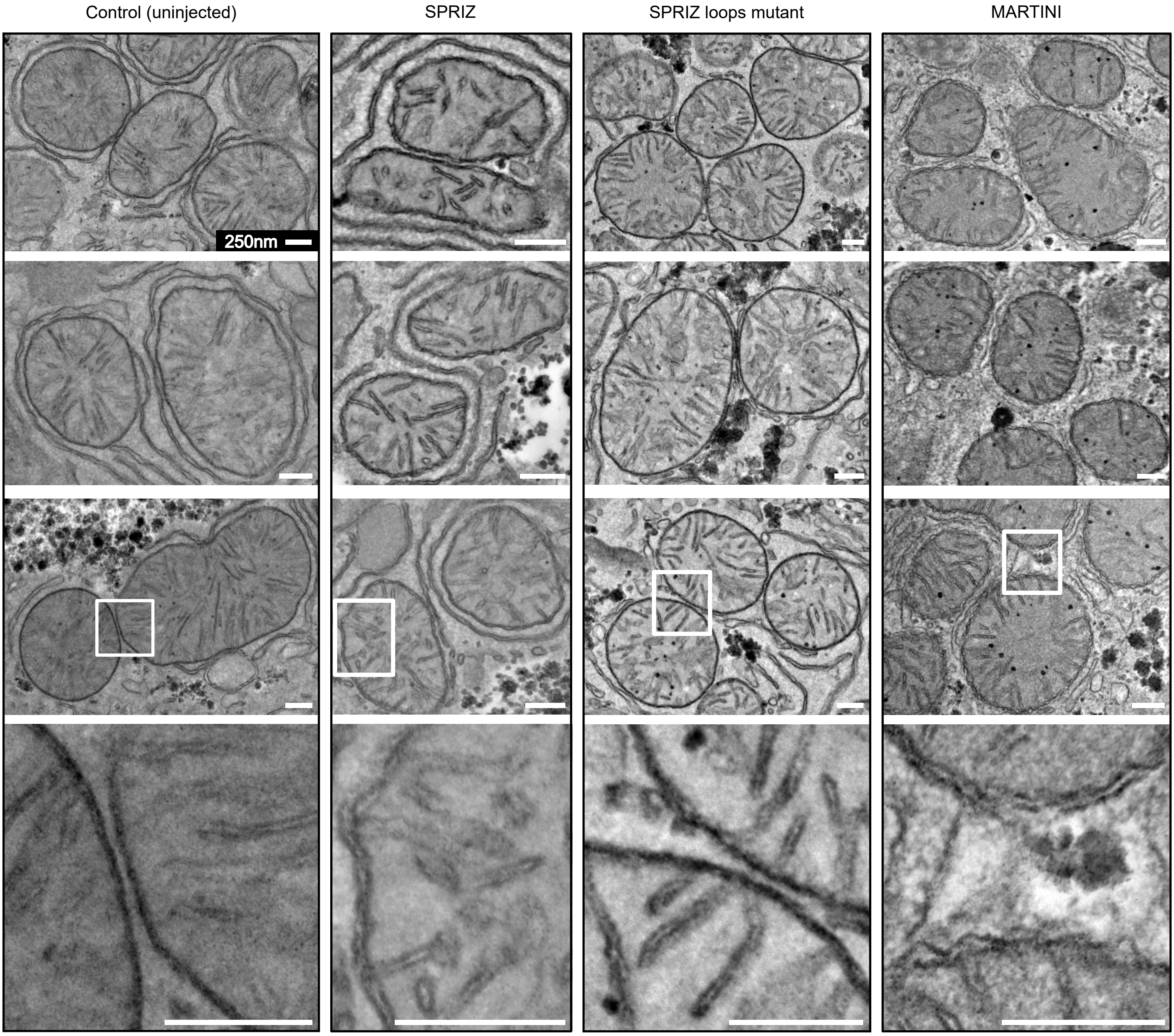

Supplementary Figure 12

Ultrastructural analyses of SPRIZ- and MARTINI-expressing mouse liver

I Workflow used to quantify the OMM–IMM distance by 3D-ET

Step 1: Segmentation and reconstruction
of the mitochondrial boundary membranes from ET dataset

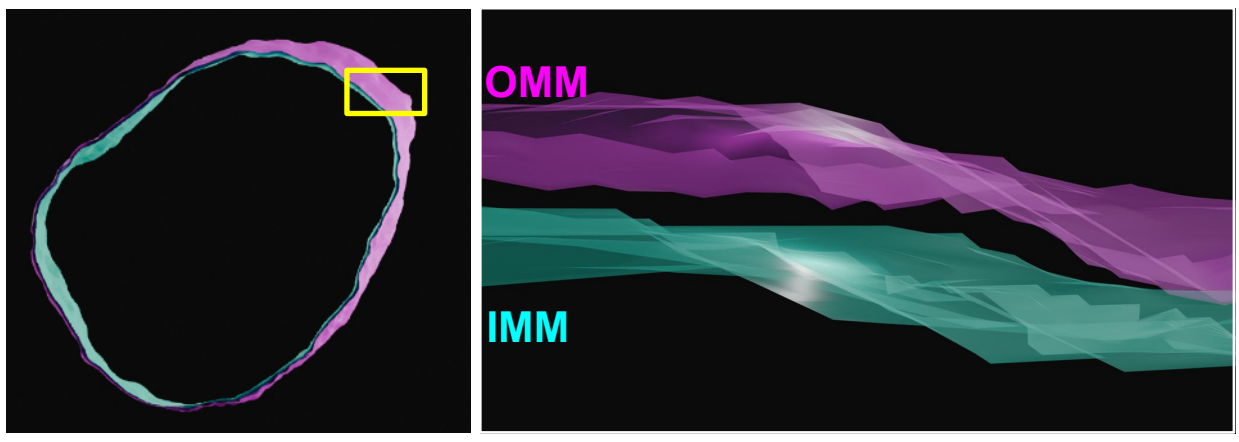

Low magnification High magnification

Step 2: Measurement of the shortest 3D distance
from each OMM voxel to its nearest IMM voxel

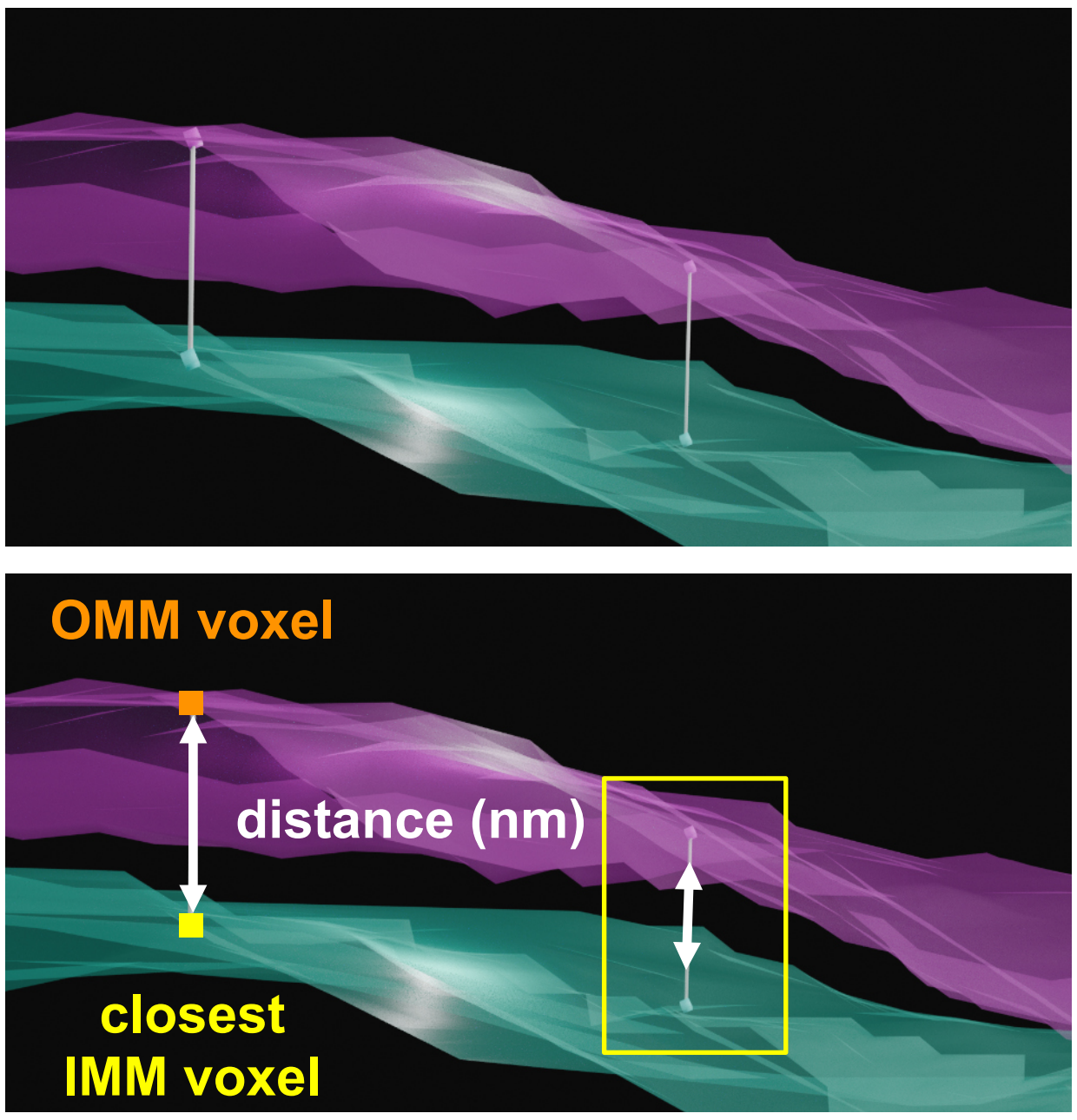

Step 3: Global view of OMM–IMM voxels-distance measurements

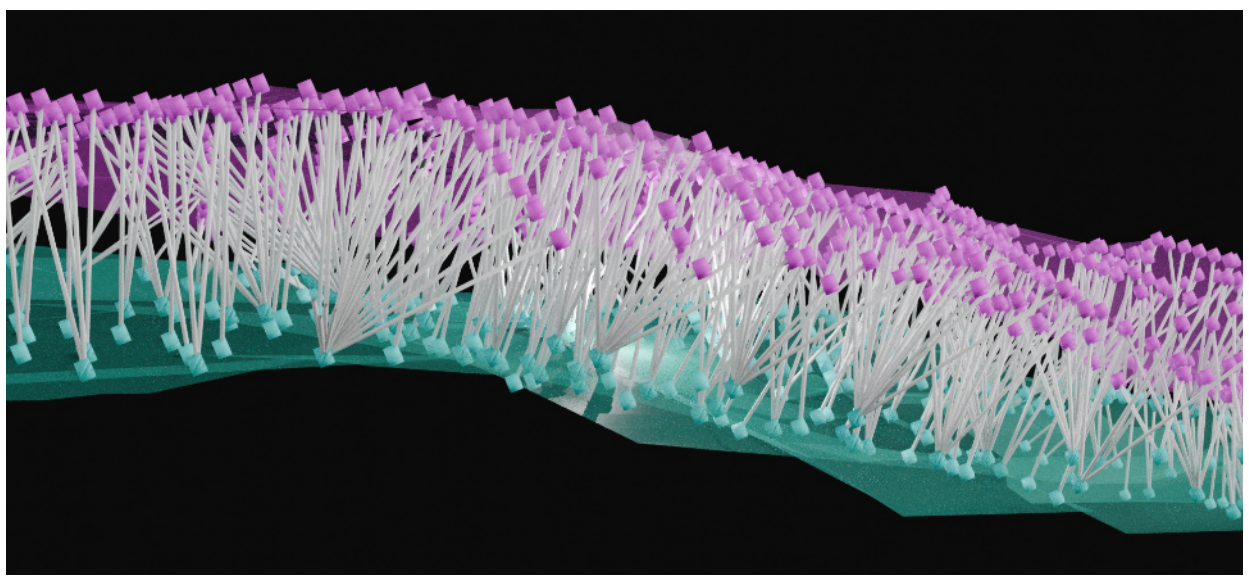

Representative map of voxel-distance measurements

Step 4: Statistical analysis voxel-distance distributions

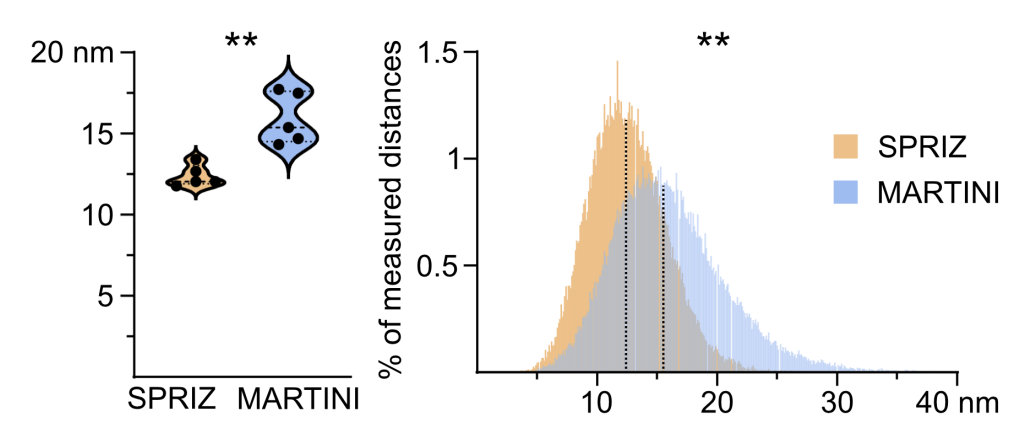

Figure 6F

Fig. S12. Ultrastructural analyses of SPRIZ- and MARTINI-expressing mouse liver.

(A) *Cristae* area (ratio of *cristae* area to mitochondrial area). Data are shown as individual values (left) or mean ± SEM (right). Workflow in fig. S6D. $n = 4$ livers per group; ≥170 mitochondria per group. ***, $p < 0.001$, ****, $p < 0.0001$ by one-way ANOVA with Tukey's multiple-comparisons test. **(B)** *Cristae* width. Data are shown as individual values (left) or mean ± SEM (right). $n = 4$ livers per group; 170 mitochondria per group. ****, $p < 0.0001$ by one-way ANOVA with Tukey's multiple-comparisons test. **(C)** Mitochondrial number (per 225 μm^2 EM field). $n = 4$ livers per group; >10,000 mitochondria quantified per group. ****, $p < 0.0001$ by one-way ANOVA with Tukey's multiple-comparisons test; mean ± SEM. **(D)** Mean cross-sectional area (μm^2). $n = 4$ livers per group; >10,000 mitochondria quantified per group. n.s., not significant; ****, $p < 0.0001$ by one-way ANOVA with Tukey's multiple-comparisons test; mean ± SEM. **(E)** IMS area expansion (ratio of IMS area to mitochondrial perimeter). Data are shown as individual values (left) or mean ± SEM (right). Workflow in fig. S6E. $n = 4$ livers per group; 300 mitochondria per group. ****, $p < 0.0001$ by one-way ANOVA with Tukey's multiple-comparisons test. **(F)** Representative transmission electron micrographs of hepatocytes from uninjected control mouse livers and from AAV8-injected mouse livers expressing SPRIZ, SPRIZ loops mutant, or MARTINI, showing mitochondrial ultrastructure. Multiple fields are shown for each condition; scale bars are indicated on the images. **(G)** 3D-ET reconstruction of outer and inner boundary membranes. Representative ET serial sections from liver mitochondria of SPRIZ- and MARTINI-expressing mice (left). Corresponding 3D reconstructions (right) show outer membrane in magenta and inner boundary membrane in cyan. Scale bars, 250 nm. **(H)** SPRIZ- and MARTINI-induced mitochondrial membranes undulations revealed by 3D-ET; scale bars, 25 nm. **(I)** Workflow used to quantify OMM–IMM distance by 3D-ET. Step 1: segmentation and 3D reconstruction of the mitochondrial boundary membranes from ET datasets, with OMM and IMM boundaries shown in magenta and cyan, respectively. Step 2: measurement of the shortest 3D distance from each OMM voxel to its nearest IMM voxel, shown for a representative example. Step 3: global view of OMM–IMM voxel-distance measurements across the reconstructed mitochondrial membrane surface, shown as a representative map of voxel-distance measurements. Step 4: statistical analysis of voxel-distance distributions, corresponding to the quantification shown in Fig. 6F.

Supplementary Figure 13

Structural and biochemical validation of the SPRIZ loops mutant

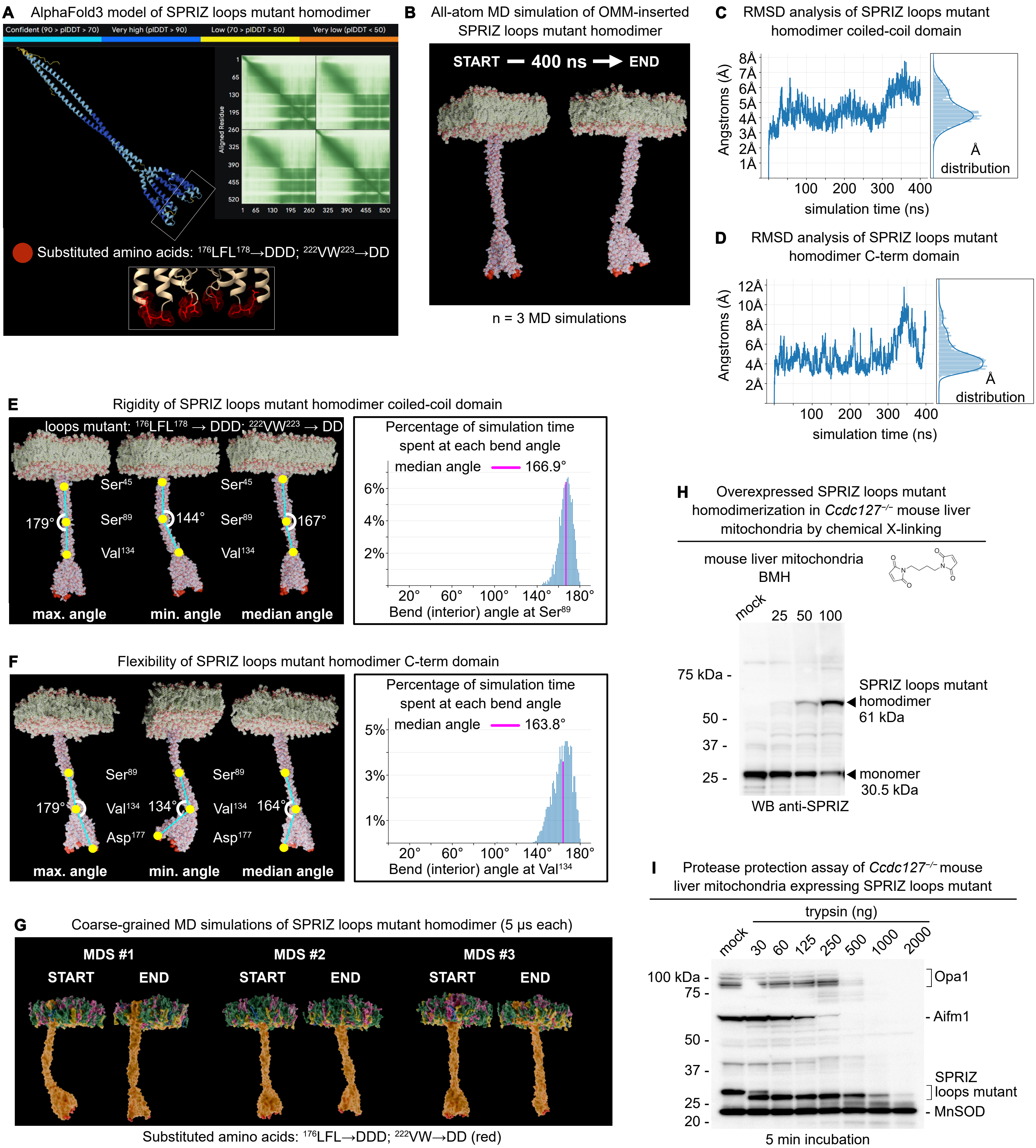

Fig. S13. Structural and biochemical validation of the SPRIZ loops mutant.

(A) AlphaFold3 model of the SPRIZ loops-mutant homodimer (^{176}LFL→^{176}DDD; ^{222}VW→^{222}DD) and predicted aligned error (PAE) map. Substituted residues are highlighted in red in the enlarged C-terminal conical domain. (B) Starting and 400-ns endpoint structures from an all-atom simulation of the OMM-anchored SPRIZ loops-mutant homodimer in an OMM-like bilayer (n = 3 independent simulations). **(C–D)** Backbone RMSD over 400 ns for the coiled-coil (C) and C-terminal conical (D) domains. Insets show RMSD distributions. **(E)** Coiled-coil domain rigidity during simulation. Snapshots show maximum (179°), minimum (144°), and median (167°) bend angles at Ser^{89}; Ser^{45}, Ser^{89}, and Val^{134} are marked, and yellow lines indicate the helical axis. The angle distribution has a median of 166.9°, indicating a near-linear coiled-coil with limited bending. **(F)** Flexibility of the C-terminal conical domain quantified using bend angles defined by Ser^{89}, Val^{134}, and Asp^{177}. Snapshots show maximum (179°), minimum (134°), and median (164°) bend angles at Val^{134}. The angle distribution has a median of 163.8°. **(G)** Three independent 5-µs coarse-grained MD simulations of the OMM-anchored SPRIZ loops-mutant homodimer, showing preservation of the homodimer architecture throughout the trajectories. Substituted residues are shown in red. **(H)** SPRIZ loops-mutant homodimerization by BMH crosslinking of mitochondria-enriched fractions from *Ccdc127*$^{-/-}$ mouse livers expressing the SPRIZ loops mutant. BMH (0 (mock), 25, 50, or 100 µM) produced a prominent ~61 kDa SPRIZ loops-mutant band (arrowhead), with the ~30.5 kDa monomer indicated. **(I)** Protease-protection assay confirming SPRIZ loops-mutant topology in *Ccdc127*$^{-/-}$ mouse liver mitochondria. Mitochondria expressing the SPRIZ loops mutant were treated with trypsin. The SPRIZ loops mutant is protected at low concentrations, like IMS/IMM markers Opa1 and Aifm1, and progressively lost at higher doses, consistent with an N-cytosolic, IMS-facing orientation. MnSOD is shown as a matrix marker.

Supplementary Figure 14

Functional analysis of SPRIZ loops mutant-overexpressing and *Ccdc127* $^{-/-}$ mouse liver mitochondria

A Ca²⁺ retention capacity (CRC) of mouse liver mitochondria-enriched fractions

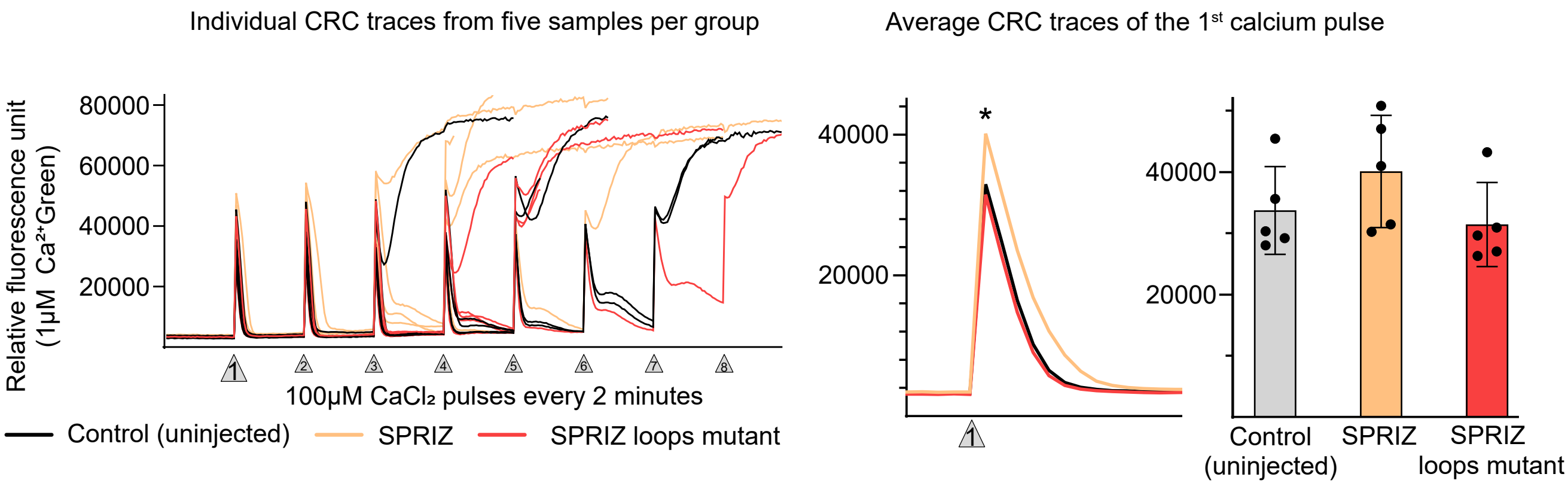

B Swelling assay of mouse liver mitochondria-enriched fractions

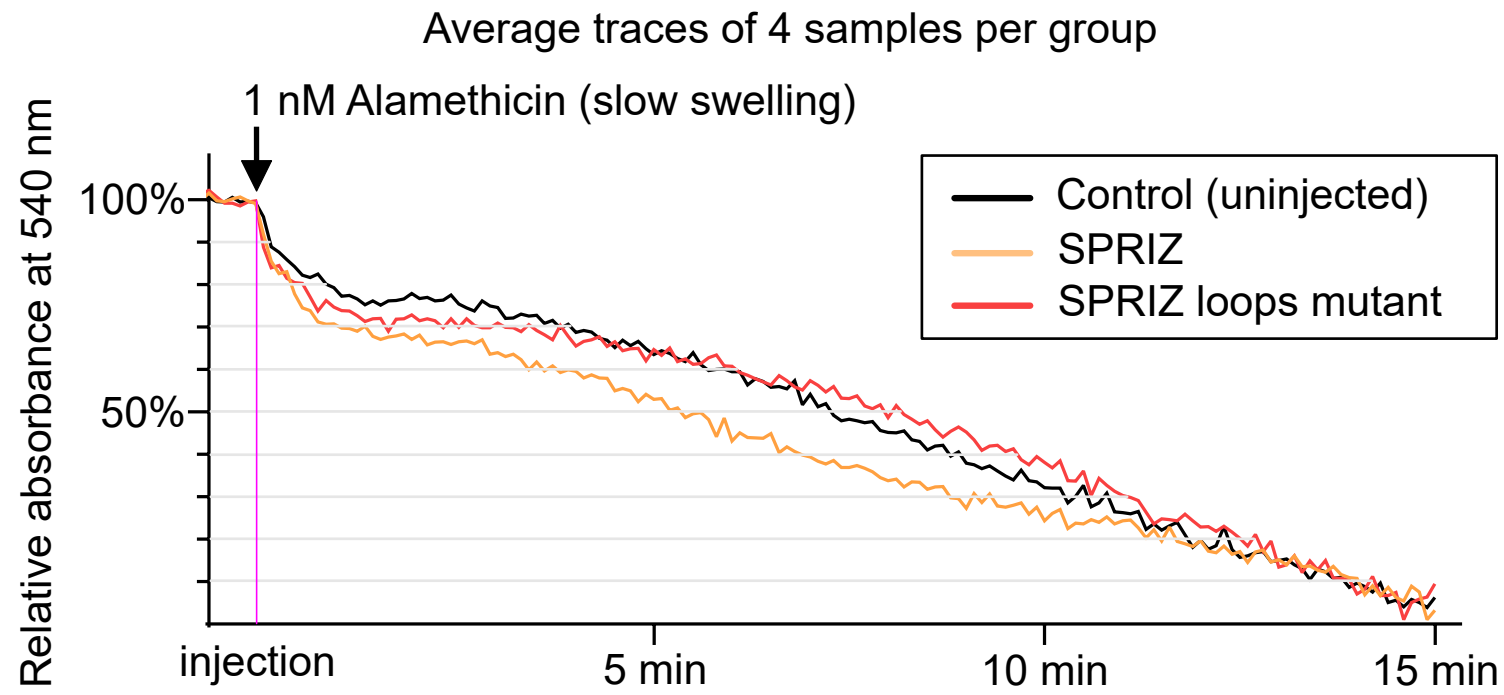

C Ca²⁺ retention capacity (CRC) of mouse liver mitochondria-enriched fractions

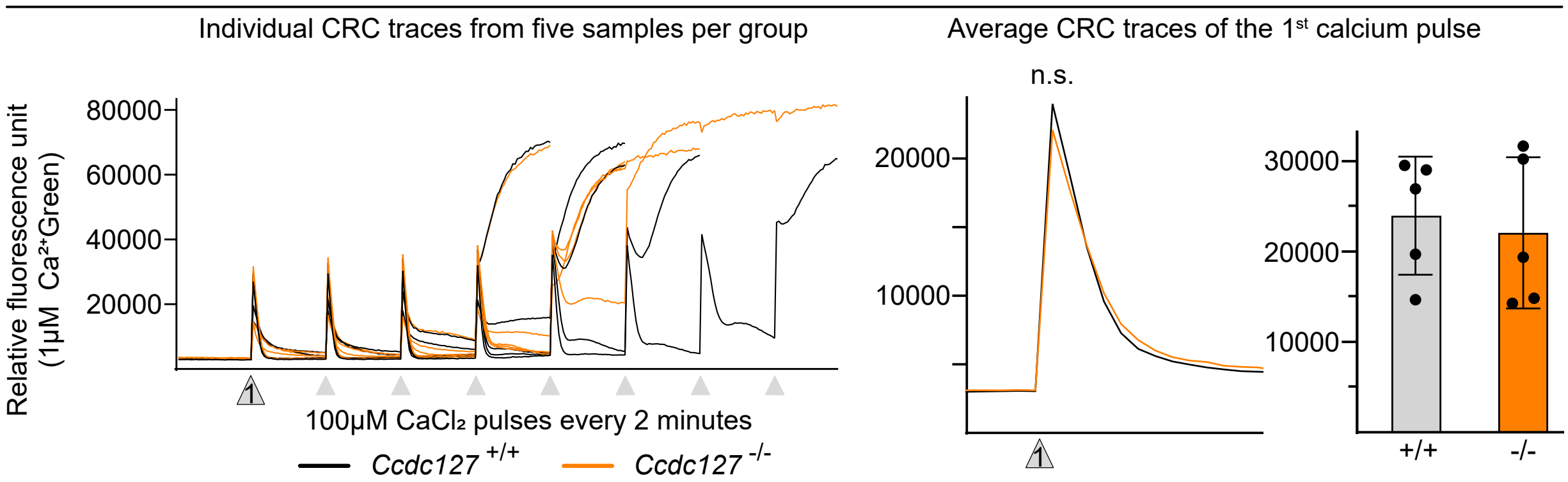

D Swelling assay of mouse liver mitochondria-enriched fractions

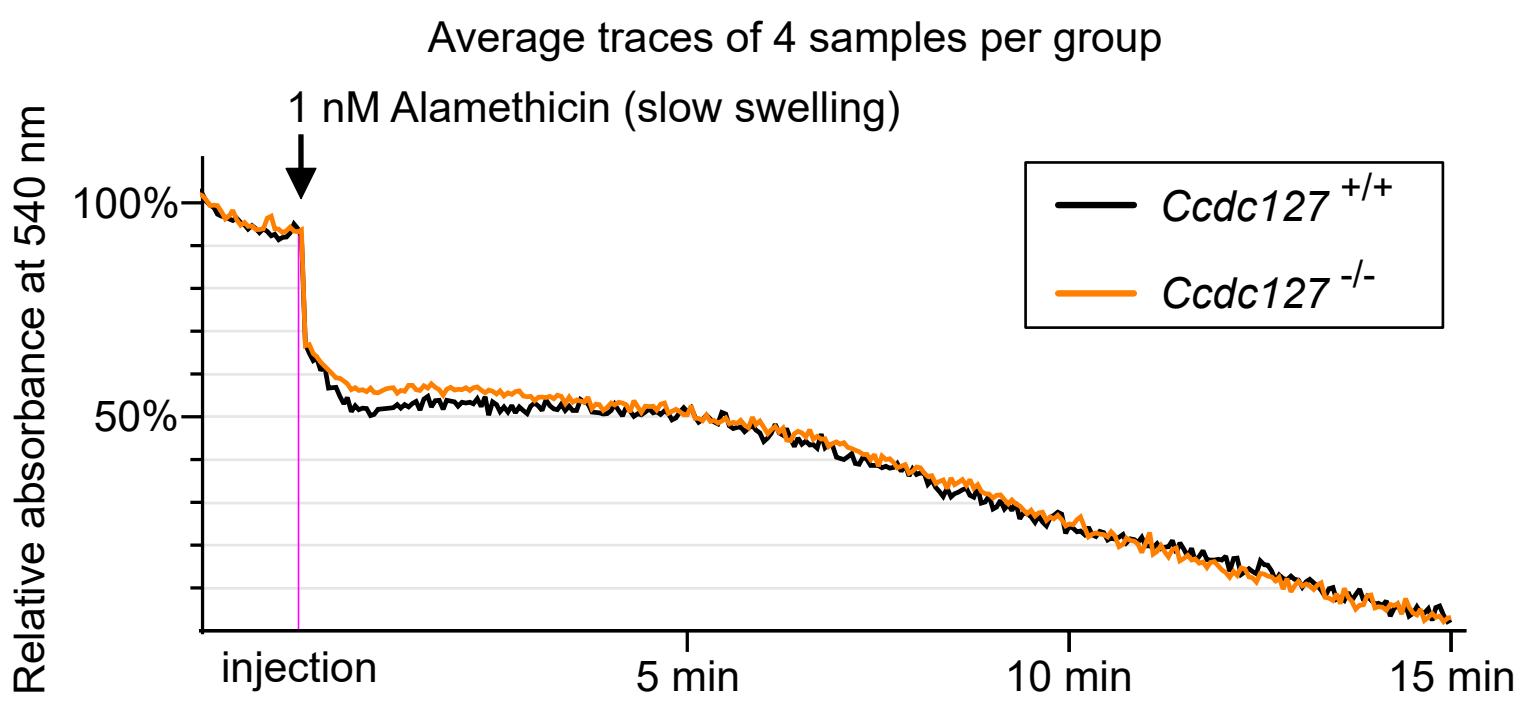

Supplementary Figure 14

Functional analysis of SPRIZ loops mutant-overexpressing and *Ccdc127* $^{-/-}$ mouse liver mitochondria

E Ca^{2+}-induced swelling assay of mouse liver mitochondria-enriched fractions

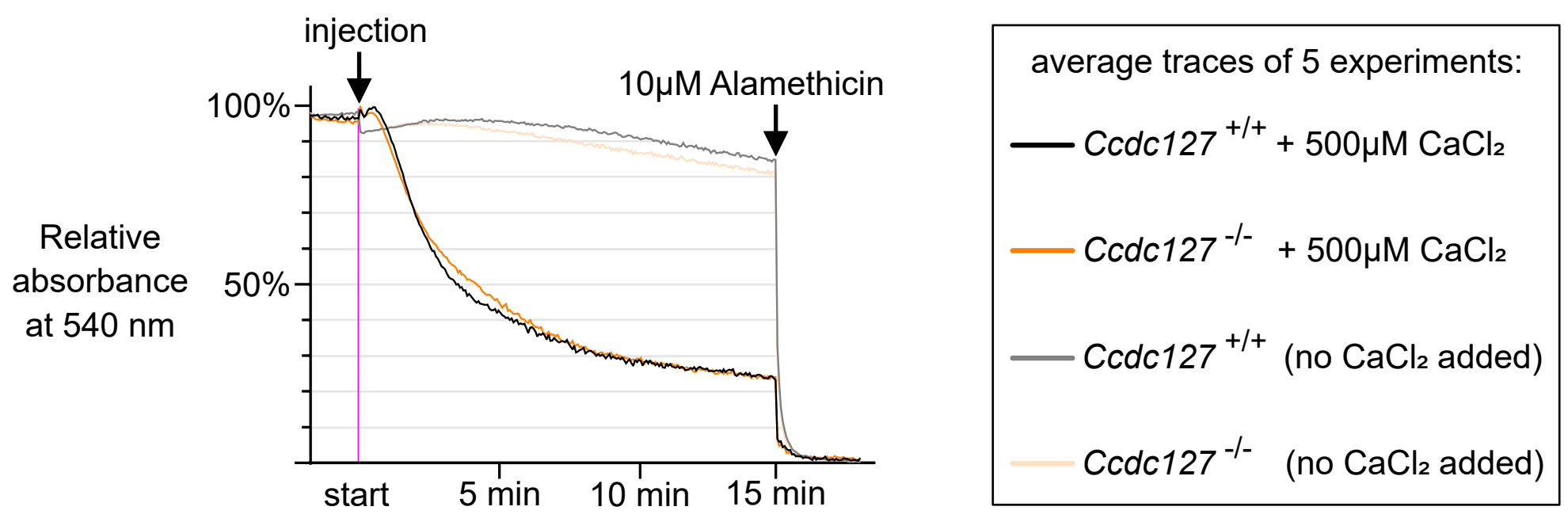

Fig. S14. Functional analysis of SPRIZ loops mutant-overexpressing and *Ccdc127*$^{-/-}$ mouse liver mitochondria.

(A) Ca^{2+} retention capacity (CRC) of mouse liver mitochondria-enriched fractions from uninjected control, SPRIZ-expressing, and SPRIZ loops mutant-expressing mice. Left: individual CRC traces from five biological samples per group. Sequential 100 µM $CaCl_2$ pulses were added every 2 min while monitoring extramitochondrial Ca^{2+} with Ca^{2+} Green. Middle: average CRC traces for the first Ca^{2+} pulse. Right: quantification of the first Ca^{2+} pulse. Individual dots represent biological replicates; bars show mean ± SD (n = 5 samples per group; *, $p < 0.05$ by Friedman test). **(B)** Alamethicin-induced swelling assay in liver mitochondria-enriched fractions. Mitochondrial swelling was monitored as decrease in absorbance at 540 nm. Average traces of four biological samples per group are shown for control (black), SPRIZ (orange), and SPRIZ loops mutant (red) groups. 1 nM alamethicin (slow swelling) was used to assess swelling kinetics. Arrow indicates alamethicin addition. **(C)** Ca^{2+} retention capacity (CRC) of mouse liver mitochondria-enriched fractions from *Ccdc127*$^{+/+}$ and *Ccdc127*$^{-/-}$ mice. Left: individual CRC traces from five biological samples per group. Sequential 100 µM $CaCl_2$ pulses were added every 2 min while monitoring extramitochondrial Ca^{2+} with Ca^{2+} Green. Middle: average CRC traces for the first Ca^{2+} pulse. Right: quantification of the first Ca^{2+} pulse. Individual dots represent biological replicates; bars show mean ± SD (n = 5 samples per group; n.s., not significant by Wilcoxon test). **(D)** Alamethicin-induced swelling assay in liver mitochondria-enriched fractions from *Ccdc127*$^{+/+}$ and *Ccdc127*$^{-/-}$ mice. Mitochondrial swelling was monitored as decrease in absorbance at 540 nm. Average traces of four biological samples per group are shown for *Ccdc127*$^{+/+}$ (black) and *Ccdc127*$^{-/-}$ (dark orange) groups. 1 nM alamethicin (slow swelling) was used to assess swelling kinetics. Arrow indicates alamethicin addition. **(E)** Ca^{2+}-induced swelling assay in liver mitochondria-enriched fractions from *Ccdc127*$^{+/+}$ and *Ccdc127*$^{-/-}$ mice. Mitochondrial swelling was monitored as decrease in absorbance at 540 nm following addition of 500 µM $CaCl_2$. Average traces of five biological samples per group are shown for *Ccdc127*$^{+/+}$ and *Ccdc127*$^{-/-}$ mitochondria with or without $CaCl_2$ addition. Alamethicin was added at 10 µM at the end of the assay as a maximal swelling control. Arrows indicate $CaCl_2$ and alamethicin addition.

Supplementary Figure 15

Preserved mitochondrial respiration in SPRIZ- and MARTINI-expressing mouse liver

A High-resolution respirometry in mouse liver mitochondria-enriched fraction

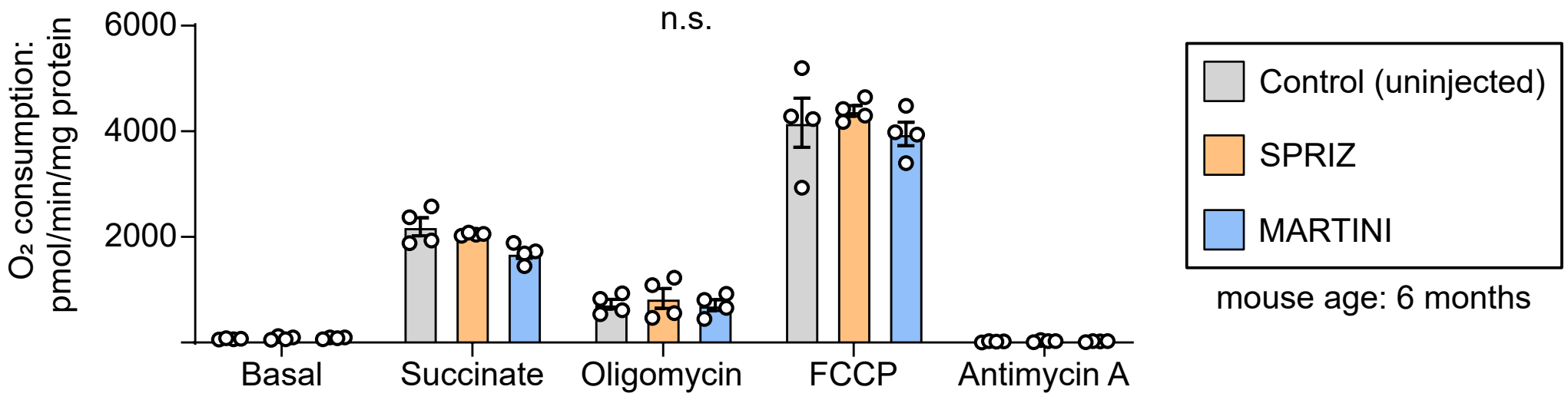

B Fatty acid oxidation assay in mouse liver mitochondria

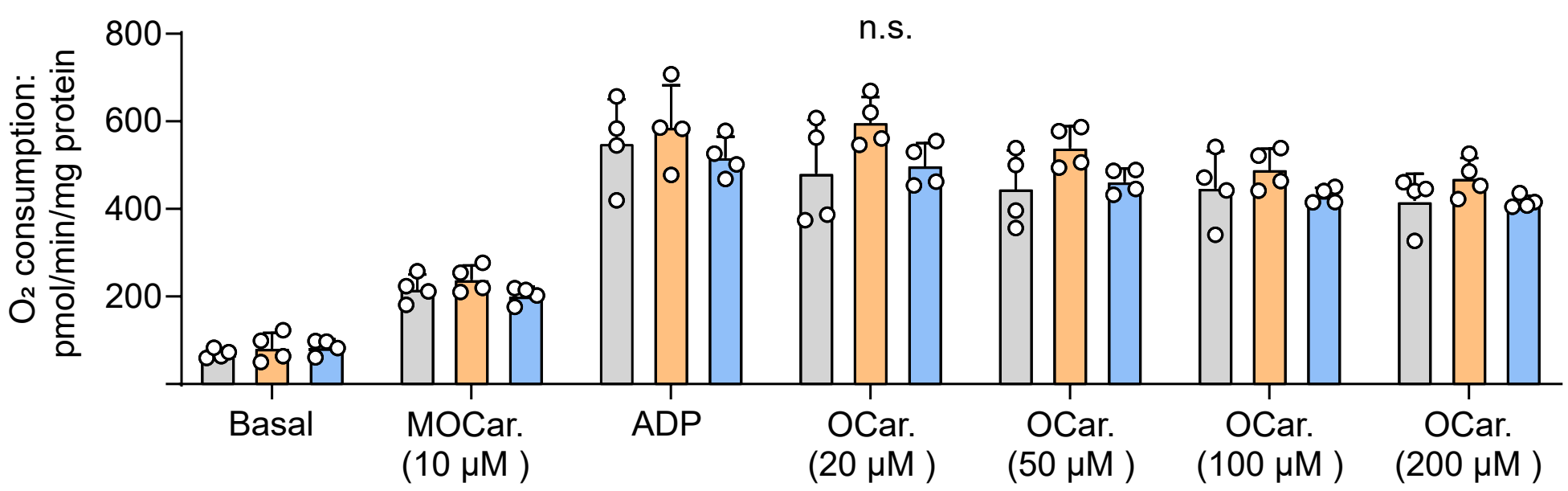

Fig. S15. Preserved mitochondrial respiration in SPRIZ- and MARTINI-expressing mouse liver.

(A) High-resolution respirometry under succinate-supported conditions in liver mitochondria-enriched fractions. Oxygen consumption rates across basal, succinate, oligomycin, FCCP, and antimycin A states. Individual biological replicates (dots) with mean ± SD. n.s., not significant by two-way ANOVA with Tukey's multiple-comparisons test. **(B)** Fatty-acid oxidation respirometry in liver mitochondria-enriched fractions with sequential octanoylcarnitine (OCar) additions at the indicated concentrations. Individual biological replicates (dots) with mean ± SD. n.s., not significant by two-way ANOVA with Tukey's multiple-comparisons test.

Supplementary Table 1

Representative invertebrate SPRIZ (CCDC127) protein homologues identified in Clustered_NR, RefSeq, and UniProt databases

Species	Accession	Common name
Saccoglossus kowalevskii	A0ABM0MXP1	Virginia acorn worm
Ptychodera flava	XP_070534857.1	Yellow acorn worm
Priapulus caudatus	A0ABM1E9R8	Cactus worm
Lingula anatina	A0A1S3JPL2	Lamp shell (brachiopod)
Flustra foliacea	CAN6025435.1	Hornwrack (bryozoan)
Branchiostoma belcheri	LOC109463890	Belcher's lancelet
Branchiostoma floridae	C3ZCF1	Florida lancelet
Branchiostoma lanceolatum	A0A8J9YZU9	European lancelet
Asterias rubens	XP_033640674.1	European starfish
Anneissia japonica	XP_033109687.1	Japanese feather starfish
Strongylocentrotus purpuratus	A0A7M7G3G2	Purple sea urchin
Diadema antillarum	XP_071498913.1	Long-spined sea urchin
Diadema setosum	XP_072176051.1	Long-spined black sea urchin
Limulus polyphemus	XP_013788901.1	Atlantic horseshoe crab
Centruroides vittatus	XP_067123193.1	Striped bark scorpion
Centruroides sculpturatus	XP_023238890.1	Arizona bark scorpion

Table S1. Representative invertebrate SPRIZ (CCDC127) orthologues identified in Clustered_NR, RefSeq, and UniProt databases.

Representative invertebrate SPRIZ orthologues detected by systematic database mining of Clustered_NR, RefSeq, and UniProt are listed with accession numbers and common names. The complete dataset comprises 16 sequences spanning multiple bilaterian lineages including hemichordates, priapulids, brachiopods, bryozoans, cephalochordates, echinoderms, and chelicerate arthropods, illustrating the sporadic distribution of SPRIZ outside vertebrates.

Supplementary Table 2. Comparative transcript and protein abundance of SPRIZ in mouse liver and HEK293 cells

Gene	Name	Mouse liver: Number of transcripts (TPM) from Anastasia et al (21)	Mouse liver: Average iBAQ value from LC–MS/MS analyses of wrappER-associated mitochondria-enriched fractions (n = 5; this study)	HEK293 cells: Number of proteins per single cell from Morgenstern et al (40)
Oma1	OMA1 metalloendopeptidase	9	554	16,123
Ambra1	Activating molecule in BECN1-regulated autophagy	10	NA	NA
Pdzd8	PDZ domain-containing protein 8	11	309	NA
Mcu	Mitochondrial calcium uniporter	13	578	90,539
Tefm	Transcription elongation factor, mitochondrial	13	95	77,574
Ccdc127	**SPRIZ**	**13**	**345**	**30,984**
Itpr1	IP3 receptor type 1	14	209	NA
Micu1	Mitochondrial calcium uptake 1 (MICU1)	17	NA	86,079
Tmx1	Thioredoxin-related transmembrane protein 1	21	3,514	NA
Mfn1	Mitofusin-1	26	171	39,396
Atad3a	AAA-domain containing 3A	26	1,621	413,258
Parl	Presenilin-associated rhomboid-like protease	29	240	20,515
Tfam	Transcription factor A, mitochondrial	34	44,926	1,502,919
Opa1	Optic atrophy 1	39	1,536	239,945
Synj2bp	Synaptojanin-2-binding protein / OMP25	43	8,689	326,554
Mfn2	Mitofusin-2	49	395	85,243
Vapb	VAPB	54	35,394	NA
Immt	Mic60 / Mitofilin	79	7,922	974,301
Mic19	CHCHD3	110	19,115	NA
Vdac1	Voltage-dependent anion channel 1	142	86,045	2,937,942
Slc25a1	Citrate carrier (CIC)	163	12,203	1,658,429
Sod2	Mn-superoxide dismutase (SOD2)	183	233,069	NA
MPC1	Mitochondrial pyruvate carrier subunit 1	303	18,153	84,012

Table S2. Comparative transcript and protein abundance of SPRIZ in mouse liver and HEK293 cells.

This table places SPRIZ abundance in context by comparing it to other mitochondrial proteins across independent quantitative datasets. In mouse liver, transcript levels (TPM) are from mouse liver transcriptomics from Anastasia et al (21), whereas protein abundance (iBAQ) was measured in wrappER-associated mitochondria-enriched fractions in this study. In HEK293 cells, absolute protein copy numbers per cell are reported from Morgenstern et al (40). SPRIZ is shown together with proteins of comparable abundance and selected high- and low-abundance reference proteins. The listed factors span major functional classes of mitochondrial membrane proteins and are not intended to represent a complete inventory.

Excel data file S1. Taxonomic distribution of SPRIZ homologs across eukaryotic proteomes.

Excel data file S2. Raw and analyzed proteomic data from wild-type and Ccdc127 knockout mouse liver mitochondria-enriched fractions.

Movie S1. All-atom molecular dynamics simulation (500 ns) of the OMM-anchored SPRIZ homodimer.

Movie S2. AlphaFold3-predicted structure of the SPRIZ homodimer C-terminal domain with the IMM-interfacing loops highlighted.

Movie S3. AlphaFold3-predicted structure of the SPRIZ homodimer C-terminal (IMM-interfacing) domain, including AlphaFold3-predicted phospholipid binding.

Movie S4. Coarse-grained molecular dynamics simulation (3 µs) of the SPRIZ C-terminal homodimer positioned above an IMM-like bilayer.

Movie S5. Three coarse-grained molecular dynamics simulations (3 µs each, under periodic boundary conditions) of the SPRIZ C-terminal homodimer carrying loop mutations positioned above an IMM-like bilayer.

Movie S6. Coarse-grained molecular dynamics simulation (5 µs) of the OMM-anchored wild-type SPRIZ homodimer engaging an IMM-like membrane.

Movie S7. All-atom molecular dynamics simulation (400 ns) of the OMM-anchored MARTINI homodimer.

REFERENCES

1. A. Suomalainen, J. Nunnari, Mitochondria at the crossroads of health and disease. *Cell* **187**, 2601–2627 (2024).

2. A. S. Monzel, J. A. Enríquez, M. Picard, Multifaceted mitochondria: Moving mitochondrial science beyond function and dysfunction. *Nat. Metab.* **5**, 546–562 (2023).

3. C. N. Cunningham, J. Rutter, 20,000 Picometers under the OMM: Diving into the vastness of mitochondrial metabolite transport. *EMBO Rep.* **21**, e50071 (2020).

4. S. A. Baker, J. Rutter, Metabolites as signalling molecules. *Nat. Rev. Mol. Cell Biol.* **24**, 355–374 (2023).

5. N. S. Chandel, Mitochondria. *Cold Spring Harb. Perspect. Biol.* **13**, a040543 (2021).

6. B. Cartes-Saavedra, A. Ghosh, G. Hajnóczky, The roles of mitochondria in global and local intracellular calcium signalling. *Nat. Rev. Mol. Cell Biol.* **26**, 456–475 (2025).

7. A. Oberst, C. Bender, D. R. Green, Living with death: The evolution of the mitochondrial pathway of apoptosis in animals. *Cell Death Differ.* **15**, 1139–1146 (2008).

8. M. Picard, O. S. Shirihai, Mitochondrial signal transduction. *Cell Metab.* **34**, 1620–1653 (2022).

9. S. Rath, R. Sharma, R. Gupta, T. Ast, C. Chan, T. J. Durham, R. P. Goodman, Z. Grabarek, M. E. Haas, W. H. W. Hung, P. R. Joshi, A. A. Jourdain, S. H. Kim, A. V. Kotrys, S. S. Lam, J. G. McCoy, J. D. Meisel, M. Miranda, A. Panda, A. Patgiri, R. Rogers, S. Sadre, H. Shah, O. S. Skinner, T. L. To, M. A. Walker, H. Wang, P. S. Ward, J. Wengrod, C. C. Yuan, S. E. Calvo, V. K. Mootha, MitoCarta3.0: An updated mitochondrial proteome now with sub-organelle localization and pathway annotations. *Nucleic Acids Res.* **49**, D1541–D1547 (2021).

10. M. Morgenstern, S. B. Stiller, P. Lübbert, C. D. Peikert, S. Dannenmaier, F. Drepper, U. Weill, P. Höß, R. Feuerstein, M. Gebert, M. Bohnert, M. van der Laan, M. Schuldiner, C. Schütze, S. Oeljeklaus, N. Pfanner, N. Wiedemann, B. Warscheid, Definition of a high-confidence mitochondrial proteome at quantitative scale. *Cell Rep.* **19**, 2836–2852 (2017).

11. E. V. Koonin, K. S. Makarova, I. B. Rogozin, L. Davidovic, M. C. Letellier, L. Pellegrini, The rhomboids: A nearly ubiquitous family of intramembrane serine proteases that probably evolved by multiple ancient horizontal gene transfers. *Genome Biol.* **4**, R19 (2003).

12. C. Glytsou, E. Calvo, S. Cogliati, A. Mehrotra, I. Anastasia, G. Rigoni, A. Raimondi, N. Shintani, M. Loureiro, J. Vazquez, L. Pellegrini, J. A. Enriquez, L. Scorrano, M. E. Soriano, Optic atrophy 1 is epistatic to the core MICOS component MIC60 in mitochondrial cristae shape control. *Cell Rep.* **17**, 3024–3034 (2016).

13. S. E. Calvo, V. K. Mootha, The mitochondrial proteome and human disease. *Annu. Rev. Genomics Hum. Genet.* **11**, 25–44 (2010).

14. Kondadi, Arun Kumar, Ruchika Anand, and Andreas S. Reichert. Cristae membrane dynamics–A paradigm change. *Trends Cell Biol.* **30**, 923–936 (2020).

15. O. Daumke, M. van der Laan, Molecular machineries shaping the mitochondrial inner membrane. *Nat. Rev. Mol. Cell Biol.* **26**, 706–724 (2025).

16. T. Bock-Bierbaum, K. Funck, F. Wollweber, E. Lisicki, K. von der Malsburg, A. von der Malsburg, J. Laborenz, J. K. Noel, M. Hessenberger, S. Jungbluth, C. Bernert, S. Kunz, D. Riedel, H. Lilie, S. Jakobs, M. van der Laan, O. Daumke, Structural insights into crista junction formation by the Mic60-Mic19 complex. *Sci. Adv.* **8**, eabo4946 (2022).

17. T. Stephan, C. Brüser, M. Deckers, A. M. Steyer, F. Balzarotti, M. Barbot, T. S. Behr, G. Heim, W. Hübner, P. Ilgen, F. Lange, D. Pacheu-Grau, J. K. Pape, S. Stoldt, T. Huser, S. W. Hell, W. Möbius, P. Rehling, D. Riedel, S. Jakobs, MICOS assembly controls mitochondrial inner membrane remodeling and crista junction redistribution to mediate cristae formation. *EMBO J.* **39**, e104105 (2020).

18. T. Schnyder, M. Rojo, R. Furter, T. Wallimann, The structure of mitochondrial creatine kinase and its membrane binding properties. *Mol. Cell. Biochem.* **133-134**, 115–123 (1994).

19. Stachowiak O, Schlattner U, Dolder M, Wallimann T. Oligomeric state and membrane binding behaviour of creatine kinase isoenzymes: Implications for cellular function and mitochondrial structure. *Mol. Cell. Biochem.* **184**, 141–151 (1998).

20. L. Kay, K. Nicolay, B. Wieringa, V. Saks, T. Wallimann, Direct evidence for the control of mitochondrial respiration by mitochondrial creatine kinase in oxidative muscle cells in situ. *J. Biol. Chem.* **275**, 6937–6944 (2000).

21. I. Anastasia, N. Ilacqua, A. Raimondi, P. Lemieux, R. Ghandehari-Alavijeh, G. Faure, S. L. Mekhedov, K. J. Williams, F. Caicci, G. Valle, M. Giacomello, A. D. Quiroga, R. Lehner, M. J. Miksis, K. Toth, T. Q. D. A. Vallim, E. V. Koonin, L. Scorrano, L. Pellegrini, Mitochondria-rough-ER contacts in the liver regulate systemic lipid homeostasis. *Cell Rep.* **34**, 108873 (2021).

22. B. Gilquin, E. Taillebourg, N. Cherradi, A. Hubstenberger, O. Gay, N. Merle, N. Assard, M.-O. Fauvarque, S. Tomohiro, O. Kuge, J. Baudier, The AAA+ ATPase ATAD3A controls mitochondrial dynamics at the interface of the inner and outer membranes. *Mol. Cell. Biol.* **30**, 1984–1996 (2010).

23. Y. Zhao, D. Hu, R. Wang, X. Sun, P. Ropelewski, Z. Hubler, K. Lundberg, Q. Wang, D. J. Adams, R. Xu, X. Qi, ATAD3A oligomerization promotes neuropathology and cognitive deficits in Alzheimer's disease models. *Nat. Commun.* **13**, 1121 (2022).

24. K. Can, A. Erstbetreuung, F. Liu, MitoMap-a three-dimensional landscape of mitochondrial architecture, thesis, Charité – Universitätsmedizin Berlin, Berlin, Germany (2025).

25. Y. Xia, Y. Zhang, Y. Sun, L. He, CCDC127 regulates lipid droplet homeostasis by enhancing mitochondria-ER contacts. *Biochem. Biophys. Res. Commun.* **683**, 149116 (2023).

26. V. Hung, P. Zou, H.-W. Rhee, N. D. Udeshi, V. Cracan, T. Svinkina, S. A. Carr, V. K. Mootha, A. Y. Ting, Proteomic mapping of the human mitochondrial intermembrane space in live cells via ratiometric APEX tagging. *Mol. Cell* **55**, 332–341 (2014).

27. C. Zarges, P. Schepsky, R. A. Rothemann, T. Bock-Bierbaum, A. von der Malsburg, L. Baumann, A. Trifunovic, O. Daumke, M. van der Laan, K. von der Malsburg, J. Riemer,

Topology control by a conserved cysteine pair in the OMM-protein CCDC127 enables MICOS interaction. bioRxiv 2025.12.12.693940 [Preprint] (2025); https://doi.org/10.64898/2025.12.12.693940.

28. S. Hassdenteufel, Y. Bykov, B. Dubreuil, K. Noll, O. Faust, R. Kamyshinsky, M. Itkin, N. Cohen, S. Malitsky, Y. Peleg, S. Albeck, K. von der Malsburg, D. Fass, M. van der Laan, M. Schuldiner, A multiplexed approach for genetic screening of human cells by electron microscopy uncovers a critical effector of mitochondrial cristae shape. bioRxiv 2025.11.15.688601 [Preprint] (2025); https://doi.org/10.1101/2025.11.15.688601.

29. T. Bock-Bierbaum, K. von der Malsburg, A. K. Natarajan, C. Zarges, K. C. Akkaya, A. Aladawi, K. Noll, S. Jungbluth, C. Schirra, Y. Zhu, N. Cremer, C. Bernert, F. Liu, M. Lehmann, J. Riemer, M. van der Laan, O. Daumke, Structural studies suggest CCDC127 as a novel membrane contact site protein in the mitochondrial intermembrane space. bioRxiv 2025.12.11.693622 [Preprint] (2025); https://doi.org/10.64898/2025.12.11.693622.

30. N. Ilacqua, I. Anastasia, D. Aloshyn, R. Ghandehari-Alavijeh, E. A. Peluso, M. C. Brearley-Sholto, L. V. Pellegrini, A. Raimondi, T. Q. de Aguiar Vallim, L. Pellegrini, Expression of Synj2bp in mouse liver regulates the extent of wrappER-mitochondria contact to maintain hepatic lipid homeostasis. *Biol. Direct* **17**, 37 (2022).

31. N. Ilacqua, I. Anastasia, A. Raimondi, P. Lemieux, T. Q. de Aguiar Vallim, K. Toth, E. V. Koonin, L. Pellegrini, A three-organelle complex made by wrappER contacts with peroxisomes and mitochondria responds to liver lipid flux changes. *J. Cell Sci.* **135**, jcs259091 (2021).

32. N. Ilacqua, I. Anastasia, L. Pellegrini, Isolation and analysis of fractions enriched in WrappER-associated mitochondria from mouse liver. *STAR Protoc.* **2**, 100752 (2021).

33. T. B. Ansell, R. A. Corey, L. V. Viti, M. Kinnebrew, R. Rohatgi, C. Siebold, M. S. P. Sansom, The energetics and ion coupling of cholesterol transport through Patched1. *Sci. Adv.* **9**, eadh1609(2023).

34. M. Chavent, A. L. Duncan, M. S. P. Sansom, Molecular dynamics simulations of membrane proteins and their interactions: From nanoscale to mesoscale. *Curr. Opin. Struct. Biol.* **40**, 8–16 (2016).

35. W. Pezeshkian, S. J. Marrink, Simulating realistic membrane shapes. *Curr. Opin. Cell Biol.* **71**, 103–111 (2021).

36. L. Borges-Araújo, I. Patmanidis, A. P. Singh, L. H. S. Santos, A. K. Sieradzan, S. Vanni, C. Czaplewski, S. Pantano, W. Shinoda, L. Monticelli, A. Liwo, S. J. Marrink, P. C. T. Souza, Pragmatic coarse-graining of proteins: Models and applications. *J. Chem. Theory Comput.* **19**, 7112–7135 (2023).

37. V. Corradi, B. I. Sejdiu, H. Mesa-Galloso, H. Abdizadeh, S. Y. Noskov, S. J. Marrink, D. P. Tieleman, Emerging diversity in lipid–protein interactions. *Chem. Rev.* **119**, 5775–5848 (2019).

38. P. C. T. Souza, R. Alessandri, J. Barnoud, S. Thallmair, I. Faustino, F. Grünewald, I. Patmanidis, H. Abdizadeh, B. M. H. Bruininks, T. A. Wassenaar, P. C. Kroon, J. Melcr, V. Nieto, V. Corradi, H. M. Khan, J. Domański, M. Javanainen, H. Martinez-Seara, N. Reuter, R. B. Best, I. Vattulainen, L. Monticelli, X. Periole, D. P. Tieleman, A. H. de Vries, S. J. Marrink, Martini 3: A general purpose force field for coarse-grained molecular dynamics. *Nat. Methods* **18**, 382–388 (2021).

39. J. Jumper, R. Evans, A. Pritzel, T. Green, M. Figurnov, O. Ronneberger, K. Tunyasuvunakool, R. Bates, A. Žídek, A. Potapenko, A. Bridgland, C. Meyer, S. A. A. Kohl, A. J. Ballard, A. Cowie, B. Romera-Paredes, S. Nikolov, R. Jain, J. Adler, T. Back, S. Petersen, D. Reiman, E. Clancy, M. Zielinski, M. Steinegger, M. Pacholska, T. Berghammer, S. Bodenstein, D. Silver, O. Vinyals, A. W. Senior, K. Kavukcuoglu, P. Kohli, D. Hassabis, Highly accurate protein structure prediction with AlphaFold. *Nature* **596**, 583–589 (2021).

40. M. Morgenstern, C. D. Peikert, P. Lübbert, I. Suppanz, C. Klemm, O. Alka, C. Steiert, N. Naumenko, A. Schendzielorz, L. Melchionda, W. W. D. Mühlhäuser, B. Knapp, J. D. Busch, S. B. Stiller, S. Dannenmaier, C. Lindau, M. Licheva, C. Eickhorst, R. Galbusera, R. M. Zerbes, M. T. Ryan, C. Kraft, V. Kozjak-Pavlovic, F. Drepper, S. Dennerlein, S. Oeljeklaus, N. Pfanner, N.

Wiedemann, B. Warscheid, Quantitative high-confidence human mitochondrial proteome and its dynamics in cellular context. *Cell Metab.* **33**, 2464–2483.e18 (2021).

41. M. K. Kucińska, J. Fedry, C. Galli, D. Morone, A. Raimondi, T. Soldà, F. Förster, M. Molinari, TMX4-driven LINC complex disassembly and asymmetric autophagy of the nuclear envelope upon acute ER stress. *Nat. Commun.* **14**, 3497 (2023).

42. S. Jo, T. Kim, V. G. Iyer, W. Im, CHARMM-GUI: A web-based graphical user interface for CHARMM. *J. Comput. Chem.* **29**, 1859–1865 (2008).

43. M. A. Lomize, I. D. Pogozheva, H. Joo, H. I. Mosberg, A. L. Lomize, OPM database and PPM web server: Resources for positioning of proteins in membranes. *Nucleic Acids Res.* **40**, D370–D376 (2012).

44. J. C. Phillips, D. J. Hardy, J. D. C. Maia, J. E. Stone, J. V. Ribeiro, R. C. Bernardi, R. Buch, G. Fiorin, J. Hénin, W. Jiang, R. McGreevy, M. C. R. Melo, B. K. Radak, R. D. Skeel, A. Singharoy, Y. Wang, B. Roux, A. Aksimentiev, Z. Luthey-Schulten, L. V. Kalé, K. Schulten, C. Chipot, E. Tajkhorshid, Scalable molecular dynamics on CPU and GPU architectures with NAMD. *J. Chem. Phys.* **153**, 044130 (2020).

45. J. Lee, X. Cheng, J. M. Swails, M. S. Yeom, P. K. Eastman, J. A. Lemkul, S. Wei, J. Buckner, J. C. Jeong, Y. Qi, S. Jo, V. S. Pande, D. A. Case, C. L. Brooks III, A. D. MacKerell, J. B. Klauda, W. Im, Charmm-gui input generator for namd, gromacs, amber, openmm, and charmm/openmm simulations using the charmm36 additive force field. *J. Chem. Theory Comput.* **12**, 405–413 (2016).

46. M. J. Abraham, T. Murtola, R. Schulz, S. Páll, J. C. Smith, B. Hess, E. Lindahl, GROMACS: High performance molecular simulations through multi-level parallelism from laptops to supercomputers. *SoftwareX* **1-2**, 19–25 (2015).

47. C. M. Brown, M. S. S. Westendorp, R. Zarmiento-Garcia, J. A. Stevens, B. M. H. Bruininks, S. L. Rouse, S. J. Marrink, T. A. Wassenaar, An integrative modelling approach to the mitochondrial cristae. *Commun. Biol.* **8**, 972 (2025).

48. C. Frezza, S. Cipolat, L. Scorrano, Organelle isolation: Functional mitochondria from mouse liver, muscle and cultured fibroblasts. *Nat. Protoc.* **2**, 287–295 (2007).

49. M. Triolo, S. Wade, N. Baker, M. Khacho, Evaluating mitochondrial length, volume, and cristae ultrastructure in rare mouse adult stem cell populations. *STAR Protoc.* **4**, 102107 (2023).

50. P. Kotchetkov, B. Lacoste, Volumetric analysis of the blood–brain barrier after ischemic stroke by electron tomography in mice. *Microcirculation* **32**, e70025 (2025).

51. Y. Gao, J. Lee, I. P. S. Smith, H. Lee, S. Kim, Y. Qi, J. B. Klauda, G. Widmalm, S. Khalid, W. Im, CHARMM-GUI supports hydrogen mass repartitioning and different protonation states of phosphates in lipopolysaccharides. *J. Chem. Inf. Model.* **61**, 831–839 (2021).

52. T. Darden, L. Perera, L. Li, L. Pedersen, New tricks for modelers from the crystallography toolkit: The particle mesh Ewald algorithm and its use in nucleic acid simulations. *Structure* **7**, R55–60 (1999).

53. S. Miyamoto, P. A. Kollman, Settle: An analytical version of the SHAKE and RATTLE algorithm for rigid water models. *J. Comput. Chem.* **13**, 952–962 (1992).

54. J.-P. Ryckaert, G. Ciccotti, H. J. C. Berendsen, Numerical integration of the cartesian equations of motion of a system with constraints: Molecular dynamics of n-alkanes. *J. Comput. Phys.* **23**, 327–341 (1977).

55. H. C. Andersen, Rattle: A “velocity” version of the shake algorithm for molecular dynamics calculations. *J. Comput. Phys.* **52**, 24–34 (1983).

56. R. J. Loncharich, B. R. Brooks, R. W. Pastor, Langevin dynamics of peptides: The frictional dependence of isomerization rates of N-acetylalanyl-N’-methylamide. *Biopolymers* **32**, 523–535 (1992).

57. J. C. Phillips, R. Braun, W. Wang, J. Gumbart, E. Tajkhorshid, E. Villa, C. Chipot, R. D. Skeel, L. Kalé, K. Schulten, Scalable molecular dynamics with NAMD. *J. Comput. Chem.* **26**, 1781–1802 (2005).

58. P. C. Kroon, F. Grünewald, J. Barnoud, M. van Tilburg, C. Brasnett, P. C. T. Souza, T. A. Wassenaar, S. J. Marrink, Martinize2 and Vermouth provide a unified framework for molecular topology generation. *eLife* **12**, RP90627 (2025).

59. M. Parrinello, A. Rahman, Polymorphic transitions in single crystals: A new molecular dynamics method. *J. Appl. Phys.* **52**, 7182–7190 (1981).

60. M. S. S. Westendorp, J. A. Stevens, B. M. H. Bruininks, S.-J. Marrink, Bentopy: Building molecular dynamics simulations with cellular complexity and scale., Zenodo (2024); https://doi.org/10.5281/ZENODO.13818758.

61. N. Michaud-Agrawal, E. J. Denning, T. B. Woolf, O. Beckstein, MDAnalysis: A toolkit for the analysis of molecular dynamics simulations. *J. Comput. Chem.* **32**, 2319–2327 (2011).

62. Brady Johnston, Pardhav Maradani, Johannes Elferich, Russell B. Davidson, Yuxuan Zhuang, Yinying Yao, zachcp, Thibault Tubiana, Rich, Patrick Kunzmann, Olive Laprevote, TheJeran, ludovic autin, Kai Niklas Spauszus, Domenico Marson, Blastorios, Alyssa Travitz, Marcelo C. R. Melo, Hampton Copeland, elija feigl, bion howard, Tyler Reddy, Prasun Kumar, Louis Colson, Joyce Kim, Jessica A. Nash, Jamie Rowe, James Hooker, Emmanuel Ferdman, Brener Ramos, BradyAJohnston/MolecularNodes: v4.5.10, version v4.5.10, Zenodo (2026); https://doi.org/10.5281/ZENODO.18385790.

63. T. Schmidt, P. Samaras, M. Frejno, S. Gessulat, M. Barnert, H. Kienegger, H. Krcmar, J. Schlegl, H.-C. Ehrlich, S. Aiche, B. Kuster, M. Wilhelm, ProteomicsDB. *Nucleic Acids Res.* **46**, D1271–D1281 (2018).

64. P. Samaras, T. Schmidt, M. Frejno, S. Gessulat, M. Reinecke, A. Jarzab, J. Zecha, J. Mergner, P. Giansanti, H.-C. Ehrlich, S. Aiche, J. Rank, H. Kienegger, H. Krcmar, B. Kuster, M. Wilhelm, ProteomicsDB: A multi-omics and multi-organism resource for life science research. *Nucleic Acids Res.* **48**, gkz974 (2019).

65. L. Lautenbacher, P. Samaras, J. Muller, A. Grafberger, M. Shraideh, J. Rank, S. T. Fuchs, T. K. Schmidt, M. The, C. Dallago, H. Wittges, B. Rost, H. Krcmar, B. Kuster, M. Wilhelm,

ProteomicsDB: Toward a FAIR open-source resource for life-science research. *Nucleic Acids Res.* **50**, D1541–D1552 (2022).

66. M. Wilhelm, J. Schlegl, H. Hahne, A. M. Gholami, M. Lieberenz, M. M. Savitski, E. Ziegler, L. Butzmann, S. Gessulat, H. Marx, T. Mathieson, S. Lemeer, K. Schnatbaum, U. Reimer, H. Wenschuh, M. Mollenhauer, J. Slotta-Huspenina, J.-H. Boese, M. Bantscheff, A. Gerstmair, F. Faerber, B. Kuster, Mass-spectrometry-based draft of the human proteome. *Nature* **509**, 582–587 (2014).
